# Structural-model-based genome mining can efficiently discover novel non-canonical terpene synthases hidden in genomes of diverse species†

**DOI:** 10.1039/d4sc01381f

**Published:** 2024-06-05

**Authors:** Tohru Abe, Haruna Shiratori, Kosuke Kashiwazaki, Kazuma Hiasa, Daijiro Ueda, Tohru Taniguchi, Hajime Sato, Takashi Abe, Tsutomu Sato

**Affiliations:** a Department of Life and Food Sciences, Graduate School of Science and Technology, Niigata University Ikarashi 2-8050, Nishi-ku Niigata 950-2181 Japan satot@agr.niigata-u.ac.jp; b Department of Electrical and Information Engineering, Graduate School of Science and Technology, Niigata University Ikarashi 2-8050, Nishi-ku Niigata 950-2181 Japan takaabe@ie.niigata-u.ac.jp; c Interdisciplinary Graduate School of Medicine and Engineering, University of Yamanashi 4-4-37 Takeda Kofu Yamanashi 400-8510 Japan hsato@yamanashi.ac.jp; d Frontier Research Center for Advanced Material and Life Science, Faculty of Advanced Life Science, Hokkaido University North 21 West 11 Sapporo 001-0021 Japan; e PRESTO, Japan Science and Technology Agency Kawaguchi Saitama 332-0012 Japan

## Abstract

Non-canonical terpene synthases (TPSs) with primary sequences that are unrecognizable as canonical TPSs have evaded detection by conventional genome mining. This study aimed to prove that novel non-canonical TPSs can be efficiently discovered from proteins, hidden in genome databases, predicted to have 3D structures similar to those of class I TPSs. Six types of non-canonical TPS candidates were detected using this search strategy from 268 genome sequences from actinomycetes. Functional analyses of these candidates revealed that at least three types were novel non-canonical TPSs. We propose classifying the non-canonical TPSs as classes ID, IE, and IF. A hypothetical protein MBB6373681 from *Pseudonocardia eucalypti* (PeuTPS) was selected as a representative example of class ID TPSs and characterized. PeuTPS was identified as a diterpene synthase that forms a 6/6/6-fused tricyclic gersemiane skeleton. Analyses of PeuTPS variants revealed that amino acid residues within new motifs [D(N/D), ND, and RXXKD] located close to the class I active site in the 3D structure were essential for enzymatic activity. The homologs of non-canonical TPSs found in this study exist in bacteria as well as in fungi, protists, and plants, and the PeuTPS gene is not located near terpene biosynthetic genes in the genome. Therefore, structural-model-based genome mining is an efficient strategy to search for novel non-canonical TPSs that are independent of biological species and biosynthetic gene clusters and will contribute to expanding the structural diversity of terpenoids.

## Abstract 2

Non-canonical terpene synthases with primary sequences that are unrecognizable as canonical terpene synthases could be discovered through structural-model-based genome mining.

## Graphical abstract

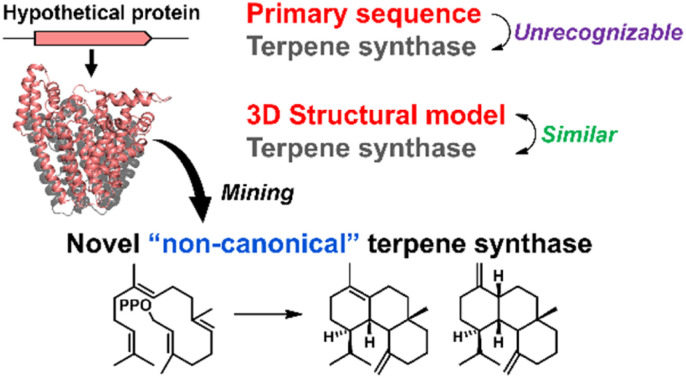

## Introduction

Terpenoids are one of the most diverse and abundant classes of natural products, comprising more than 80 000 compounds.^1^ Terpene synthases (TPSs; also referred to as terpene cyclases) are responsible for generating the structural diversity of cyclic skeletons and are classified into classes I and II based on differences in their cyclization initiation mechanisms (elimination of diphosphate or protonation of double bonds/epoxides, respectively) and active site motifs (DDXXD + NSE/DTE and DXDD).^2,3^ The active site motifs in class I TPSs, DDXXD and NSE/DTE, are involved in diphosphate group binding in the substrate *via* Mg^2+^ to eliminate the diphosphate group.^2,3^ The effector triad, composed of Arg, Asp, and Gly residues, is involved in substrate recognition and carbocation stabilization and is conserved in class I TPSs.^4,5^ Genome mining using sequence homology such as BLAST and Hidden Markov models, which are based on the overall and motif sequences of these enzymes, has led to the discovery of many novel terpenes from various organisms such as bacteria, fungi, and plants.^5–9^ In contrast, several non-canonical (or unconventional) TPSs with primary sequences that are unrecognizable as canonical classes I and II with no known active site motifs have been recently discovered.^10–16^ Notably, the 3D structures of two types of non-canonical TPSs (BalTS from *Bacillus alcalophilus* and AsR6s from *Acremonium strictum*) that catalyze class I type reactions are similar to those of class I TPSs (Table S1†).^13,16^ We have proposed classifying the BalTS homolog family into class IB.^13,14^ In the class IB TPS (BalTS), the six Asp residues in the DYLDNLXD and DY(F,L,W)IDXXED motifs exist in positions sterically close to those in class I, and these have been proven to be catalytic residues.^13,14^ Furthermore, in AsR6 (named class IC in this study), Asp and Lys residues are reported to be located in a position similar to that in class I and IB and are involved in diphosphate group recognition.^16^ The class IB and IC TPSs have been discovered through comprehensive analyses of gene-disruption-strain library and analysis of a related biosynthetic gene cluster, respectively.^12,15^ Further discoveries of non-canonical TPSs should contribute to expanding terpenoid diversity; however, the number of non-canonical TPSs that can be found by these methods is limited and efficient approaches that target a wide range of genes have not yet been developed. This study aimed to prove that novel non-canonical TPSs can be efficiently discovered from proteins, hidden in genome databases, predicted to have 3D structures similar to those of class I TPSs.

## Results and discussion

### Search for non-canonical TPS candidates

**Fig. 1:**
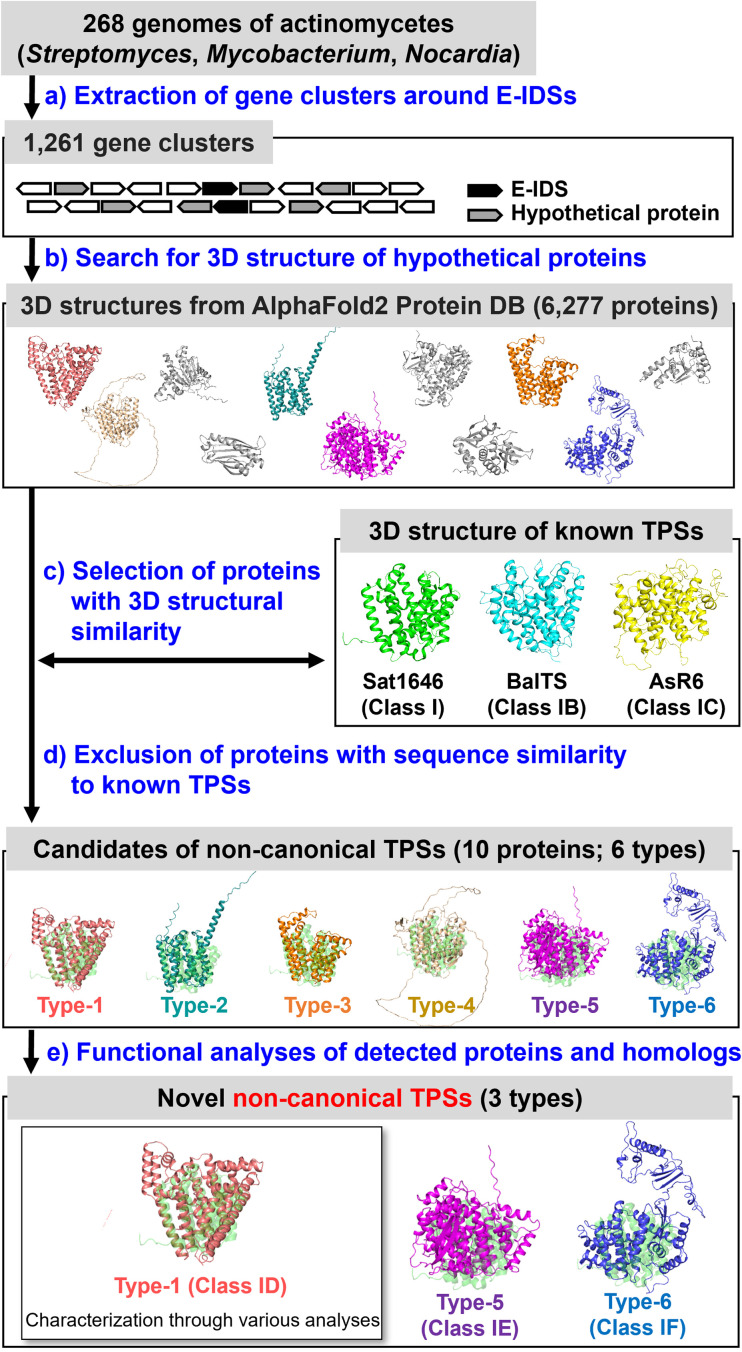
Strategy for structural-model-based genome mining of non-canonical TPSs. Initially, 819 complete genomes of actinomycetes (*Streptomyces*, *Mycobacterium*, and *Nocardia*) published in ENA/DDBJ/Genbank on 2023/06/12 were searched. However, 268 genomes for which the 3D structure models of the corresponding proteins were available from the Alphafold DB were used. The workflow detected ten proteins (5, 4, and 1 proteins from *Streptomyces*, *Mycobacterium*, and *Nocardia*, respectively) as non-canonical TPS candidates and classified them into six types (types 1–6) based on their sequence similarities. (a–d) The 3D models of the candidates were superposed with the class I TPS (Sat1646, green structure). Functional analyses of each candidate type (e) revealed that three (1, 5, and 6) were new non-canonical TPSs (classes ID, IE, and IF). In this study, seven of the type-1 (class ID) homologs were also subjected to the functional analyses.

We selected proteins with 3D structures similar to those of known class I and subclass TPSs (Sat1646 (ref. 17) from bacteria: class I; BalTS^13,14^ from bacteria: class IB; AsR6 (ref. 16) from fungi: class IC) as candidates for the non-canonical TPSs (Fig. 1a–c). To reduce the number of target proteins, we analyzed hypothetical proteins located within ±10 genes of *E*-isoprenyl diphosphate synthase (E-IDS), which biosynthesizes the substrate for TPSs on the genomes of actinomycetes of the genera *Streptomyces*, *Mycobacterium*, and *Nocardia* (Fig. 1a and b). This strategy should be applicable to various biological species and genome locations; however, to demonstrate the strategy efficacy, we selected the above restricted conditions. After excluding proteins with similarities to all known TPS sequences (Fig. 1d), we detected ten proteins as non-canonical TPS candidates from the genomes of 268 actinomycetes species (Tables S2, S3, Fig. S1†) and classified them into six types (types 1–6) based on their sequence similarities (*E* value < 0.1).

**Fig. 2:**
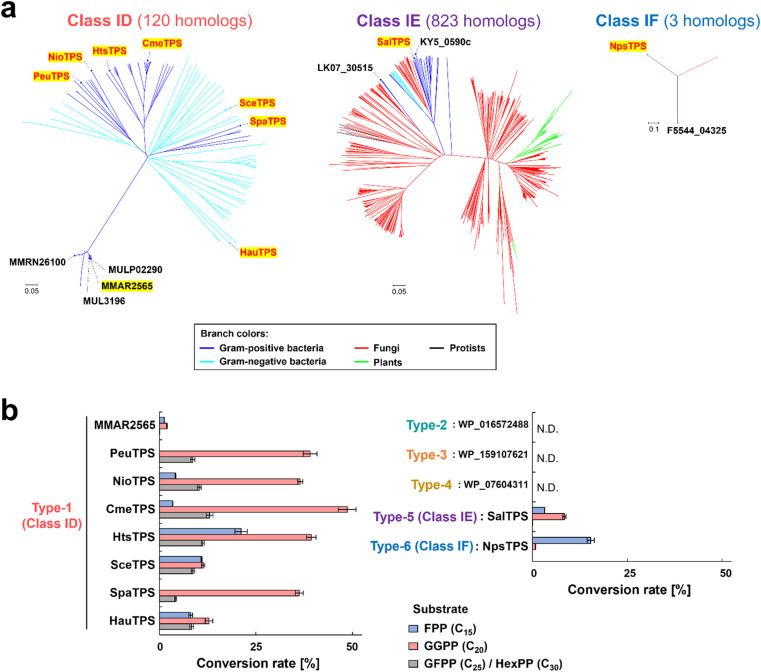
Phylogenic trees and enzymatic activities of non-canonical TPS candidates. (a) Phylogenetic tree of class ID, IE, and IF TPSs. The names of proteins detected from the structural-model-based search and their homologs subjected to functional analyses are shown in black and red, respectively. Proteins that exhibited TPS activities are highlighted in yellow. (b) Analysis of enzymatic activity of non-canonical TPS candidates using [^14^C]prenyl diphosphate substrates. A total of 50 μL of reaction mixture [25 mM MOPS-NaOH (pH 7.5), 10 mM MgCl_2_, 500 nM ^14^C-labeled substrates, and 100 nM purified enzyme] was incubated at 30 °C for 1 h. Conversion rates of substrates calculated from radioactivities on TLC are represented. Error bars indicate standard deviation (*n* = 3). N.D.: not detectable.

As a representative for analyzing type-1, MMAR2565 from *Mycobacterium marinum* discovered using the above search was selected. Regarding types 2–6, since the origin of detected genes was difficult to obtain, homologs from easily available bacterial strains or genomes were analyzed (Fig. 1e, Table S3, Fig. S1†). In addition, the functions of type-1 (MMAR2565) homologs were also analyzed to confirm that the homologs distributed in various bacterial species (*E* value < 10^−5^: currently 120 species; Fig. 2a) are non-canonical TPSs (Fig. 1e). Seven type-1 homologs (PeuTPS, NioTPS, CmeTPS, HtsTPS, SceTPS, SpaTPS, and HauTPS) from various phylogenetic groups, including non-actinomycetes (Fig. 2a, Table S3†), were selected for functional analysis.

### Functional analyses of non-canonical TPS candidates

Thirteen proteins (six types of non-canonical TPS candidates described above) were individually expressed as His-tagged fusion proteins in *Escherichia coli* and purified using a nickel affinity column (Fig. S2†). The enzymatic activity of each protein was evaluated using C_15_–C_30_ of the [^14^C]prenyl diphosphate substrate—specifically, *E*,*E*-farnesyl diphosphate (FPP, C_15_), *E*,*E*,*E*-geranylgeranyl diphosphate (GGPP, C_20_), and *ca.* 2 : 1 mixture of all-*E*-geranylfarnesyl diphosphate (GFPP, C_25_)/all-*E*-hexaprenyl diphosphate (HexPP, C_30_)—enzymatically synthesized by E-IDSs, and with the exception of three non-canonical TPS candidates (types 2–4), all proteins demonstrated activity toward prenyl diphosphate substrates to form dephosphorylated products, similar to class I type reactions (Fig. 2b and S3†). These proteins, with the exception of the less active MMAR2565 (Fig. 2b), were confirmed by gas chromatography-mass spectrometry (GC-MS) analysis to produce unknown terpenes (Fig. S4 and S5†), demonstrating their function as TPSs. In addition to diterpene synthases (type-1 of PeuTPS, NioTPS, CmeTPS, HtsTPS, SceTPS, SpaTPS, and HauTPS; type-5 of SalTPS), a sesquiterpene synthase (type-6 of NpsTPS) was found (Fig. 2b, S3–S5†). Since these findings were generated from only one test reaction performed under only one condition, to determine whether type 2–4 proteins exhibit enzymatic activities, further experiments under various reaction conditions need to be carried out. Here, we propose naming the three types of non-canonical TPSs (types-1, 5 and 6) classes ID, IE, and IF, respectively. BLAST searches found 120, 823, and 3 homologs of class ID, IE, and IF TPSs, respectively (Fig. 2a). Notably, the homologs of non-canonical TPSs discovered in this study exist in bacteria as well as in fungi, protists, and plants (Fig. 2a).

**Fig. 3:**
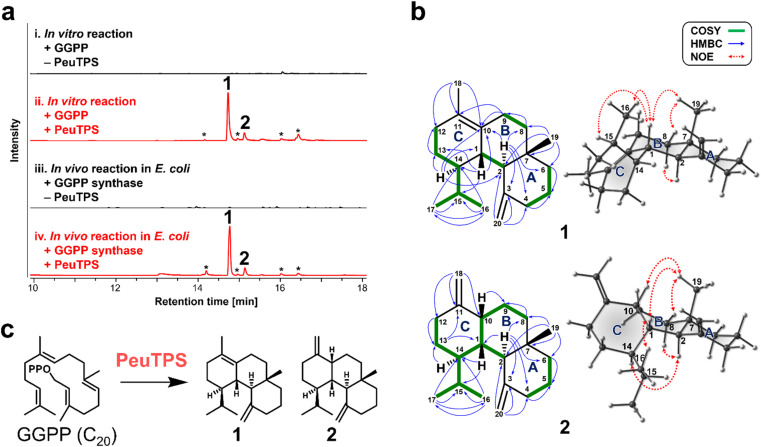
Analysis of PeuTPS products. (a) Gas chromatography-mass spectrometry analyses (total ion chromatograms) of PeuTPS products from GGPP by *in vitro* and *in vivo* reactions. (i) and (ii) Reaction products formed from GGPP in the absence (i) and presence (ii) of PeuTPS. (iii) and (iv) Metabolites of *E. coli* that expressed (iv) and did not express (iii) PeuTPS with GGPP synthase and mevalonate pathway enzymes. Asterisks indicate unidentified products. (b) Chemical structures of 1 and 2. The most stable 3D structures obtained by the conformational search are presented on the right. Characteristic 2D NMR correlations are shown. (c) Reaction catalyzed by PeuTPS.

To demonstrate that non-canonical TPSs absent in biosynthetic gene clusters can also be discovered with this strategy, the class ID homolog of PeuTPS that is not located near terpene biosynthetic gene clusters including E-IDS (Fig. S6†) was chosen as a representative example and characterized in detail using the most reactive GGPP substrate. GC-MS analysis revealed that PeuTPS produced two products (1 : 2 = *ca.* 85 : 15) from GGPP both *in vitro* and *in vivo* (Fig. 3a, S4, and S5†). Products, 1 and 2, were isolated from *E. coli* cultures co-expressing PeuTPS, GGPP synthase,^18^ and mevalonate pathway enzymes.^19^ Chemical structures of 1 and 2 were determined using mass spectrometry and nuclear magnetic resonance (NMR) (Fig. 3b, S7–S23†). Analyses of the HMBC and ^1^H,^1^H-COSY spectra showed that both 1 and 2 have 6/6/6-fused tricyclic skeletons and the structural difference between 1 and 2 is the position of the double bond in the C ring (Fig. 3b, S7, and S16†). The relative stereochemistry of the stereogenic centers at positions 1, 2, 7, and 14 in 1 was determined using NOE correlations (H1–H15, H1–H16, H1–H19, H8equatorial-H19, and H2–H8axial) (Fig. 3b and S7†). Since no H1–H14 correlation was observed in ^1^H,^1^H-COSY, the H1–H14 dihedral angle was inferred to be close to 90°. The NMR data and 3D structures calculated by the conformational search supported the relative stereochemistry of at position 14 in 1 (Fig. 3b, S7, and S15†). The relative stereochemistry of the stereogenic centers at positions 1, 2, 7, 10, and 14 in 2 was determined by NOE correlation (H1–H16, H1–H19, H10–H19, H8equatorial-H19, H2–H8axial, and H2–H14) (Fig. 3b and S16†). The absolute configuration of 1 was determined as 1*S*,2*R*,7*S*,14*S* using vibrational circular dichroism (VCD) spectroscopy (Fig. S24†). Owing to the low isolated amount, the stereochemistry of 2 could not be analyzed using VCD spectroscopy. However, since both 1 and 2 are produced by the same enzyme, PeuTPS, the absolute configuration of 2 should be similar to that of 1, as shown in Fig. 3b. Compounds 1 and 2 have not been reported as natural products. To the best of our knowledge, the only known natural products with the same cyclic skeleton (gersemiane skeleton) as 1 and 2 are 11 compounds found in corals, seagrass, liverwort, and marine cyanobacteria (Fig. S25†).^20–25^ We propose the names peugersemienes A and B for the compounds 1 and 2, respectively. This is the first report of TPS forming the gersemiane skeleton of 1 and 2 (Fig. 3c). We propose the reaction pathways of 1 and 2 based on DFT calculations (Fig. S26 and S27†); however, further analysis may be necessary to elucidate this mechanism. The production of 1 and 2 by *P. eucalypti* culture was not observed in this study, suggesting that either the PeuTPS gene might not be expressed under normal laboratory culture conditions or that both 1 and 2 might be further converted to unknown compounds. Several homologs of class ID, IE, and IF TPSs are clustered with genes of tailoring enzymes such as P450 on the genomes (Fig. S6†), suggesting that these TPSs may serve as core enzymes in undiscovered natural product biosynthesis. We are currently analyzing the structures of products synthesized by the class ID, IE, and IF TPSs other than PeuTPS and will report the diverse structures of these terpenes in the near future.

### 3D structural model, amino acid motifs, and metal ion requirements of PeuTPS

**Fig. 4:**
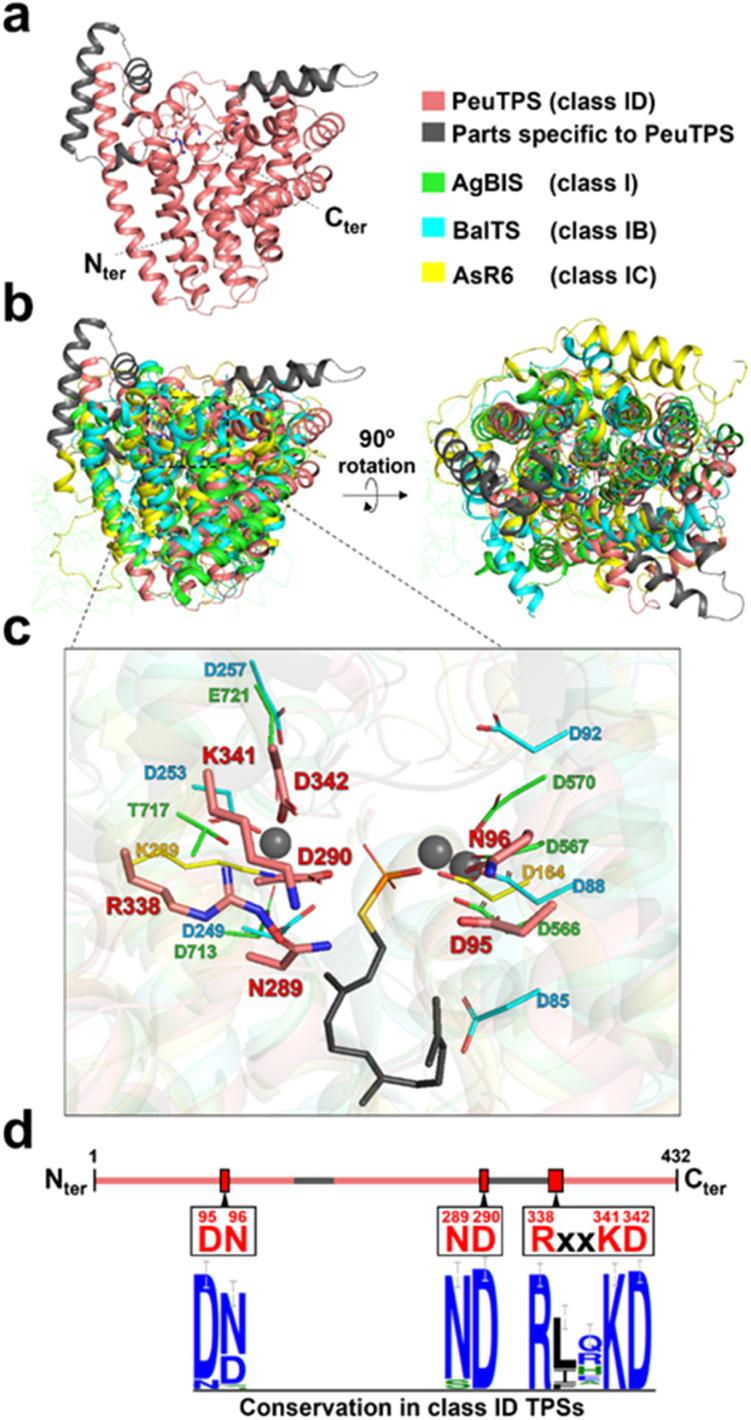
Structure of PeuTPS. (a) 3D structure of PeuTPS predicted by Alphafold2. (b) Superposition of the 3D structure of PeuTPS (pink and gray) with the class I TPS domain of AgBIS (green), BalTS (class IB; blue), and AsR6 (class IC; yellow). The model of PeuTPS has a sufficient confidence level in the region superposed with other TPSs (Fig. S1†). Additionally, the RMSD of PeuTPS and class I TPS domain of AgBIS (3.1) is comparable to that of the crystal structures, AgBIS-BalTS and AgBIS- AsR6 (both 3.7). (c) Proposed catalytic residues of PeuTPS (pink) superposed with that of AgBIS (green), BalTS (blue), and AsR6 (yellow). A side chain of farnesyl thiopyrophosphate and Mg^2+^ ion co-crystalized with AgBIS are represented as gray sticks and gray spheres, respectively. (d) Conservation of proposed catalytic residues in class ID TPSs. The height of the symbols in the sequence logos indicates the sequence conservation in 120 homologs at that position.

Among the 3D structure resolved proteins, the 3D structural model of PeuTPS (Fig. 4a, S1, and S28†) showed the highest similarity to that of the class I domain of α-bisabolene synthase from plant *Abies grandis* (AgBIS),^26^ which is composed of three domains (Table S4†). Superposition of the 3D structural model of PeuTPS with AgBIS (class I), BalTS (class IB), and AsR6 (class IC) indicated that the major α-helix components overlapped (Fig. 4b). However, PeuTPS has additional α-helix components that do not overlap with those in classes I, IB, or IC (Fig. 4b). Furthermore, the positions of the PeuTPS N- and C-termini differed from those of the others (Fig. S28†).

**Fig. 5:**
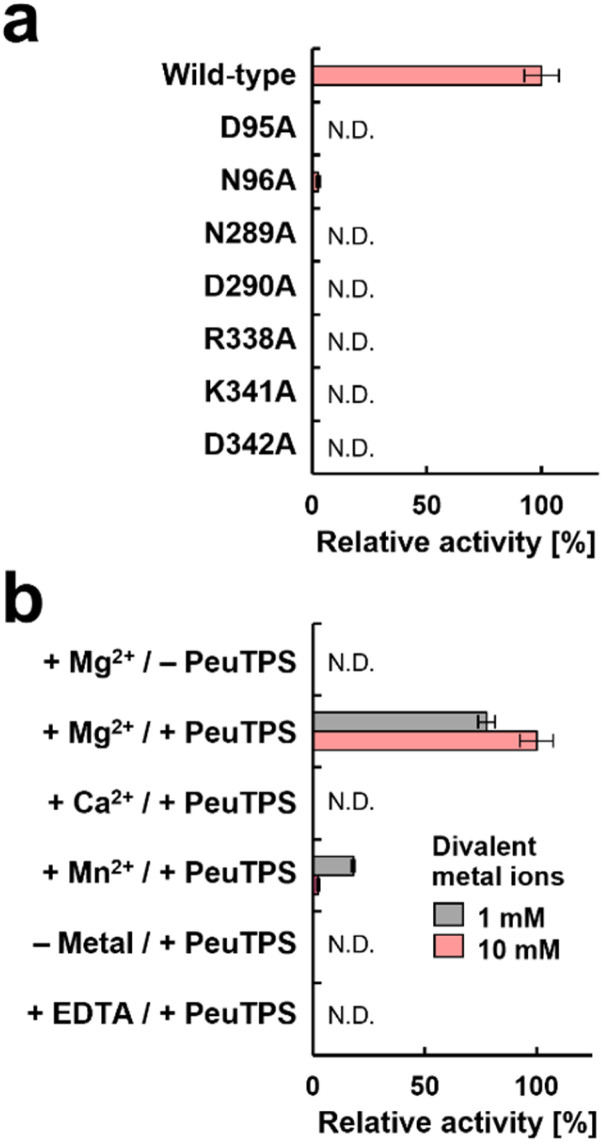
Enzymatic activities of wild-type and variant PeuTPSs in converting the GGPP substrate to produce 1 and 2. Quantification of the products was performed using GC. Product ratios were not changed in all reactions. Error bars indicate standard deviation (*n* = 3). N.D.: not detectable. (a) Comparison of enzymatic activities of variants with wild-type PeuTPS. (b) Analysis of requirements for divalent metal ions in the wild-type PeuTPS reaction. Concentrations of divalent metal ions were 1 or 10 mM. Reactions in the absence of divalent metal ions and in the presence of EDTA (10 mM) were also performed.

The catalytic residues in motifs of class I (DDXXD and NSE/DTE), IB [DYLDNLXD and DY(F,L,W)IDXXED], and Asp/Lys residues of class IC exist in similar positions in 3D structures (Fig. 4c). Seven residues (D95, N96, N289, D290, R338, K341, and D342) of PeuTPS are also located sterically close to the active site of known TPSs (Fig. 4c), and the same or similar residues were conserved in class ID TPSs (Fig. 4d and S29†). The new motifs [D(N/D), ND, and RXXKD] are clearly different from those of class I, IB, and IC TPSs (Fig. 4d). Therefore, we analyzed the enzymatic activities of PeuTPS variants in which these seven residues were individually replaced with Ala. All variants were inactivated or exhibited a significant reduction in activity (Fig. 5a), and the results were similar to previous analyses of BalTS (class IB) Ala variants targeting the six catalytic Asp residues.^13^ In addition, docking simulation of the GGPP substrate into the PeuTPS model structure suggested that the cavity around the residues of the motifs [D(N/D), ND, and RXXKD] is the only space in which the substrate can bind (Fig. S30†). Therefore, the position of the active site in PeuTPS as well as its overall 3D structure may be similar to that of classes I, IB, and IC. In addition, Mg^2+^ was essential for PeuTPS activity (Fig. 5b), suggesting that Mg^2+^ may contribute to the binding of the diphosphate group of the substrate, similar to most class I, IB, and IC TPSs. We propose that PeuTPS not only catalyzes diphosphate elimination but also protonation in the second step (Fig. S26†). However, its catalytic mechanism remains unclear. Furthermore, the effector triad strictly conserved in class I TPSs has not been found in the 3D structural model of PeuTPS (Fig. S31†). To elucidate the detailed catalytic mechanism of PeuTPS, the actual 3D structure of PeuTPS or its homolog should be evaluated in the future. In addition, we are currently analyzing the products of PeuTPS homologs in detail, and a comparison of their active site structures will help us understand the catalytic mechanism.

## Conclusions

In this study, we discovered the three novel types of non-canonical TPSs (classes ID, IE and IF) with the new strategy, structural-model-based genome mining. In particular, the class ID of PeuTPS has been characterized through various analyses. The class ID TPS genes (PeuTPS homologs) were found to be present in 120 species of bacterial genomes, and synthesized products different from those of PeuTPS (Fig. S3–S5†). Therefore, the strategy used in this study contributes to expanding the diversity of terpenoids. Since we only targeted 6277 proteins with available 3D structures from the Alphafold DB (Table S2†), further novel TPS candidates may be obtained by incorporating the 3D structure prediction step of unanalyzed hypothetical proteins (8457 proteins). Additionally, by broadening our focus to organisms beyond the genera *Streptomyces*, *Mycobacterium*, and *Nocardia* and exploring genes not located near the E-IDS, more non-canonical TPSs could be discovered. In fact, SceTPS and HauTPS are proteins from non-actinomycetes, and homologs of the non-canonical TPSs discovered in this study also exist in fungi, protists, and plants (Fig. 2a). In addition, the PeuTPS gene is not located near terpene biosynthetic genes, including E-IDS on the genome (Fig. S6†). Therefore, structural-model-based genome mining would be an efficient strategy to search for novel non-canonical TPSs that are independent of biological species and biosynthetic gene clusters and will contribute to expanding our understanding of the terpenoid world.

## Data availability

The datasets supporting this article have been uploaded as part of the ESI.†

## Author contributions

To. A. and T. S. designed the project. To. A., T. T., H. Sa., Ta. A., and T. S. wrote the manuscript. K. K. and Ta. A. performed bioinformatic analysis. K. H. and H. Sa. performed the DFT calculations. T. T. performed the VCD analysis. To. A., H. Sh., D. U., and T. S. performed all other wet experiments and associated data analyses. All the authors approved the final version of the manuscript.

## Conflicts of interest

There are no conflicts to declare.

## Supplementary Material

SC-015-D4SC01381F-s001
