# Supplementary material for: Structural-model-based genome mining can efficiently discover novel non-canonical terpene synthases hidden in genomes of diverse species
Source: Chem Sci. 2024 Jun 5;15(27):10402–7. doi: 10.1039/d4sc01381f (PMC11234867; doi:10.1039/d4sc01381f)
Supplement: SC-015-D4SC01381F-s001 [file SC-015-D4SC01381F-s001.pdf]

Electronic Supplementary Material (ESI) for Chemical Science.
This journal is © The Royal Society of Chemistry 2024

Supporting Information

Structural-model-based genome mining can efficiently discover novel non-canonical terpene synthases hidden in genomes of diverse species

Tohru Abe,[a] Haruna Shiratori,[a] Kosuke Kashiwazaki,[b] Kazuma Hiasa,[c] Daijiro Ueda,[a] Tohru Taniguchi,[d] Hajime Sato,[c,e]* Takashi Abe,[b]* Tsutomu Sato[a]*

Affiliation

[a] Department of Life and Food Sciences, Graduate School of Science and Technology, Niigata University, Ikarashi 2-8050, Nishi-ku, Niigata, Japan 950-2181, Japan.

[b] Department of Electrical and Information Engineering, Graduate School of Science and Technology, Niigata University, Ikarashi 2-8050, Nishi-ku, Niigata, Japan 950-2181, Japan.

[c] Interdisciplinary Graduate School of Medicine and Engineering, University of Yamanashi, 4-4-37 Takeda, Kofu, Yamanashi 400-8510, Japan.

[d] Frontier Research Center for Advanced Material and Life Science, Faculty of Advanced Life Science, Hokkaido University, North 21 West 11, Sapporo 001-0021, Japan.

[e] PRESTO, Japan Science and Technology Agency, Kawaguchi, Saitama 332-0012, Japan.

*** Corresponding authors**

Hajime Sato (hsato@yamanashi.ac.jp)

Takashi Abe (takaabe@ie.niigata-u.ac.jp)

Tsutomu Sato (satot@agr.niigata-u.ac.jp)

Table of Contents

Bioinformatics analysis S1

Genome sequences S1

Detection for gene clusters on genome focused on E-IDS S1

Search for non-canonical TPS candidates based on the similarity of protein 3D structure S1

Protein sequence analysis S1

Wet experiments S2

General procedure and materials S2

Cloning, expression, and purification of non-canonical TPS canditates S2

Enzymatic assay of non-canonical TPS candidates using [^{14}C]prenyl diphosphate substrates S3

Enzymatic assay of non-canonical TPS candidates using non-labeled prenyl diphosphate substrates S4

Production and isolation of **1** and **2** from *E. coli* cells expressing PeuTPS S4

Structural analyses of **1** and **2** S5

Analysis of the metabolites from *P. eucalypti* S5

Investigations of reaction pathway by DFT calculations S6

Computational details S6

Cartesian coordinates, energies, and imaginary frequencies S7-S14

Tables

Table S1. Z-scores and sequence identities of class I TPS (Sat1646) with class IB and IC TPSs S15

Table S2. The result of search workflow for non-canonical TPS candidates S16

Table S3. Non-canonical TPS candidates S17

Table S4. The top ten proteins with the highest structural similarity to PeuTPS S18

Table S5. Primers used in this study S19

Figures

Fig. S1. 3D structural model of non-canonical TPS candidates S20

Fig. S2. SDS-PAGE of recombinant proteins S21

Fig. S3. Enzymatic assay using [^{14}C]prenyl diphosphate substrates S22

Fig. S4. GC-MS total ion chromatograms of products formed by TPSs S23

Fig. S5. EI-MS spectra of products formed by TPSs S24

Fig. S6. The flanking region of the genes coding non-canonical TPSs S25

Fig. S7. NMR assignment of **1** measured in C_6D_6 S26

Fig. S8-14. NMR spectra of **1** S27-S33

Fig. S15. The most stable 3D structures of two isomers of **1** calculated by the conformational search S34

Fig. S16. NMR assignment of **2** measured in C_6D_6 S35

Fig. S17-23. NMR spectra of **2** S36-S37

Fig. S24. Calculated and observed VCD/IR spectra of **1** S43

Fig. S25. Examples of natural products possessing gersemiane skeleton S44

Fig. S26. Proposed mechanism for the formation of the PeuTPS products based on the DFT evaluation S45

Fig. S27. Mechanistic investigation using DFT calculation S46

Fig. S28. 3D structures of TPSs S47

Fig. S29. Sequence alignment of class ID TPSs analyzed in this study S48

Fig. S30. Docking model of PeuTPS with GGPP S49

Fig. S31. 3D view of the residues in PeuTPS corresponding to the effector triad in selinadiene synthase S50

References S51

Bioinformatics analysis

Genome sequences

The genera *Mycobacterium*, *Nocardia,* and *Streptomyces*, which belong to actinomycetes, were targeted in this study. Complete genomes were downloaded from ENA/DDBJ/Genbank on 2023/06/12. The annotated complete genomes included 393, 31, and 395 for the genera *Mycobacterium*, *Nocardia*, and *Streptomyces,* respectively.

Detection for gene clusters on genome focused on E-IDS

To search for non-canonical TPSs around E-IDS, we performed InterProScan (version 5.26-65.0, https://www.ebi.ac.uk/interpro/about/interproscan/)[1] using Pfam[2] as a reference database for protein sequences of each genome. E-IDS was assigned as IPR000092 in the Pfam database (step a). When similarity to this enzyme was identified, a total of 21 genes were extracted as a candidate gene cluster for terpene biosynthetic enzymes, comprising 10 genes before and after the enzyme with which similarity was found (step a). In addition, when multiple E-IDSs were detected within a gene cluster, the 10 most upstream and downstream genes, each, were defined as the gene cluster.

Search for non-canonical TPS candidates based on the similarity of protein 3D structure

A 3D structure information of hypothetical proteins (annotation on ENA/DDBJ/Genbank) detected in the gene cluster was obtained from the Alphafold Protein Structure Database (https://www.alphafold.ebi.ac.uk/)[3] (step b). Hypothetical proteins that were not registered in the database at the time of the study were excluded from this analysis. To select the hypothetical proteins with 3D structures similar to known TPSs, comparing 3D structures were performed using DaliLite.v5 (http://ekhidna2.biocenter.helsinki.fi/dali/)[4] (step c). As the query protein 3D structure of known TPSs (Sat1646: class I, BalTS: class IB, AsR6: class IC), we used the three protein 3D structures (PDB accession numbers: 5YO8, 7E4O, 7OC5) registered in PDB (Protein Data Bank).[5]

Proteins that showed DALI's Z-score of 5 or higher with either 5YO8, 7E4O, or 7OC5 were extracted (step c). Finally, a BLAST search and an InterProScan were performed manually, and proteins that are unrecognizable as known TPSs homologs (IPR008949, Isoprenoid synthase domain superfamily; IPR019712, Tetraprenyl-β-curcumene synthase YtpB-like; IPR044878, UbiA prenyltransferase superfamily) were defined as final novel non-canonical TPS candidates (step d). Moving step d to before step b may reduce unnecessary computational efforts.

Protein sequence analysis

The homolog search was performed against the non-redundant protein sequence database (National Center for Biotechnology Information) using PSI-BLAST. Multiple alignment was generated using clustal Omega. The sequence logos were generated with the aligned sequences of 120 class ID TPS homologs using the WebLogo online tool (https://weblogo.threeplusone.com/). Phylogenetic tree was constructed by the neighbor-joining method.

Wet experiments

General procedure and materials

DMAPP (dimethylallyl diphophate), FPP, and GGPP were prepared from 3-methylbut-2-en-1-ol, *E*,*E*-farnesol (FOH), and *E*,*E*,*E*-geranylgeraniol (GGOH), as described by Davisson *et al.*,[6] [1-^{14}C]IPP (55 mCi·mmol^{-1}) was purchased from American Radiolabeled Chemicals (Maryland Heights, MO, USA), and potato acid phosphatase was purchased from Sigma- Aldrich (St Louis, MO, USA). All other chemicals were of analytical grade. Gas chromatography-mass spectrometry (GC-MS) was performed on a JMS-T100GCV spectrometer (JEOL, Tokyo, Japan) equipped with a DB-1 capillary column (30 m×0.25 mm×0.25 μm) in electron ionization mode and operated at 70 eV. Gas chromatography (GC) analysis was performed on a Shimadzu GC-2014 chromatograph (Shimazu, Kyoto, Japan) equipped with a flame ionization detector and a DB-1 capillary column (30 m×0.25 mm×0.25 μm; J&W Scientific, Inc.). HPLC was performed on an LC-20AD chromatograph with SPD-20A UV-detector (Shimazu) at 210 nm. NMR spectrum was acquired using a Varian NMR System 700 at 700 MHz. The specific rotation was measured using a Horiba SEPA300 polarimeter. *Escherichia coli* JM109 was used for sequencing analysis, and *E. coli* BL21 (DE3) was used for expression of the genes. The genomic DNA of *Mycobacterium marinum* M (= Aronson BAA-535) was obtained from American Type Culture Collection. The strains *Pseudonocardia eucalypti* JCM 18303, *Streptomyces noursei* Brown *et al.* 1953 JCM5054, *Streptomyces ardesiacus* JCM5815, *Streptomyces amritsarensis* JCM19660, *Streptomyces alanosinicus* JCM4714, and *Nocardia pseudobrasiliensis* JCM9894 were obtained from RIKEN Japan Collection of Microorganisms.

Cloning, expression, and purification of non-canonical TPS candidates

The method is based on the method described in Ref.7. Among the proteins of each type candidates obtained from the homolog search, except MMAR2565, the bacterial strain available from RIKEN Japan Collection of Microorganisms were used as gene sources. The gene encoding MMAR2565 was amplified from the genome using primers listed in Table S5. Amplified PCR fragment was digested at NdeI/KpnI sites and inserted into the same sites in the pCold I vector (Takara), followed by sequence confirmation. Genes encoding type 1 candidates (PeuTPS, NioTPS, CmeTPS, HtsTPS, SceTPS, SpaTPS, and HauTPS) were synthesized by Genewiz (Morrisville, NC, USA) using codon-optimized sequences for *E. coli* and inserted into the NdeI/BamHI sites of pCold I. Genes encoding candidates of type 2-6 (WP_016572488, WP_159107621, WP_076043115, SalTPS, NpsTPS) were amplified by colony PCR using primers listed in Table S5. These amplified PCR fragments were digested at NdeI/EcoRI sites and inserted into the same sites in the pET28c vector (Novagen), followed by sequence confirmation. Plasmids expressing variants of PeuTPS were constructed using Quik Change Site-directed Mutagenesis Kit (Agilent Technologies, Santa Clara, CA, USA) and primers listed in Table S5. For the expression of recombinant proteins except MMAR2565, *E. coli* BL21(DE3) was transformed with the respective pCold I or pET28c derivatives and cultured at 37°C in 1 L of LB medium with antibiotics [ampicillin (100 μg·L^{-1}) for pCold I derivatives; kanamycin (25 μg·L^{-1}) for pET28c derivatives]. The culture was continued until the optical density at 600 nm reached *ca.* 0.6, followed by cultivation at 25°C for 16 h after the addition of IPTG (0.1 mM for pCold I derivatives; 0.5 mM for pET28c derivatives). For the expression of recombinant MMAR2565, *E. coli* BL21(DE3) was transformed with the pCold I derivative and a chaperon-expression plasmid pG-KJE8 (Clontech, Palo Alto, CA, USA). The culture was carried out at 37°C in 1 L

of LB medium with ampicillin (100 μg·L^{-1}), chloramphenicol (25 μg·L^{-1}), L-arabinose (0.5 g·L^{-1}), and tetracycline (5 ng·L^{-1}). Upon reaching an optical density at 600 nm of *ca*. 0.6, the cultivation was continued at 15°C for 24 h following the addition of IPTG (0.1 mM). Cells expressing the recombinant protein were harvested by centrifugation and disrupted by sonication in 25 mL of buffer A [20 mM Tris/HCl (pH 7.9) and 300 mM NaCl] containing 10 mM imidazole between 4 and 10°C. The homogenate was centrifuged at 18,270 x g for 20 min to prepare the supernatant containing soluble His-tagged fusion protein, which was loaded into a nickel-nitrilotriacetic acid agarose column (1 mL; Qiagen, Hilden, Germany), followed by washing with 10 mL of buffer A containing 10 mM imidazole and then 10 mL of buffer A containing 50 mM imidazole. The purified protein was eluted with 3 mL of buffer A, containing 250 mM imidazole, and then buffer-exchanged into buffer B [25 mM MOPS-NaOH (pH 7.5) and 150 mM NaCl] by gel-filtration chromatography using a Sephadex G-10 column (GE Healthcare, Chicago, IL, USA). The expression and purification of recombinant proteins were analyzed by SDS/PAGE on 10% gels.

Enzymatic assay of non-canonical TPS candidates using [^{14}C]prenyl diphosphate substrates

Enzymes used to synthesize [^{14}C]prenyl diphosphate substrates were prepared according to previously described methods.[7,8] [^{14}C]FPP was synthesized in 200 μL reaction mixtures containing 50 mM MOPS-NaOH (pH 7.9), 1 mM $MgCl_2$ and 20 μM DMAPP, 10 μM [^{14}C]IPP, and 5 μM Rv2173 from *Mycobacterium tuberculosis*[7] by incubating at 37°C for 30 min. [^{14}C]GGPP was synthesized in 200 μL reaction mixtures containing 50 mM MOPS-NaOH (pH 7.9), 1 mM $MgCl_2$ and 20 μM DMAPP, 20 μM [^{14}C]IPP, and 5 μM Mvan3536 from *M. vanbaalenii*[7] by incubating at 28°C for 30 min. [^{14}C]GFPP/HexPP was synthesized in 200 μL reaction mixtures containing 25 mM Tris-HCl(pH 8.5), 25 mM NH_4Cl, 1 mM $MgCl_2$ and 20 μM FPP, 5 μM [^{14}C]IPP, 5 μM Bcl-EIDS2L, and 5 μM Bcl-EIDS2S from *Bacillus clausii*[8], by incubating at 37°C for 5 min. After the reaction, the reaction mixture was washed with ethyl acetate and extracted with 1-butanol saturated with water, and the organic layer was washed with water. The concentration of the [^{14}C]prenyl diphosphates was determined by measuring radioactivity with a TriCarb2910TR liquid scintillation counter (PerkinElmer, Waltham, MA, USA). The carbon chain length of synthesized [^{14}C]prenyl diphosphates was confirmed by comparison with authentic FOH, GGOH, all-*E*-geranylfarnesol (GFOH), and all-*E*-hexaprenol (HexOH), using the reversed-phase RP-18 plate (Merck) after enzymatic dephosphorylation at 37°C for 16 h in the following mixture as reported previously[9]: 60% (*v*/*v*) methanol, 0.1 M acetate buffer (pH 5.6), 0.1% Triton X-100, 1 mg·mL^{-1} potato acid phosphatase. Authentic FOH and GGOH were commercially available, and GFOH and HexOH were previously synthesized.[10]

The reaction mixture used for the terpene synthase assay contained 25 mM MOPS-NaOH (pH 7.5), 10 mM $MgCl_2$, 500 nM ^{14}C-labeled substrates, and 100 nM purified enzyme in a total volume of 50 μL. The reaction was carried out at 30°C for 1 h and terminated by adding 50 μL of methanol. The product was extracted with 200 μL of *n*-hexane. The extract was concentrated and analyzed by thin layer chromatography (TLC) using a solvent system of *n*-hexane-ethyl acetate (100:20). Radioactive spots were detected with an image analyzer (FLA-9000; Fujifilm, Tokyo, Japan). The conversion rates were calculated as radioactivities of the products on TLC relative to that of the alcohol products that were quantitatively dephosphorylated from substrates by acid phosphatase described above.

Enzymatic assay of non-canonical TPS candidates using non-labeled prenyl diphosphate substrates

The reaction mixture typically contained 25 mM MOPS-NaOH (pH 7.5), 10 mM $MgCl_2$, 100 µM GGPP, and 1 µM purified recombinant protein in a total volume of 500 µL. The reaction was carried out at 30°C for 1 h (for quantitative analyses by GC) or 16 h (for qualitative analyses by GC-MS) and terminated by adding 500 µL methanol. The product was extracted with *n*-hexane (2 mL×3). The extracts were concentrated and analyzed by GC-MS (injection temperature, 230°C; oven temperature, from 60 to 300°C at increments of 10°C min^{-1}). Quantitative analysis of products of PeuTPS and its variants were performed GC under the same condition as GC-MS.

Production and isolation of 1 and 2 from *E. coli* cells expressing PeuTPS

To in vivo production of **1** and **2** in *E. coli*, PeuTPS gene was introduced to NdeI/BamHI sites of pET28c vector from pCold I derivative. Further, pCDF-Mvan3536, the plasmid expressing GGPP synthase, was constructed by the introduction of amplified Mvan3536 gene by PCR (primers are listed in Table S5) from previously constructed pCold I derivative[7] to NcoI/BamHI sites of pCDFduet-1 vector. The plasmid expressing mevalonate pathway enzymes and FPP synthase, pJBEI2997, was purchased from Addgene. *E. coli* BL21(DE3) was transformed by the pET28c-PeuTPS with pJBEI2997 and pCDF-Mvan3536. The strain was cultured at 37°C in 6 L of LB with kanamycin (25 µg·L-1), chloramphenicol (25 µg·L^{-1}), and streptomycin (50 µg·L^{-1}), and until an optical density at 600 nm reached *ca.* 0.6, which was followed by cultivation at 30°C for 72 h after the addition of IPTG (0.5 mM). The culture was separated into cells and broth by centrifugation. Cells were lyophilized, and the metabolites were extracted with methanol (600 mL×3). The extract was concentrated to 600 mL and further extracted with *n*-hexane (600 mL×3). The metabolites in the broth were extracted with *n*-hexane (3 L×3) after adding 1.2 L of acetone. The PeuTPS products were detected in extracts from cells and broth at a ratio of *ca.* 3:1. The extract (549.2 mg) from cells and broth was partially purified by silica gel (30 g) column chromatography with *n*-hexane, and 13 mg of the fraction (33.1 mg) was subjected to silver ion HPLC (Silver column KANTO, 4.6 × 250 mm, 5 µm; KANTO CHEMICAL CO., INC.) with *n*-hexane-acetonitrile (100:0.02, 0.5 mL·min^{-1}), yielding pure compound **1** (oil; 11.4 mg; retention time: 6.0 min) and compound **2** (oil; 1.3 mg; retention time: 8.5 min).

Structural analyses of compounds 1 and 2

The structure of **1** was determined by MS (Fig. S5) and NMR (Fig. S7–S14). HR-EI-MS detected *m*/*z* 272.2486 $[M]^+$ (calculated 272.2499 for $[C_{20}H_{32}]^+$). $[\alpha]^{20}_D$ = -20.56 (*c*= 0.11 in $CHCl_3$).

The structure of **2** was determined by MS (Fig. S5) and NMR (Fig. S16–S23). HR-EI-MS detected *m*/*z* 272.2487 $[M]^+$ (calculated 272.2499 for $[C_{20}H_{32}]^+$). $[\alpha]^{20}_D$ = -35.08 (*c*= 0.013 in $CHCl_3$).

VCD and IR spectra were recorded for a 0.9 M $CDCl_3$ solution of **1** placed in a 50-μm BaF_2 cell. These spectra were measured on a JASCO FVS-6000 spectrometer equipped with an optical filter that passes through 2200-850 cm^{-1} light and a MCT-V detector at a resolution of 4 cm^{-1} under ambient temperature. VCD spectra were recorded for 3000 scans, while IR spectra were recorded for 16 scans. Both spectra were corrected by solvent spectra obtained under the identical measurement conditions.

Theoretical calculations of VCD and IR spectra started with molecular mechanics conformational search of **1** using CONFLEX 9 software.[11] Obtained geometries within 7.5 kcal/mol from the most stable were optimized at DFT/B3LYP/6-311++G(d,p) using PCM for chloroform on Gaussian 16 program.[12] The VCD and IR spectra of the resultant two stable conformers within ΔE 2.0 kcal/mol from the most stable were calculated at the same level of theory. The calculated frequencies, dipole strengths and rotational strengths were converted to VCD and IR spectra on GaussView 6 software using a peak half-width at half height of 4 cm^{-1}. The calculated frequencies were scaled with a factor of 0.98. The VCD and IR spectra of each conformer were averaged based on its Boltzmann populations simulated at 298 K.

Analysis of the metabolites from *P. eucalypti*

P. eucalypti was cultivated in 42 yeast-starch medium (2.0 g yeast extract, 10 g soluble starch, dissolved in 1 L distilled water, pH 7.3) at 28 °C. For agar plates, agar (15.0 g/L) was added to the medium. After 14 days of cultivation in both liquid and agar medium, the metabolites were extracted and then analyzed using GC-MS by the same procedure as described above.

Investigations of reaction pathway by DFT calculations

Computational details

All calculations were carried out using the Gaussian 16 package.[12] Structure optimizations were performed with the M06-2X[13] density functional theory method and the 6-31+G(d,p) basis set without any symmetry restrictions. M06-2X was selected because of its accuracy in calculating terpene-forming reactions and its proven track record of being used in previous studies of reaction mechanism analysis.[14,15] Vibrational frequency calculations at the same level of theory with optimization were performed to verify that each local minimum has no imaginary frequency and that each transition states (**TS**) has only a single imaginary frequency. Conformational search was done with the Spartan'20 program.[16] Intrinsic reaction coordinate (IRC) calculations[17-20] for all **TSs** were performed with GRRM11[21] based on Gaussian 16. The Gibbs free energies (G_{rel}) were used for the discussion.

Cartesian coordinates, energies, and imaginary frequencies.

Compound_1_stable_conformer

C	-0.33000000	0.29400000	0.08300000
C	1.07700000	-0.39400000	0.10100000
C	1.70500000	-0.73000000	1.44800000
C	2.87700000	-1.68000000	1.31300000
C	3.97200000	-1.05600000	0.42100000
C	3.39400000	-0.54100000	-0.90600000
C	2.16100000	0.38900000	-0.74700000
C	1.60100000	0.70400000	-2.15300000
C	0.22000000	1.38700000	-2.12700000
C	-0.74800000	0.50100000	-1.37700000
C	-1.79200000	-0.13100000	-1.95300000
C	-2.62100000	-1.15300000	-1.20200000
C	-1.95900000	-1.62200000	0.09300000
C	-1.42600000	-0.43400000	0.91600000
C	-2.55400000	0.53800000	1.38600000
C	-2.04900000	1.58500000	2.39700000
C	-3.74400000	-0.20700000	2.02000000
C	-2.25500000	0.07400000	-3.37800000
C	2.59000000	1.70900000	-0.06900000
C	1.38800000	-0.18300000	2.62900000
H	-2.80200000	-2.01300000	-1.86200000
H	-1.11800000	-2.28200000	-0.15500000
H	-2.65200000	-2.23000000	0.68300000
H	-0.96500000	-0.84300000	1.82100000
H	-0.19400000	1.29600000	0.50800000
H	-3.61900000	-0.73300000	-1.00000000
H	-2.92500000	1.08000000	0.50600000
H	-3.41300000	-0.82900000	2.86200000
H	-4.47800000	0.50800000	2.40900000
H	-4.26600000	-0.85500000	1.31100000
H	-2.85600000	2.27600000	2.66500000
H	-1.71000000	1.10300000	3.32200000
H	-1.22300000	2.19100000	2.01200000
H	-2.04900000	-0.81400000	-3.99000000
H	-3.34400000	0.21700000	-3.40000000
H	-1.80300000	0.93400000	-3.87400000
H	-0.10200000	1.58000000	-3.15300000
H	0.29700000	2.36700000	-1.63600000
H	2.32500000	1.32900000	-2.69500000
H	1.50600000	-0.23300000	-2.71900000
H	0.95100000	-1.35300000	-0.42400000
H	4.17300000	-0.01400000	-1.47300000
H	3.09400000	-1.40300000	-1.52000000
H	3.00600000	1.54900000	0.92700000
H	3.35300000	2.20700000	-0.68000000
H	1.75400000	2.40700000	0.04200000
H	4.75700000	-1.79500000	0.22000000
H	4.45100000	-0.23500000	0.96800000
H	2.53500000	-2.61900000	0.85200000
H	3.28500000	-1.93300000	2.29800000
H	0.59300000	0.54400000	2.74700000
H	1.93600000	-0.45100000	3.53000000

Compound_2_stable_conformer

C	-0.46300000	0.19700000	0.42600000
C	0.90200000	-0.36300000	-0.06100000
C	1.47100000	-1.54100000	0.70700000
C	2.57300000	-2.24400000	-0.05100000
C	3.69000000	-1.25900000	-0.42700000
C	3.12100000	-0.03200000	-1.14400000
C	2.03400000	0.68800000	-0.31900000
C	1.41300000	1.80200000	-1.16900000
C	0.24400000	2.52700000	-0.47400000
C	-0.41200000	1.72500000	0.67600000
C	-1.77700000	2.29600000	0.99000000
C	-2.87000000	1.92000000	0.02600000
C	-2.94000000	0.39500000	-0.08700000
C	-1.60400000	-0.20900000	-0.54100000
C	-1.71300000	-1.74300000	-0.73600000
C	-2.48300000	-2.09300000	-2.01400000
C	-2.32800000	-2.47000000	0.46500000
C	-1.99600000	3.05100000	2.06900000
C	2.68800000	1.26600000	0.95300000
C	1.12800000	-1.91900000	1.94100000
H	-2.66400000	2.34300000	-0.96700000
H	-3.74100000	0.10800000	-0.77900000
H	-3.20500000	-0.01000000	0.89800000
H	-1.36000000	0.22400000	-1.52800000
H	-0.71300000	-0.24500000	1.39800000
H	-3.82800000	2.33200000	0.36000000
H	-0.69800000	-2.14000000	-0.85900000
H	-3.39900000	-2.25600000	0.55500000
H	-2.21700000	-3.55200000	0.34400000
H	-1.84200000	-2.19300000	1.40500000
H	-2.49600000	-3.17600000	-2.17200000
H	-3.52500000	-1.75900000	-1.96000000
H	-2.02500000	-1.62900000	-2.89400000
H	-2.97800000	3.46500000	2.28100000
H	-1.19800000	3.28000000	2.77100000
H	-0.51000000	2.78600000	-1.22500000
H	0.57800000	3.48100000	-0.05100000
H	2.18900000	2.52400000	-1.45400000
H	1.05900000	1.34400000	-2.10400000
H	0.72000000	-0.74700000	-1.07700000
H	3.92400000	0.67800000	-1.37800000
H	2.68700000	-0.34700000	-2.10400000
H	2.75800000	0.52100000	1.75200000
H	3.70400000	1.60600000	0.72300000
H	2.14800000	2.12800000	1.34700000
H	4.42900000	-1.75700000	-1.06400000
H	4.21600000	-0.94700000	0.48400000
H	2.15100000	-2.66600000	-0.97700000
H	2.97300000	-3.07800000	0.53500000
H	0.36500000	-1.41400000	2.52500000
H	1.62100000	-2.76300000	2.41500000
H	0.19900000	1.86500000	1.57200000

A

Sum of electronic and thermal Free Energies = -780.915922

Number of imaginary frequensies = 0

C	-2.07668300	-1.54322800	1.10723700
C	-1.00855600	-2.10499600	0.20693700
C	0.29034300	-2.28357800	0.49958000
C	1.18009800	-3.00985700	-0.48840200
C	2.57160800	-2.38523500	-0.69485000
C	2.50271700	-0.94563000	-1.12463700
C	3.19773400	0.08966700	-0.63266300
C	2.99120500	1.48644800	-1.17396100
C	2.48454100	2.48073800	-0.10267500
C	1.23548900	1.96116800	0.53930900
C	-0.00382200	2.46993300	0.50766900
C	-1.12138400	1.65548800	1.11871100
C	-1.77628500	0.82458900	-0.00042400
C	-2.79163100	-0.24644600	0.66257200
C	-3.69446600	-0.22358900	-0.45283400
C	-3.53781400	-1.13134200	-1.60995600
C	-4.80927200	0.74496000	-0.47486400
C	-0.41168800	3.76833700	-0.13495200
C	4.20667400	-0.01312500	0.48395700
C	0.95503100	-1.89224300	1.79071700
H	-1.87462800	2.29458400	1.59514800
H	-1.02908200	0.22983800	-0.53487700
H	-2.24986900	1.50295300	-0.72115200
H	-2.85831900	-2.29757200	1.27024000
H	-1.66341700	-1.33386000	2.09574200
H	-0.72510400	0.97137600	1.87651700
H	-3.24223300	0.25942600	1.52180000
H	-4.93718300	1.20016100	-1.46103300
H	-5.71641200	0.14205500	-0.30027700
H	-4.74416600	1.49936300	0.30964700
H	-4.29156300	-0.96377400	-2.37838400
H	-2.53757300	-1.00940800	-2.04375800
H	-3.58216300	-2.16937100	-1.25444800
H	-1.23400300	3.62412700	-0.84629200
H	-0.76860500	4.47180400	0.62567700
H	0.40710200	4.24452200	-0.67384400
H	2.32921200	3.45839500	-0.56449400
H	3.25829600	2.61593500	0.66289600
H	3.93920400	1.86633100	-1.57539500
H	2.27309100	1.45864600	-2.00086300
H	1.80608400	-0.74067000	-1.94131800
H	4.49958300	-1.03928000	0.70626900
H	5.11413000	0.54269500	0.22396200
H	3.82115200	0.43526600	1.40928300
H	3.08488700	-2.97625200	-1.46343900
H	3.16156100	-2.49654600	0.21847200
H	0.66385700	-3.08364000	-1.45247900
H	1.31728400	-4.03806100	-0.12712100
H	1.35594000	1.00599600	1.05476700
H	1.77852800	-1.19771700	1.59024200
H	0.28404900	-1.42425700	2.51234300
H	1.39622500	-2.77672600	2.26472800
H	-1.33889500	-2.48868900	-0.75738800

TS_A-B

Sum of electronic and thermal Free Energies = -780.908757

Number of imaginary frequensies = 1

The magnitude of the imaginary frequency: -318.0852

C	-2.11163500	-1.18234000	0.94602300
C	-1.14656000	-1.87111700	0.00910800
C	0.10557300	-2.26257900	0.28590000
C	0.91012600	-2.99078900	-0.76933500
C	2.37039600	-2.51807100	-0.89589200
C	2.48750500	-1.04249000	-1.16558900
C	3.28943600	-0.15933300	-0.55339600
C	3.28864000	1.29785300	-0.95854500
C	2.89866500	2.25181000	0.19490000
C	1.56705200	1.85941700	0.76137000
C	0.37401400	2.41522700	0.51839100
C	-0.86997000	1.72495300	1.03107400
C	-1.64495000	1.10226000	-0.14514100
C	-2.67239400	0.05576100	0.24952000
C	-3.98437500	0.03181600	-0.29350000
C	-4.88741700	-1.15473400	-0.09461200
C	-4.55975700	1.15345400	-1.09496600
C	0.14385900	3.64789600	-0.31714100
C	4.24189100	-0.50188100	0.56496200
C	0.79116700	-2.08961500	1.61305500
H	-1.52397500	2.42806700	1.56617900
H	-0.92792800	0.55662700	-0.77245500
H	-2.07963000	1.88533400	-0.76618500
H	-2.91650200	-1.86008800	1.24202100
H	-1.60840000	-0.87194600	1.86226700
H	-0.57080000	0.94557100	1.73838600
H	-3.51931700	0.56590800	0.98046000
H	-4.55609900	0.80888000	-2.13877200
H	-5.60384400	1.32073800	-0.82183100
H	-4.00497700	2.08644100	-1.03975600
H	-5.85017100	-0.98735100	-0.57575800
H	-4.41600000	-2.04490200	-0.52359800
H	-5.06550200	-1.36227700	0.96399300
H	-0.28704500	3.39706700	-1.29462200
H	-0.55726400	4.32583100	0.18285200
H	1.06431400	4.20115500	-0.50350200
H	2.89296500	3.27731200	-0.18243300
H	3.66165100	2.21125500	0.98031900
H	4.28915900	1.57497200	-1.31503400
H	2.59097300	1.44741200	-1.79026700
H	1.84917000	-0.66840000	-1.96956300

H	4.37731800	-1.57456700	0.70412500
H	5.22705500	-0.06531700	0.36737100
H	3.90008200	-0.07875900	1.51873200
H	2.82456500	-3.08018200	-1.72122400
H	2.92344000	-2.79975000	0.00375400
H	0.40611400	-2.89732900	-1.73780500
H	0.91674100	-4.05980900	-0.51787900
H	1.57634400	0.95691400	1.37568100
H	1.66850500	-1.44358300	1.49538700
H	0.15341200	-1.66633100	2.39044600
H	1.15451700	-3.05922800	1.97177000
H	-1.53646100	-2.07466300	-0.99017700

B

Sum of electronic and thermal Free Energies = -780.92256

Number of imaginary frequensies = 0

C	-2.00916200	-1.72322300	0.43149200
C	-0.78101300	-2.14918800	-0.32197600
C	0.45420700	-2.32998700	0.16824800
C	1.52795200	-2.91636500	-0.72257900
C	2.89807400	-2.22310900	-0.63016100
C	2.82434300	-0.75843700	-0.96163300
C	3.38465900	0.26248600	-0.29821700
C	3.19991300	1.68361800	-0.77986400
C	2.47342000	2.57956200	0.25113900
C	1.17905200	1.94534300	0.66200000
C	-0.04818500	2.21957200	0.19998400
C	-1.20187400	1.38723700	0.69252100
C	-1.83902200	0.60734000	-0.55604300
C	-2.63984800	-0.43989300	0.04224000
C	-4.08005500	-0.27390400	0.30241300
C	-4.69958700	-0.73660300	-1.06292000
C	-4.57212800	1.12616500	0.66870400
C	-0.38863400	3.28011000	-0.81121300
C	4.21517600	0.11769000	0.95212200
C	0.87137100	-2.05300600	1.58545200
H	-1.98921000	2.00386000	1.13573100
H	-1.00502300	0.20083900	-1.13169100
H	-2.42589100	1.32466300	-1.13277900
H	-2.78619300	-2.50108600	0.43143500
H	-1.80378200	-1.57058200	1.50860000
H	-0.84759000	0.67121600	1.43974700
H	-4.39355900	-1.00151500	1.05966400
H	-4.29107900	1.87278300	-0.07935900
H	-5.66284000	1.11024400	0.72302900
H	-4.19504000	1.44082000	1.64395400
H	-5.78229500	-0.76702200	-0.92039400
H	-4.47098100	-0.02025400	-1.85537700
H	-4.36721800	-1.73160500	-1.36780400
H	-0.63072300	2.83790600	-1.78584100
H	-1.26069400	3.86093800	-0.48950500
H	0.43557300	3.97521400	-0.97020200
H	2.32063500	3.57087000	-0.18215600
H	3.10754000	2.71637000	1.13365500
H	4.17880200	2.12983100	-0.99608100
H	2.62572300	1.68346500	-1.71295600
H	2.25525300	-0.51976800	-1.86320000
H	4.48672500	-0.91508000	1.17110000
H	5.14324400	0.69186700	0.85666500
H	3.68921600	0.52053100	1.82778200
H	3.56742400	-2.72770000	-1.33769400
H	3.32670200	-2.38530400	0.36233100
H	1.17984600	-2.90956400	-1.76127800
H	1.65473500	-3.97022200	-0.44026800
H	1.27107100	1.12200400	1.37199600
H	1.63363600	-1.26473800	1.59480800
H	0.05333600	-1.74079900	2.23862500
H	1.32965100	-2.94524300	2.02590200
H	-0.95893800	-2.39538500	-1.36869000

TS_B-C

Sum of electronic and thermal Free Energies = -780.910005

Number of imaginary frequensies = 1

The magnitude of the imaginary frequency: -426.0857

C	-1.73924400	-1.48616800	-0.05496700
C	-0.36069800	-1.65157300	-0.52606800
C	0.39457600	-2.73392300	-0.23402900
C	1.73524200	-2.93595900	-0.89950100
C	2.90162200	-2.17580800	-0.22985600
C	2.93472900	-0.72436000	-0.63059500
C	3.40615200	0.31408900	0.07011000
C	3.37416500	1.70320600	-0.52384900
C	2.49553400	2.68290100	0.28586900
C	1.14258500	2.09739000	0.55982300
C	-0.03508000	2.42216300	0.01159800
C	-1.27253500	1.69105800	0.47150600
C	-2.00454700	0.94316200	-0.70157100
C	-2.54747900	-0.31822600	-0.12641300
C	-3.96349200	-0.37606300	0.39571300
C	-4.98379900	-0.22783700	-0.74530800
C	-4.18380100	0.70846700	1.46339400
C	-0.26296400	3.49875600	-1.01465100
C	3.98394100	0.20966900	1.45692800
C	0.01765700	-3.81588500	0.73724800
H	-1.98331500	2.39310100	0.91977100
H	-1.27816800	0.72985800	-1.48966200
H	-2.79808400	1.56250300	-1.12427400
H	-2.47948800	-1.47245300	-1.02881100
H	-2.15982900	-2.29206900	0.54472900
H	-0.99058100	0.97463200	1.25153900

H	-4.10709000	-1.35630300	0.86478600
H	-4.16055400	1.70861100	1.02190200
H	-5.17394500	0.56199400	1.90049100
H	-3.44605100	0.65824700	2.26799400
H	-5.99199500	-0.30972700	-0.33262900
H	-4.90378500	0.74673700	-1.23427800
H	-4.86788900	-1.00949900	-1.50234400
H	-0.73234200	3.09240200	-1.91898600
H	-0.93859000	4.26799400	-0.62266600
H	0.66279000	3.98586900	-1.32053300
H	2.42781700	3.63136200	-0.25222700
H	2.98209700	2.90389100	1.24329100
H	4.39270300	2.10804500	-0.57458600
H	2.99099500	1.65100400	-1.54873900
H	2.57953500	-0.51992500	-1.64298900
H	4.10866600	-0.82142800	1.78925700
H	4.96663200	0.69289100	1.49063400
H	3.35460300	0.72880000	2.19026200
H	3.83082100	-2.66361800	-0.54980600
H	2.84717300	-2.29336700	0.85786400
H	1.67613000	-2.63058100	-1.94967100
H	1.95689500	-4.00761000	-0.89008100
H	1.13980900	1.28117700	1.28439300
H	0.86231700	-3.99403900	1.41178800
H	-0.85518800	-3.60230300	1.35400700
H	-0.15074200	-4.75481500	0.19834200
H	0.04327000	-0.90171800	-1.20105200

C

Sum of electronic and thermal Free Energies = -780.92875

Number of imaginary frequensies = 0

C	-1.61801400	-1.51033000	-0.23449300
C	-0.26961800	-1.51953700	-0.50850600
C	0.49689900	-2.67967700	-0.30706100
C	1.77450300	-2.85362800	-1.03887400
C	2.95631200	-2.08488600	-0.35090000
C	2.94963900	-0.61589500	-0.65976100
C	3.29835300	0.39231900	0.15166600
C	3.26325000	1.81423200	-0.35773400
C	2.31128200	2.72150500	0.45377400
C	0.95709600	2.09424200	0.60833900
C	-0.19627500	2.42566900	0.01366100
C	-1.43093400	1.60692200	0.30957800
C	-1.99815800	0.89609300	-0.93854700
C	-2.60471600	-0.48389600	-0.64884300
C	-3.86761700	-0.54063800	0.26047000
C	-4.93744300	0.43703600	-0.22233000
C	-3.58338500	-0.34093600	1.75377100
C	-0.38923600	3.57178000	-0.94220700
C	3.74759300	0.22642700	1.57893400
C	0.11714600	-3.77788600	0.61709100
H	-2.20791900	2.25076900	0.74262500
H	-1.20304200	0.77788200	-1.68503600
H	-2.76058900	1.51122000	-1.42439600
H	-2.95106500	-0.88821600	-1.62344800
H	-2.04862300	-2.39580600	0.23649300
H	-1.17643300	0.86616900	1.07673700
H	-4.26534500	-1.55829400	0.13703100
H	-3.33675300	0.69725100	1.98649800
H	-4.47254000	-0.60384800	2.33289900
H	-2.76009200	-0.97058800	2.11421900
H	-5.86727600	0.27672500	0.32928600
H	-4.63050000	1.47413700	-0.05172000
H	-5.15255700	0.31298500	-1.28835300
H	-0.78629500	3.22008700	-1.90156800
H	-1.11992200	4.28215200	-0.53840000
H	0.53396100	4.11479600	-1.14539000
H	2.25423600	3.69616000	-0.03634400
H	2.73789100	2.90168000	1.44776800
H	4.27471800	2.23801300	-0.31869700
H	2.94676200	1.81685200	-1.40616900
H	2.68946600	-0.36453900	-1.68974000
H	3.88058300	-0.81434600	1.87563500
H	4.70477600	0.73824100	1.72632200
H	3.03547000	0.69072400	2.27074800
H	3.86124600	-2.55254900	-0.75616700
H	2.95370000	-2.28416100	0.72462000
H	1.67902600	-2.47110900	-2.06025700
H	2.02583000	-3.91631900	-1.07853600
H	0.92895800	1.23511900	1.28248800
H	0.99835100	-4.03341900	1.21876700
H	-0.71203200	-3.55481000	1.28481900
H	-0.10430900	-4.67807800	0.02867500
H	0.19026000	-0.68836900	-1.03957000

E

Sum of electronic and thermal Free Energies = -780.904583

Number of imaginary frequensies = 0

C	-1.11476600	-0.95706200	-0.01716500
C	-0.27605200	-1.80556900	-0.62292600
C	0.99739800	-2.28851800	-0.05165500
C	2.09007300	-2.64926200	-1.04400400
C	3.43344800	-1.95708000	-0.78776300
C	3.34529000	-0.43483600	-0.95512100
C	3.02325400	0.47192800	0.15094300
C	2.63118700	1.85859100	-0.17342900
C	1.77635200	2.63726200	0.85051800
C	0.35633200	2.13874800	0.90399600
C	-0.62249300	2.51663600	0.07008200
C	-2.01867300	1.95303000	0.18676900

C	-2.37079800	0.98277700	-0.96827300
C	-2.44302200	-0.50179900	-0.55258000
C	-3.56376300	-0.81837600	0.46862600
C	-3.67585900	-2.33033800	0.68353600
C	-4.91413300	-0.24335400	0.03777200
C	-0.44487700	3.53244600	-1.02899700
C	3.26581200	0.12663700	1.55348100
C	1.17476300	-2.46964300	1.26626100
H	-2.72433300	2.79073600	0.19038500
H	-1.61587300	1.06269500	-1.75967900
H	-3.32039000	1.26729900	-1.42791700
H	-2.66253400	-1.07851600	-1.46361300
H	-0.82905500	-0.55281500	0.95587000
H	-2.13343100	1.45537400	1.15439900
H	-3.28992200	-0.36116600	1.43105400
H	-5.18824000	-0.60215300	-0.96135700
H	-5.69930200	-0.55813600	0.73027900
H	-4.91430700	0.84975500	0.01826700
H	-4.45214200	-2.55916400	1.41896800
H	-3.94676100	-2.83079200	-0.25368600
H	-2.73659800	-2.76624500	1.03524800
H	-0.84434700	3.15057000	-1.97365700
H	-1.01872800	4.43543400	-0.78998300
H	0.59235100	3.82889200	-1.19894800
H	1.81029000	3.68698600	0.55054800
H	2.23519700	2.59613200	1.84210600
H	3.62455300	2.34865200	-0.24105900
H	2.19232200	1.91209600	-1.17416400
H	2.78509800	-0.15418400	-1.85351900
H	3.72945000	0.95820500	2.09376500
H	2.25552900	0.00789000	1.98388400
H	3.80748800	-0.80703100	1.69746000
H	4.16065200	-2.32174900	-1.51948400
H	3.83104500	-2.22597700	0.19508600
H	1.75950800	-2.40873500	-2.06074300
H	2.25795000	-3.73134200	-1.02341400
H	0.11869600	1.42162900	1.69020000
H	0.36956100	-2.30655200	1.97590600
H	2.11196900	-2.84273200	1.66748400
H	-0.53838300	-2.19243500	-1.60754100
H	4.37584600	-0.04592800	-1.14257400

TS_E-F

Sum of electronic and thermal Free Energies = -780.884213

Number of imaginary frequensies = 1

The magnitude of the imaginary frequency: -212.7532

C	-0.30964500	-0.75885800	0.15337100
C	0.93689800	-0.87630400	-0.41605400
C	2.07405600	-1.62497400	0.19617500
C	3.30914400	-1.66288200	-0.66630500
C	3.99651700	-0.29556800	-0.74372700
C	3.03900700	0.85302500	-1.05989700
C	1.97306300	1.19388800	-0.05129600
C	1.07999400	2.33595900	-0.46261500
C	0.22956400	3.02770700	0.61992500
C	-0.93947400	2.16012500	0.97397300
C	-2.12494100	2.17714900	0.35115300
C	-3.00772600	0.95727600	0.39921700
C	-2.60341600	0.10318500	-0.82548800
C	-1.60493300	-1.04309900	-0.56480800
C	-2.23930200	-2.23522100	0.23330600
C	-1.33550500	-3.47102300	0.15632400
C	-3.63943200	-2.59252700	-0.27006500
C	-2.60299800	3.25751200	-0.58205100
C	2.31265000	1.09644300	1.39260800
C	2.00006100	-2.26182800	1.37133800
H	-4.06560300	1.23022700	0.33314800
H	-2.16062300	0.75837900	-1.58387500
H	-3.47705500	-0.34538500	-1.30248800
H	-1.33707800	-1.43208100	-1.55716400
H	-0.36765100	-0.72216500	1.24418800
H	-2.87249100	0.39884900	1.33240100
H	-2.32663000	-1.92703700	1.28525800
H	-3.63554600	-2.77439500	-1.35101200
H	-3.97496600	-3.51144400	0.21807700
H	-4.37752700	-1.81774400	-0.05047100
H	-1.76676100	-4.29659700	0.72784300
H	-1.23725200	-3.80153100	-0.88463600
H	-0.32660900	-3.29285600	0.53992700
H	-2.90671900	2.84367700	-1.54966500
H	-3.49530100	3.72509900	-0.15089700
H	-1.87093000	4.04404400	-0.76377300
H	-0.09962800	3.98389900	0.20917700
H	0.84396600	3.27113500	1.49155100
H	1.79309300	3.07084300	-0.86985800
H	0.45797800	2.04063800	-1.31657900
H	2.58947300	0.75584100	-2.05537700
H	2.88701900	2.01239600	1.61027900
H	1.42934900	1.12160700	2.03341900
H	2.93288500	0.24218500	1.64548800
H	4.76508600	-0.31581700	-1.52110900
H	4.51890500	-0.08878700	0.19610500
H	3.02587500	-1.98408400	-1.67716400
H	4.01231900	-2.40512700	-0.28113400
H	-0.76095600	1.36273200	1.69438000
H	1.10665700	-2.26367700	1.98833800
H	2.85186300	-2.81950200	1.74808700
H	0.95182500	-0.88553600	-1.50819300
H	3.63154900	1.78232300	-1.10477500

F

Sum of electronic and thermal Free Energies = -780.96274

Number of imaginary frequensies = 0

C	-0.46448000	-0.12886000	0.43533800
C	0.90173600	-0.24770600	-0.26739900
C	1.31163500	-1.65853900	-0.65606100
C	2.49937600	-1.67571900	-1.58686200
C	3.70319000	-1.01458100	-0.89636100
C	3.33609000	0.37056600	-0.36048400
C	2.08528700	0.38443300	0.54358600
C	1.75204800	1.84709800	0.87322400
C	0.41304900	2.01735800	1.59141600
C	-0.74574900	1.40723600	0.80172900
C	-1.21859900	2.14282100	-0.37383500
C	-2.35523100	1.59292800	-1.11949000
C	-1.96403300	0.14385900	-1.56178700
C	-1.65883900	-0.71934700	-0.34202300
C	-2.88782300	-0.99081400	0.56213900
C	-2.61376600	-2.14460800	1.53464700
C	-4.14609900	-1.31509400	-0.25021700
C	-0.58874500	3.36481600	-0.89151900
C	2.36849600	-0.38829300	1.84081800
C	0.81623600	-2.77831500	-0.12308700
H	-2.63200900	2.22113500	-1.96765400
H	-1.10012900	0.19094400	-2.23256400
H	-2.79340700	-0.25139300	-2.15080000
H	-1.33983900	-1.69191300	-0.73557400
H	-0.39380900	-0.62000700	1.41094400
H	-3.21850100	1.49700700	-0.44570100
H	-3.10254700	-0.09261900	1.16531100
H	-3.95533500	-2.14552300	-0.94003400
H	-4.95667900	-1.61945900	0.41631000
H	-4.51341300	-0.46607900	-0.83480800
H	-3.46808100	-2.29099000	2.20015800
H	-2.46224000	-3.07760100	0.98013100
H	-1.73741000	-1.97940300	2.16774700
H	-0.01992200	3.04280800	-1.78401900
H	-1.34622200	4.05917100	-1.26733800
H	0.10185100	3.84967300	-0.20569700
H	0.21478000	3.07197800	1.81080500
H	0.44713300	1.51675900	2.56370400
H	2.55263900	2.27061600	1.49002300
H	1.75606500	2.42062900	-0.06376800
H	3.15211500	1.04024500	-1.21406300
H	3.19817700	0.08648500	2.37446300
H	1.51090400	-0.40767200	2.52034300
H	2.64164100	-1.42614400	1.64524400
H	4.53693400	-0.92712100	-1.59926000
H	4.04699000	-1.65971100	-0.08046900
H	2.25958300	-1.11749000	-2.50318900
H	2.73807900	-2.70110300	-1.88111300
H	-1.65067200	1.34320100	1.43377900
H	0.01758300	-2.78210200	0.61329200
H	1.22167800	-3.74700700	-0.39962300
H	0.85157100	0.33909000	-1.20386100
H	4.17981300	0.79815200	0.19465000

E2

Sum of electronic and thermal Free Energies = -780.958841

Number of imaginary frequensies = 0

C	-1.20965300	-0.74230200	-0.09856500
C	-0.23012100	-1.33424100	-0.86482300
C	1.08763600	-1.46609800	-0.41376600
C	2.06514700	-2.25996700	-1.21495500
C	3.50852600	-2.08187300	-0.74521200
C	3.78134200	-0.58757200	-0.56582100
C	2.86347900	0.06826800	0.50247700
C	2.39325400	1.43005500	-0.05309400
C	1.53616000	2.33946700	0.84868200
C	0.04753000	2.06689000	0.85925900
C	-0.77884200	2.21578000	-0.19079300
C	-2.27147200	1.99682100	-0.06885000
C	-2.80544800	0.90320100	-1.00660000
C	-2.62423400	-0.56025600	-0.48463500
C	-3.54187100	-0.92281000	0.71111500
C	-3.31430600	-2.38584900	1.10518000
C	-5.01751400	-0.68037600	0.39493800
C	-0.29871300	2.66349700	-1.55046200
C	3.63425600	0.23296900	1.81723700
C	1.63516200	-0.86172300	0.81674500
H	-2.77240800	2.93666900	-0.33228900
H	-2.31616000	0.98305400	-1.98295600
H	-3.87445500	1.03544400	-1.18927100
H	-2.86068600	-1.22707500	-1.32569200
H	-0.94899800	-0.39513900	0.89787000
H	-2.54498200	1.78498100	0.97130700
H	-3.25782600	-0.28716900	1.56201600
H	-5.30676600	-1.18773800	-0.53269600
H	-5.63940200	-1.08242500	1.19894100
H	-5.25884100	0.38053800	0.29782200
H	-3.93194100	-2.65690100	1.96462200
H	-3.58135900	-3.05250600	0.27705700
H	-2.27014900	-2.58733200	1.37373100
H	-0.12042600	1.81249100	-2.22061300
H	-1.04938500	3.29683700	-2.03330800
H	0.63307900	3.23025900	-1.49003000
H	1.68726500	3.37074500	0.50616100
H	1.91909200	2.32010900	1.87322800
H	3.30726700	1.97535300	-0.32167300
H	1.86295200	1.25287000	-0.99828600
H	3.62340200	-0.09845300	-1.53590100
H	4.41220100	0.99473700	1.70524100
H	2.98332500	0.53060100	2.64471300
H	4.12106400	-0.70707200	2.09825600
H	4.18757900	-2.51525900	-1.48274400

H	3.68023800	-2.62436700	0.19186500
H	1.95247100	-1.98616400	-2.27347400
H	1.73871200	-3.31094100	-1.15674900
H	-0.39706300	1.78942400	1.81729500
H	0.90697000	-0.31658600	1.41605900
H	2.01774200	-1.69498300	1.42539400
H	-0.49272100	-1.75228700	-1.83496500
H	4.82830100	-0.41499400	-0.29926400

C2

Sum of electronic and thermal Free Energies = -780.937714

Number of imaginary frequensies = 0

C	-1.11789800	-0.84000800	0.41528700
C	-0.22380200	-1.61206200	-0.29224800
C	0.84873400	-2.28388200	0.29856700
C	1.81994400	-2.98637400	-0.57432400
C	3.12187900	-2.11922200	-0.66481000
C	2.79346400	-0.71684400	-1.08945400
C	3.08568500	0.42883400	-0.45802400
C	2.63665100	1.74469800	-1.05243300
C	1.94864900	2.69579900	-0.05494600
C	0.74734700	2.12536900	0.65181700
C	-0.54621700	2.39891500	0.41295600
C	-1.62103800	1.89026400	1.34693400
C	-2.73195700	1.06437800	0.67595800
C	-2.21208600	-0.07442400	-0.21235500
C	-3.33486000	-1.10271300	-0.63696000
C	-4.48377700	-0.36302600	-1.32099300
C	-3.84160500	-1.95843700	0.52615200
C	-1.03861600	3.26961900	-0.71226400
C	3.90777000	0.53205200	0.80111700
C	1.18868900	-2.19472300	1.74227800
H	-2.09662300	2.75176800	1.83301400
H	-3.35759600	1.71593500	0.05906400
H	-3.38608100	0.66156800	1.45750900
H	-1.82168400	0.33422100	-1.15573100
H	-1.05822200	-0.83385500	1.50386900
H	-1.15363500	1.31389000	2.15460400
H	-2.86801600	-1.76903100	-1.37336300
H	-4.29259900	-1.34745100	1.31360100
H	-4.61352200	-2.64420200	0.16756200
H	-3.04862000	-2.56756700	0.97410400
H	-5.16901900	-1.08846600	-1.76804900
H	-5.05637500	0.23310700	-0.60464500
H	-4.12630300	0.29885600	-2.11592800
H	-1.72465800	2.71316300	-1.36309300
H	-1.60507900	4.12162600	-0.31886400
H	-0.23368100	3.65242000	-1.33947800
H	1.67867800	3.60955500	-0.58991700
H	2.67523900	3.00316500	0.70665700
H	3.51657400	2.26293600	-1.45665000
H	1.96135700	1.55257300	-1.89356800
H	2.22603200	-0.64529500	-2.01948000
H	4.34459400	-0.41708500	1.11455600
H	4.73216100	1.23730500	0.64711400
H	3.31511700	0.92949300	1.63358600
H	3.76702300	-2.61433000	-1.39801500
H	3.64145400	-2.15028400	0.29528100
H	1.40868500	-3.12868600	-1.57661200
H	2.07605000	-3.96190900	-0.14785700
H	0.96535300	1.47822900	1.50616400
H	1.85938500	-1.32650300	1.84872600
H	0.33299100	-2.03555300	2.39744900
H	1.74479900	-3.07484000	2.07034800
H	-0.31550500	-1.65403100	-1.37549000

TS_C2-G

Sum of electronic and thermal Free Energies = -780.904724

Number of imaginary frequensies = 1

The magnitude of the imaginary frequency: -117.4712

C	-0.68082200	-0.26849800	0.36094000
C	0.35352700	-1.35141300	0.18099900
C	1.36490100	-1.61972800	1.01898600
C	2.52468100	-2.46586900	0.54812200
C	3.61597900	-1.56039900	-0.12141300
C	2.89561500	-0.73503000	-1.13889800
C	2.60180900	0.57099300	-1.11106700
C	1.42444300	1.04434200	-1.92614100
C	0.34903900	1.80781900	-1.10852200
C	-0.05721300	1.15080700	0.30381700
C	-1.01617800	2.15656700	0.71820300
C	-2.44109300	1.84402800	0.56686300
C	-2.77460100	0.86217600	-0.57086100
C	-1.87388200	-0.40235400	-0.60351000
C	-2.66711700	-1.70452400	-0.34982400
C	-3.67105400	-1.96592500	-1.47528800
C	-3.36059000	-1.73252400	1.01544200
C	-0.60583000	3.45774100	1.27193800
C	3.33076000	1.60306700	-0.29005700
C	1.56299500	-0.93553100	2.34543400
H	-3.06377500	2.74340800	0.56600100
H	-2.71069800	1.41031600	-1.51531100
H	-3.82600800	0.58851300	-0.45901900
H	-1.45275100	-0.48413800	-1.61617100
H	-1.06875900	-0.34717800	1.38712300
H	-2.64532000	1.33686800	1.53640300
H	-1.93484600	-2.52213000	-0.36413600
H	-4.08290600	-0.91341400	1.12264100
H	-3.92200800	-2.66328900	1.13042000
H	-2.65277900	-1.68301800	1.84865000

H	-4.14475000	-2.94228900	-1.34503900
H	-4.47297500	-1.21925200	-1.48999300
H	-3.18397200	-1.95786400	-2.45543300
H	-1.15214000	3.62474200	2.21028000
H	0.46777300	3.53857300	1.44148700
H	-0.94746600	4.25605700	0.59826700
H	-0.53461100	1.93652800	-1.74104500
H	0.73822600	2.80658200	-0.88726300
H	1.72104000	1.76121400	-2.70490500
H	0.95975000	0.19148100	-2.42885600
H	2.33794400	-1.33894900	-1.85663800
H	4.15809400	1.16989700	0.27363500
H	3.75199900	2.36950500	-0.95164100
H	2.68160300	2.13137200	0.42061900
H	4.37312000	-2.21074400	-0.56985500
H	4.11308300	-0.94580900	0.63442400
H	2.18092500	-3.19349000	-0.19433100
H	2.97592400	-3.02391800	1.37436700
H	0.85736400	1.20402000	0.90485200
H	2.37453700	-0.19658600	2.27825400
H	0.66680900	-0.42433000	2.70707900
H	1.86397700	-1.66020200	3.10787800
H	0.32138300	-1.87694300	-0.77373200

G

Sum of electronic and thermal Free Energies = -780.924393

Number of imaginary frequensies = 0

C	-0.65728700	-0.11226300	0.30823800
C	0.48259800	-0.82151800	-0.28129400
C	1.36170600	-1.60044700	0.38814000
C	2.46531300	-2.29138900	-0.37318700
C	3.72831200	-1.37299200	-0.49707600
C	3.26289000	-0.09427000	-1.11567100
C	2.85434200	0.98587500	-0.44198400
C	1.93216900	1.97514300	-1.11187400
C	0.78919300	2.52256700	-0.23220100
C	-0.25481200	1.67246400	0.53370900
C	-1.60201400	2.12362800	0.46928900
C	-2.61853600	1.51102400	1.33309000
C	-3.13416200	0.32660300	0.42505800
C	-2.00227000	-0.30841400	-0.40390500
C	-2.27455800	-1.80910000	-0.68988900
C	-3.60653000	-1.97506700	-1.42715300
C	-2.22817000	-2.69740000	0.55704900
C	-2.10970000	2.99491800	-0.62142900
C	3.17630100	1.25140500	1.00559000
C	1.40335600	-1.78369600	1.87861500
H	-3.44652400	2.18963200	1.54834400
H	-3.93227600	0.68946100	-0.22585100
H	-3.57714100	-0.40448800	1.10596700
H	-1.94647500	0.18436900	-1.38594300
H	-0.77631300	-0.36942900	1.36088200
H	-2.20288300	1.12675000	2.26580400
H	-1.47527600	-2.14393700	-1.36169800
H	-3.00302300	-2.43303100	1.28453000
H	-2.40626600	-3.73715300	0.27017200
H	-1.25643400	-2.66131500	1.05856500
H	-3.72443000	-3.00758300	-1.76547000
H	-4.45780700	-1.74671300	-0.77675900
H	-3.66458400	-1.32638300	-2.30742200
H	-3.19694600	2.96270200	-0.69654900
H	-1.81600700	4.02930300	-0.38871900
H	-1.65509200	2.75541400	-1.58709200
H	0.25865200	3.23511700	-0.86565800
H	1.24720300	3.13288900	0.55503500
H	2.49040300	2.87051700	-1.41768100
H	1.52650400	1.53554000	-2.02932200
H	2.97895100	-0.17200300	-2.16695800
H	4.00390200	0.64078800	1.36747300
H	3.45852400	2.30048900	1.14964300
H	2.31207100	1.05828600	1.65718900
H	4.46491800	-1.89559100	-1.11317700
H	4.17344200	-1.22345600	0.49007600
H	2.12367300	-2.54050700	-1.38284700
H	2.74622700	-3.22587700	0.12328100
H	0.05754100	1.50781800	1.56795500
H	2.42417000	-1.61224400	2.23925600
H	0.73737100	-1.12225800	2.43632700
H	1.15293300	-2.81922400	2.13726300
H	0.57215800	-0.76030900	-1.365

Table S1. Z-scores and sequence identities of class I TPS (Sat1646) with class IB and IC TPSs.

Protein	Z-Score*	Sequence identity [%]
BalTS (class IB)	18.8	13
AsR6 (class IC)	11.4	10

* Z-score was calculated by Dali server. Protein 3D structures (PDB accession numbers: Sat1646, 7E4O; BalTS, 5YO8; AsR6, 7OC5) registered in PDB were used for the structural comparison.

Table S2. The results of search workflow for non-canonical TPS candidates.

**Genus name**	***Streptomyces***	***Mycobacterium***	***Nocardia***	**Total**
Genomes	395 (191)	393 (64)	31 (13)	819 (268)
Gene clusters	2198 (962)	1344 (218)	158 (81)	3700 (1261)
Hypothetical proteins in the gene clusters	7877 (4455)	6182 (1379)	675 (443)	14734 (6277)
Non-canonical TPS candidates	5	4	1	10

The numbers in parentheses indicate only the number of genomes / gene clusters / proteins which protein 3D structures were available from the Alphafold DB.

Table S3. Non-canonical TPS candidates.

Type	Detected proteins by structural-model-based search: Accession	Species	Z-score to query protein*: Sat1646	BalTS	AsR6	Homologs analyzed their functions: Accession	Species	Sequence identity [%] / E-value
1	MMAR2565**	*Mycobacterium marinum* M	**20.0** (12)	**15.5** (8)	**9.2** (10)	MBB6373681 (PeuTPS)	*Pseudonocardia eucalypti*	27.6 / 7e-26
						WP_218477332 (NioTPS)	*Nocardia iowensis*	26.5 / 3e-24
	MUL3196	*Mycobacterium ulcerans*	**19.4** (12)	**14.9** (7)	**9.5** (10)	WP_166381380 (CmeTPS)	*Catellatospora methionotrophica*	29.3 / 2e-28
						WP_157545681 (HtsTPS)	*Hamadaea tsunoensis*	29.3 / 5e-31
	MULP02290	*Mycobacterium liflandii*	**20.1** (12)	**15.6** (8)	**9.1** (11)	AGP38205 (SceTPS)	*Sorangium cellulosum*	27.7 / 6e-35
						WP_055470764 (SpaTPS)	*Streptomyces pathocidini*	28.1 / 4e-25
						WP_012187888 (HauTPS)	*Herpetosiphon aurantiacus*	23.0 / 7e-16
	MMRN26100	*Mycobacterium marinum* ATCC 927	**16.1** (13)	**12.4** (10)	**8.7** (9)			with MMAR2565
2	DC74_1164	*Streptomyces noursei* NK660	**12.8** (14)	**12.7** (7)	**6.7** (7)	WP_016572488	*Streptomyces noursei* Brown *et al.* 1953	99.3 / 0.0 with DC74_1164
3	NI25_07840	*Streptomyces* sp. CCM_MD2014	**12.5** (17)	**12.0** (10)	**5.6** (4)	WP_159107621	*Streptomyces ardesiacus*	87.3 / 1e-150 with NI25_07840
4	B1K54_05605	*Streptomyces* sp. Fd1-xmd	**9.6** (15)	**12.0** (8)	**4.9** (8)	WP_076043115	*Streptomyces amritsarensis*	98.3 / 0.0 with B1K54_05605
5	KY5_0590c	*Streptomyces formicae*	**7.8** (10)	**5.7** (10)	**5.2** (5)	WP_229882092 (SalTPS)	*Streptomyces alanosinicus*	50.2 / 0.0 with KY5_0590c
	LK07_30515	*Streptomyces pluripotens*	**6.4** (6)	**7.4** (6)	**6.5** (9)			
6	F5544_04325	*Nocardia arthritidis*	**5.0** (12)	**3.9** (7)	**3.8** (12)	WP_068009759 (NpsTPS)	*Nocardia pseudobrasiliensis*	48.8 / 0.0 with F5544_04325

* Sequence identities [%] are shown in parentheses. Z-score was calculated by Dali server.

** Among the proteins detected by the structural-model-based search, only the function of this protein was analyzed.

Table S4. The top ten proteins with the highest structural similarity to PeuTPS.

Protein (PDB accession No.)	Z-score*
α-Bisabolene synthase (3sae)	21.8
2-Methylisoborneol synthase (4la6)	21.3
epi-Isozizaene synthase (3kb9)	20.8
Pimarane-type diterpene synthase Sat1646 (7e4n)	20.7
Cattleyene Synthase (7y88)	19.9
Sesquiterpene synthase (7ofl)	19.2
Cembrene A synthase (7s5l)	19.0
trichobrasilenol synthase (7w5g)	18.3
Germacradien-4-ol synthase (5i1u)	17.4
Aristolochene synthase (2oa6)	17.3

* Z-score was calculated by Dali server.

Table S5. Primers used in this study.

Name	Sequence (5' -> 3')
MMAR2565-fw (NdeI)	GGAGCGCATATGGCCGGGGAAAACGCCGTGGAC
MMAR2565-rv (KpnI)	GGGTACGGTACCCTAGGCCGGTGGGATCATGCG
WP_016572488-fw (NdeI)	GGATCCCATATGGCTACACACGCGATACGCGCG
WP_016572488-rv (EcoRI)	GCGGGTGAATTCTCACGGGCGTTCCGCATGCGGTC
WP_159107621-fw (NdeI)	GAGCGGCATATGAGCGGGTTCCTTCCCCCGGCCTC
WP_159107621-rv (EcoRI)	AGAACTGAATTCTCACCACGGCCGGCGCACTGATG
WP_076043115-fw (NdeI)	AGAACTCATATGTTCGGAATCATCAGGCCATGCCG
WP_076043115-rv (EcoRI)	GTGCGCGAATTCTCAGTCGCAGCAGTCGCAGCAGC
SalTPS-fw (NdeI)	CGAGCCCATATGACGATCCCGGACCAGGATCCCAG
SalTPS-rv (EcoRI)	GCGGTCGAATTCTCACCTTTCGACCTCGTGGGCAC
NpsTPS-fw (NdeI)	GGTGAGCATATGAGCTACGAGCTGCACCGTGACG
NpsTPS-rv (EcoRI)	ACCAGGGAATTCTTACATCGGTACTTTCGTGTGAC
Mvan3536-fw (NcoI)	AAACCCCCATGGATGCATCGGCACCGTCAGC
Mvan3536-rv (EcoRI)	AAACCCGAATCCCTAGGCGGACCGGTTCGCGGC
PeuTPS-D95A-fw	GTTTGAAGTCGTGAGTGCGAATTTGGGCATTGGCC
PeuTPS-D95A-rv	GGCCAATGCCCAAATTCGCACTCACGACTTCAAAC
PeuTPS-N96A-fw	GTTTGAAGTCGTGAGTGATGCGTTGGGCATTGGCC
PeuTPS-N96A-rv	GGCCAATGCCCAACGCATCACTCACGACTTCAAAC
PeuTPS-N289A-fw	GCTGGTGCGCCTGCTGGCGGATGTGGGCACCCCGC
PeuTPS-N289A-rv	GCGGGGTGCCCACATCCGCCAGCAGGCGCACCAGC
PeuTPS-D290A-fw	GGTGCGCCTGCTGAACGCGGTGGGCACCCCGCTGC
PeuTPS-D290A-rv	GCAGCGGGGTGCCCACCGCGTTCAGCAGGCGCACC
PeuTPS-R338A-fw	GCCGGCGCTGAACGCGCTGCGCAAAGATCTGATTC
PeuTPS-R338A-rv	GAATCAGATCTTTGCGCAGCGCGTTCAGCGCCGGC
PeuTPS-K341A-fw	GAACCGCCTGCGCGCGGATCTGATTCATGGCGAAC
PeuTPS-K341A-rv	GTTCGCCATGAATCAGATCCGCGCGCAGGCGGTTC
PeuTPS-D342A-fw	CCGCCTGCGCAAAGCGCTGATTCATGGCGAACTG
PeuTPS-D342A-rv	CAGTTCGCCATGAATCAGCGCTTTGCGCAGGCGG

Sites for restriction enzymes or mutation introduction are shown under lines.

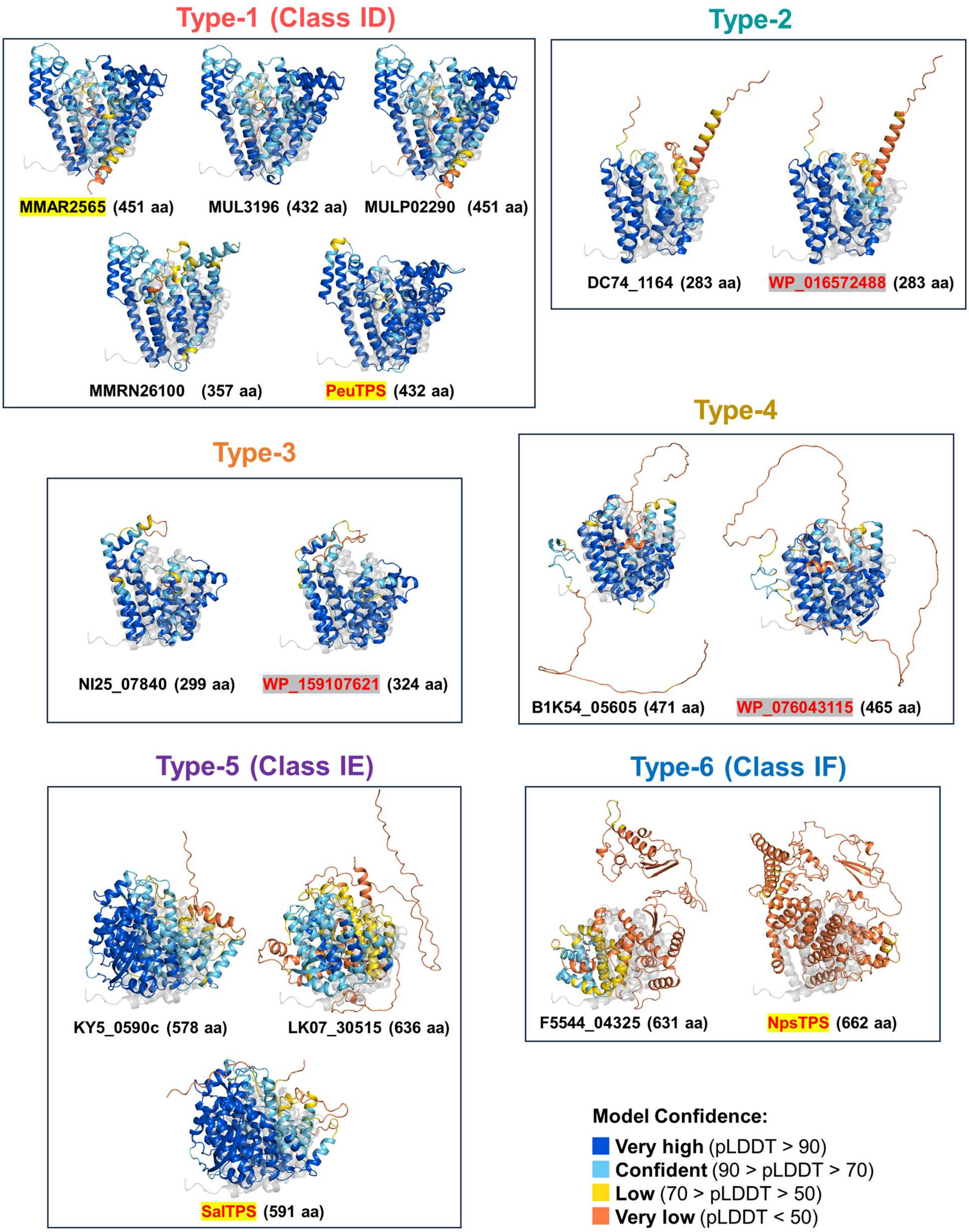

Fig. S1. 3D structural model which is color-coded by the pLDDT values indicating the confidence level of the Alphafold2 prediction of non-canonical TPS candidates. The models of these candidates are shown superposed with class I TPS (Sat1646, gray structure). The names of proteins detected from the structural-model-based search and their homologs are shown in black and red, respectively. Proteins that exhibited and did not exhibit TPS activities are highlighted in yellow and gray, respectively.

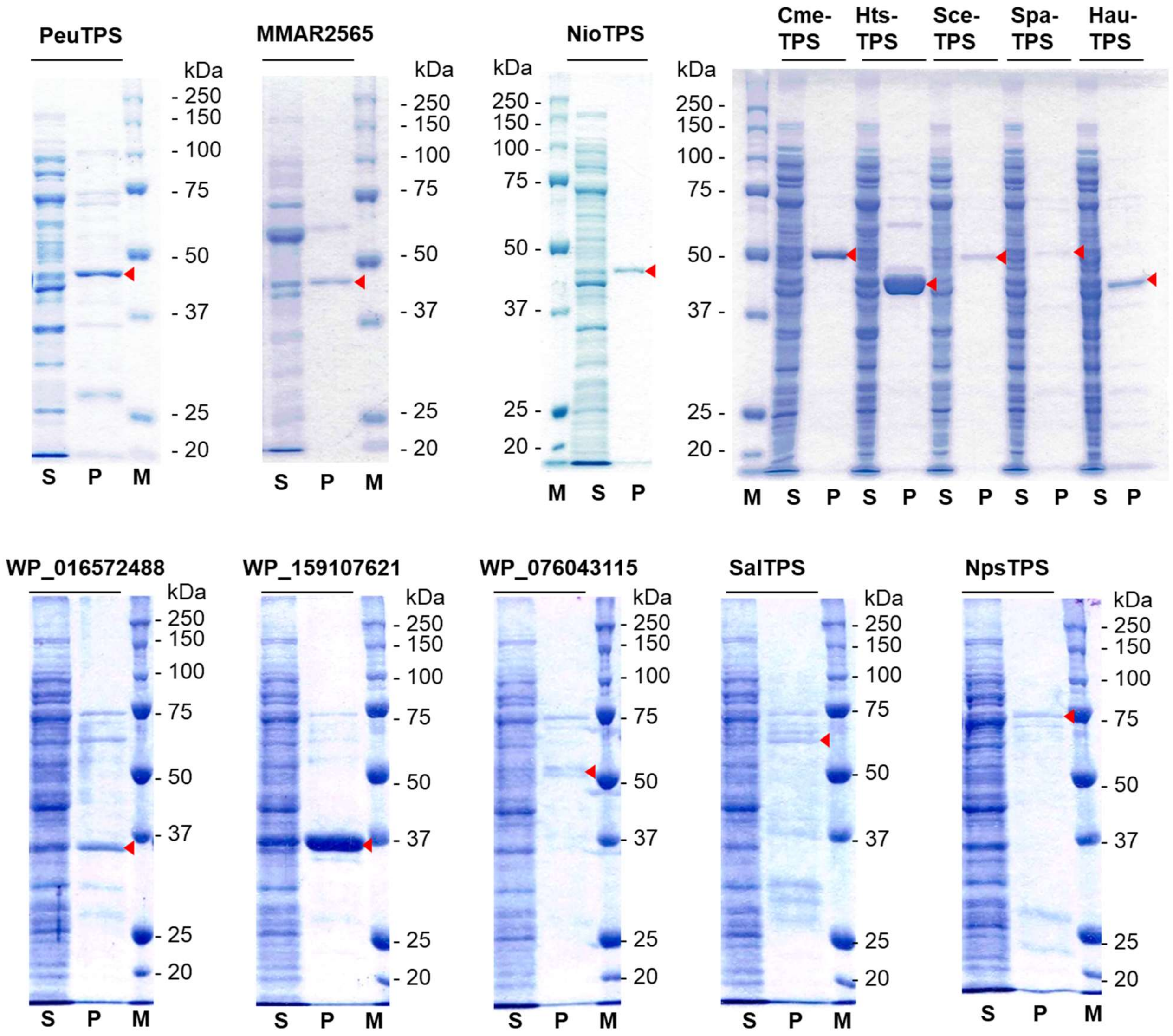

Fig. S2. SDS-PAGE of recombinant proteins. Bands of recombinant TPSs are shown by red triangles. The deduced molecular weights of the TPSs are as follow: PeuTPS, 49.3 kDa; MMAR2565, 51.3 kDa; NioTPS, 50.7 kDa; CmeTPS, 49.8 kDa; HtsTPS, 49.4 kDa; SceTPS, 50.3 kDa; SpaTPS, 51.3 kDa; HauTPS, 51.9 kDa; WP_016572488, 34.4 kDa; WP_159107621, 37.4; WP_076043115, 50.0 kDa; SalTPS, 68.0 kDa; NpsTPS, 74.8 kDa. M: molecular weight marker; P: purified protein; S: total soluble proteins from *E. coli*.

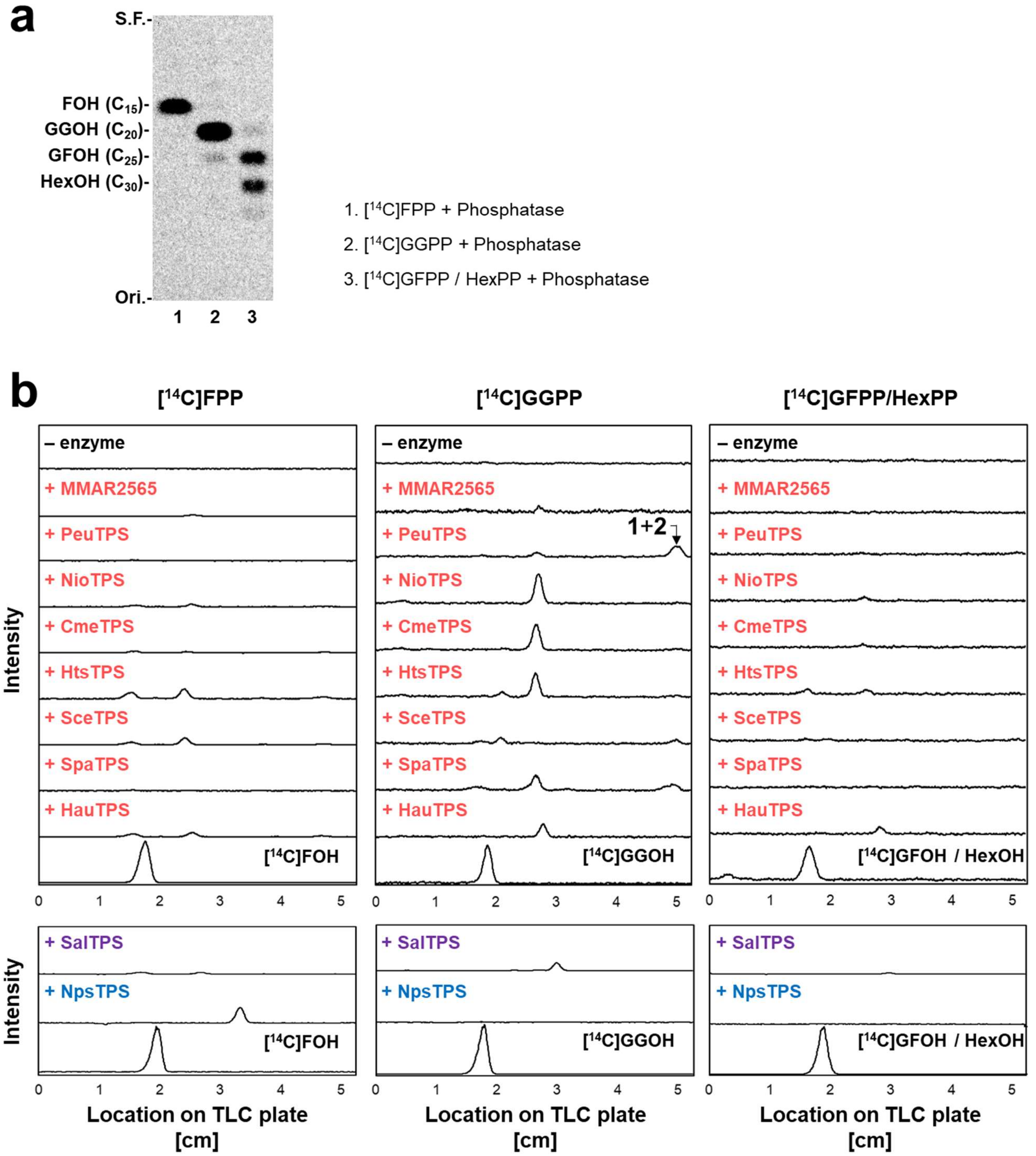

Fig. S3. Enzymatic assay of TPSs using [^{14}C]prenyl diphosphate substrates. a) Reversed-phase TLC analysis of phosphatase-treated ^{14}C-labelled substrates. TLC plate was developed by acetone-water (9:1). Location of co-spotted non-labelled FOH, GGOH, GFOH, and HexOH were shown in the left of the TLC data. b) TLC analysis of the products formed from ^{14}C-labelled substrates by TPSs. The products and 12.5 pmol of [^{14}C]FOH, GGOH, and GFOH/HexOH were developed by *n*-hexane-ethyl acetate (100:20). Intensity of radioactivity toward the location on TLC plate was represented.

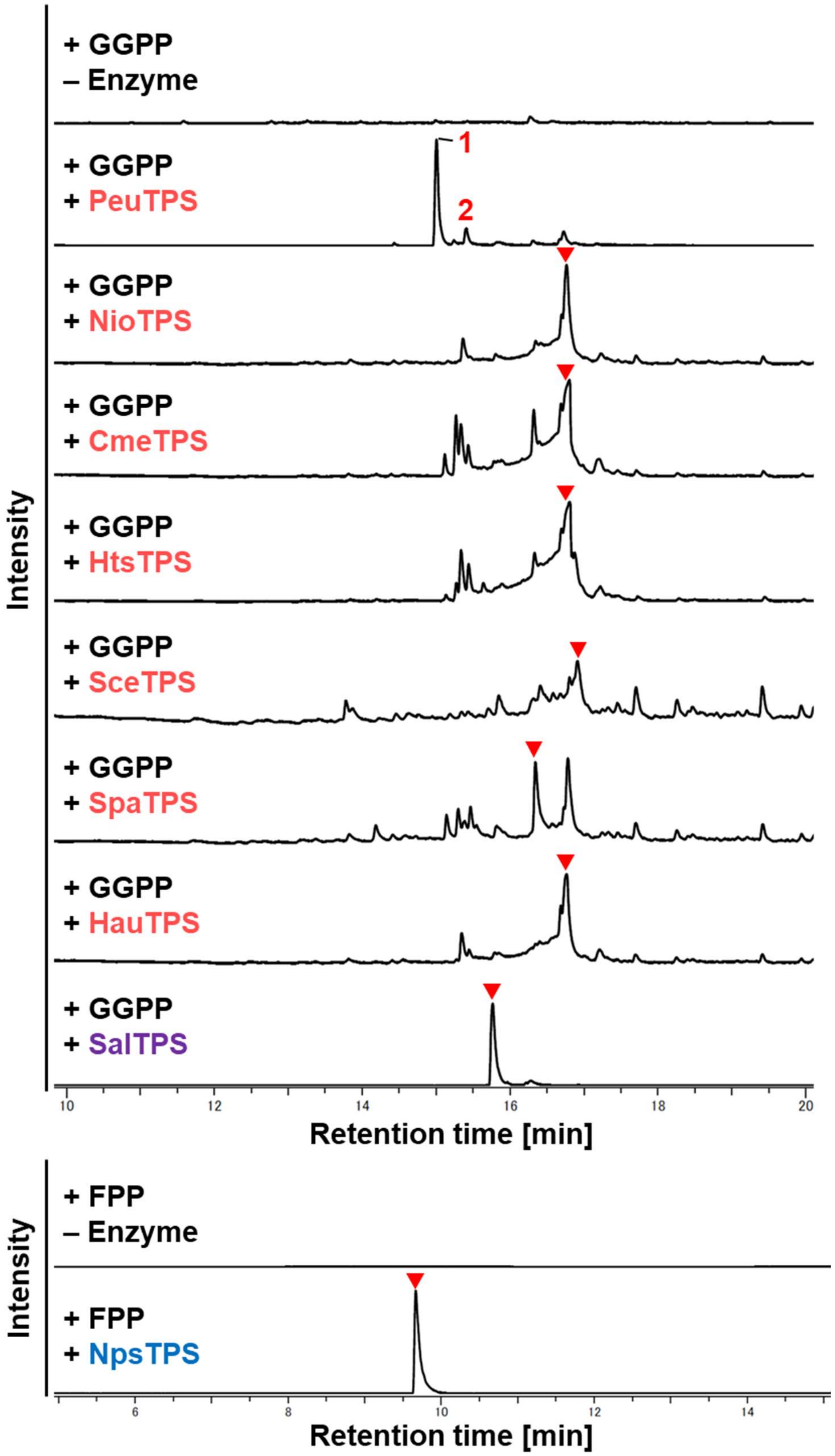

Fig. S4. GC-MS total ion chromatograms of products formed by TPSs. Mass spectra of main products by each TPSs (red triangles) are shown in Fig. S5.

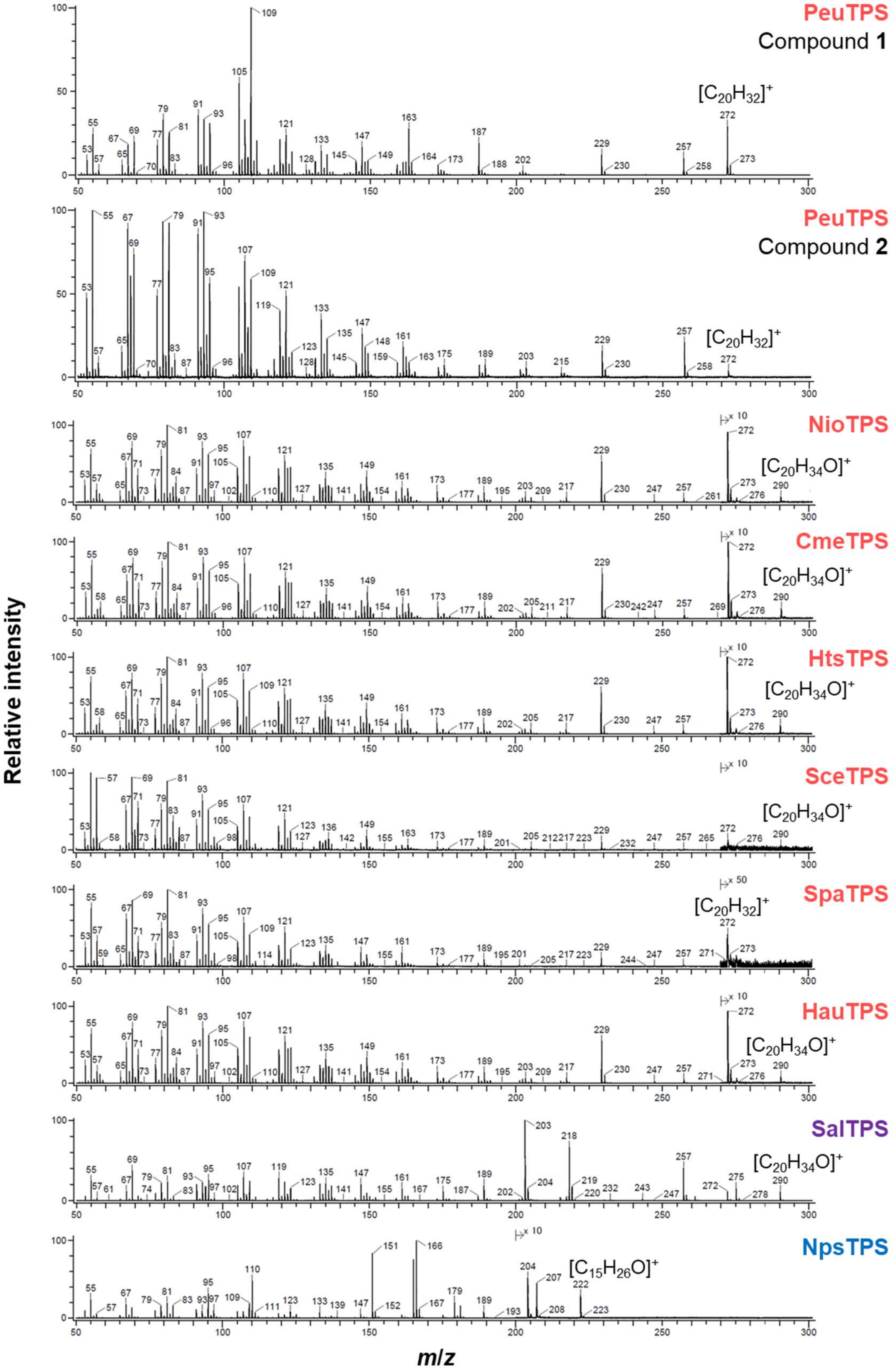

Fig. S5. EI-MS spectra of products formed by TPSs. The characteristic ions are as follows: *m/z* 204, $[C_{15}H_{24}]^+$ corresponding to sesquiterpene hydrocarbon ; *m/z* 222, $[C_{15}H_{26}O]^+$ corresponding sesquiterpene alcohol; *m/z* 272, $[C_{20}H_{32}]^+$ corresponding to diterpene hydrocarbon; *m/z* 290, $[C_{20}H_{34}O]^+$ corresponding diterpene alcohol.

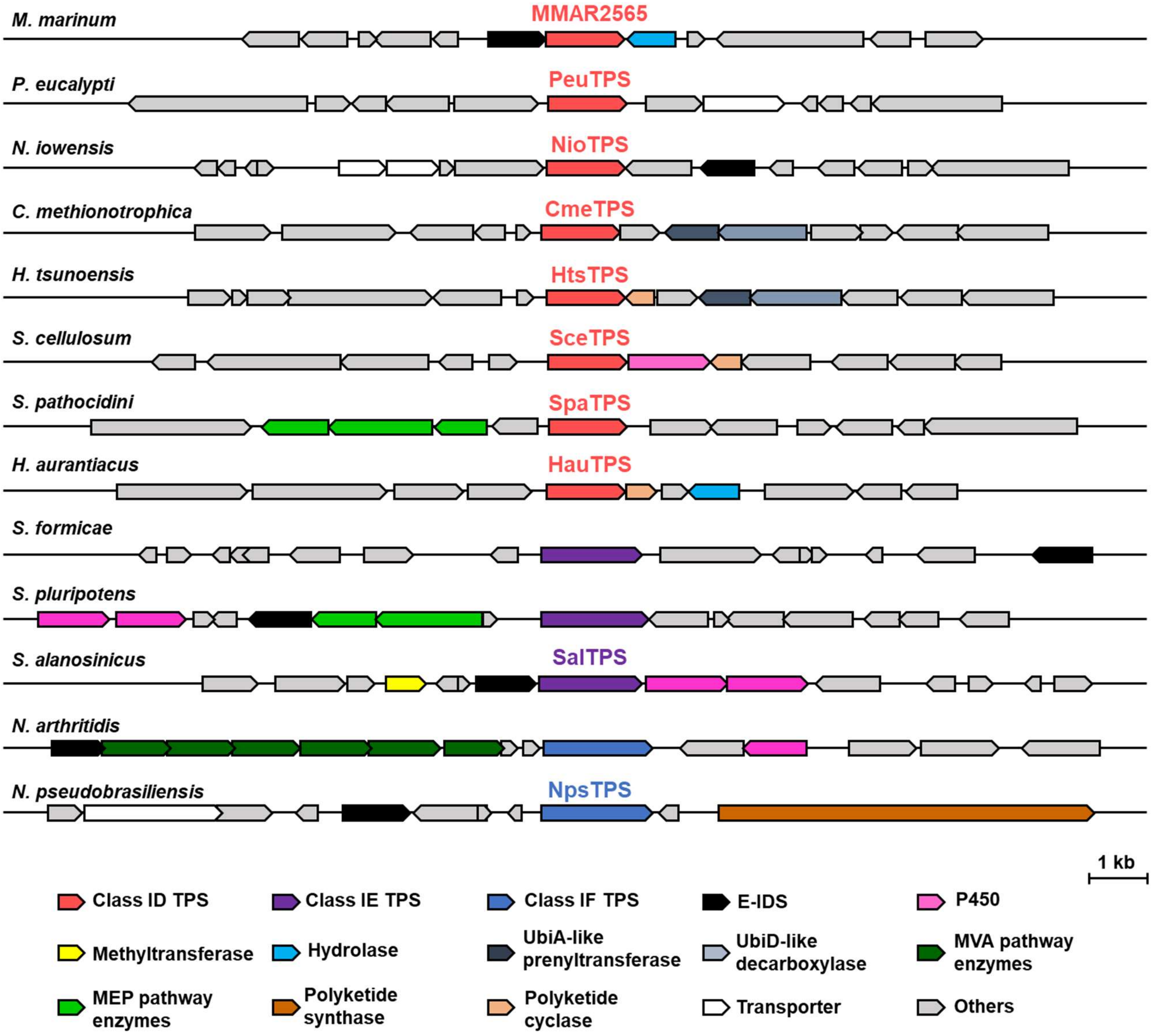

Fig. S6. The flanking region of the genes coding non-canonical TPSs.

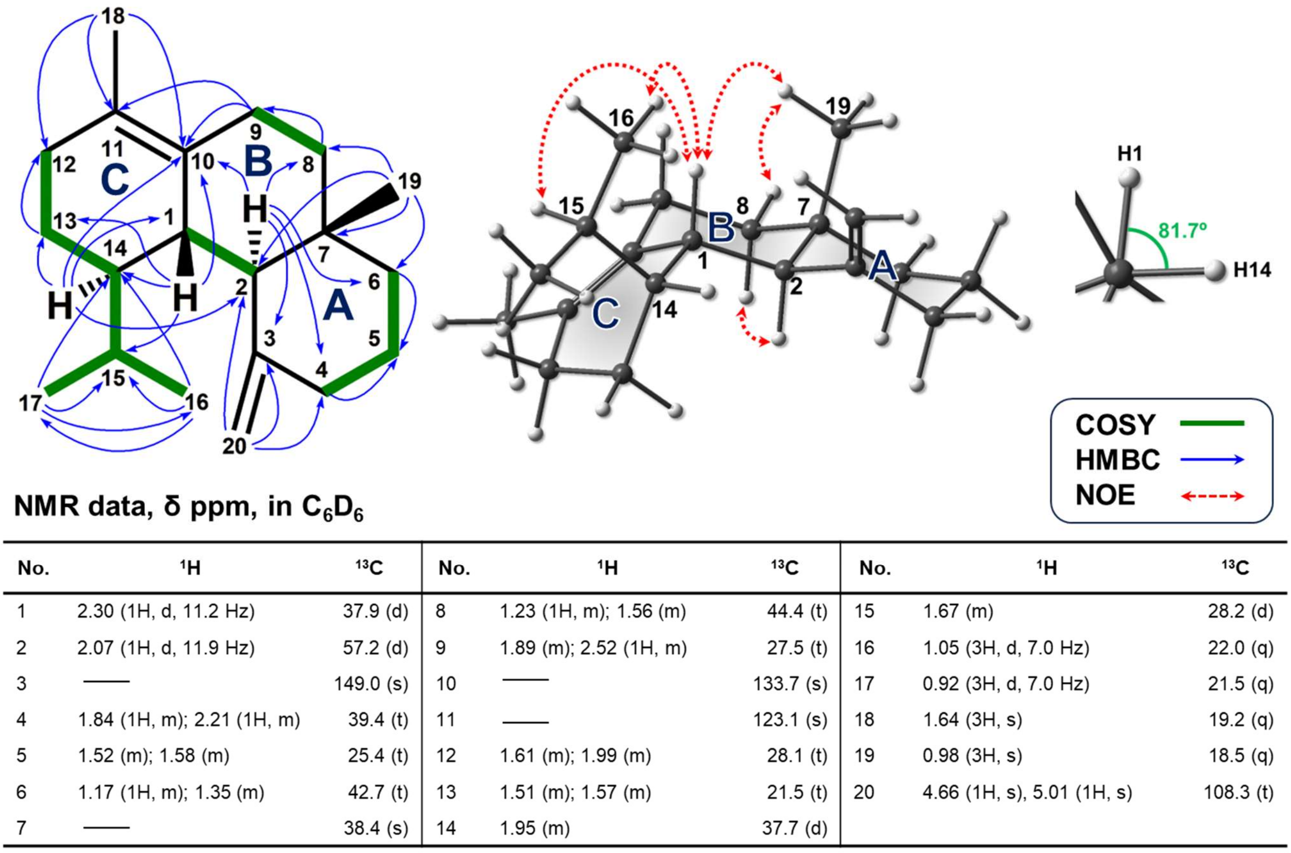

NMR data, δ ppm, in C_6D_6

No.	^{1}H	^{13}C	No.	^{1}H	^{13}C	No.	^{1}H	^{13}C
1	2.30 (1H, d, 11.2 Hz)	37.9 (d)	8	1.23 (1H, m); 1.56 (m)	44.4 (t)	15	1.67 (m)	28.2 (d)
2	2.07 (1H, d, 11.9 Hz)	57.2 (d)	9	1.89 (m); 2.52 (1H, m)	27.5 (t)	16	1.05 (3H, d, 7.0 Hz)	22.0 (q)
3	——	149.0 (s)	10	——	133.7 (s)	17	0.92 (3H, d, 7.0 Hz)	21.5 (q)
4	1.84 (1H, m); 2.21 (1H, m)	39.4 (t)	11	——	123.1 (s)	18	1.64 (3H, s)	19.2 (q)
5	1.52 (m); 1.58 (m)	25.4 (t)	12	1.61 (m); 1.99 (m)	28.1 (t)	19	0.98 (3H, s)	18.5 (q)
6	1.17 (1H, m); 1.35 (m)	42.7 (t)	13	1.51 (m); 1.57 (m)	21.5 (t)	20	4.66 (1H, s), 5.01 (1H, s)	108.3 (t)
7	——	38.4 (s)	14	1.95 (m)	37.7 (d)			

Fig. S7. NMR assignment of **1** measured in C_6D_6. The most stable 3D structures calculated by the conformational search are shown on the center. Since no H1-H14 correlation is observed in ^{1}H,^{1}H-COSY, it is inferred that the H1-H14 dihedral angle is close to 90° (shown in the right), which supports the relative stereochemistry of the 14 position of **1**.

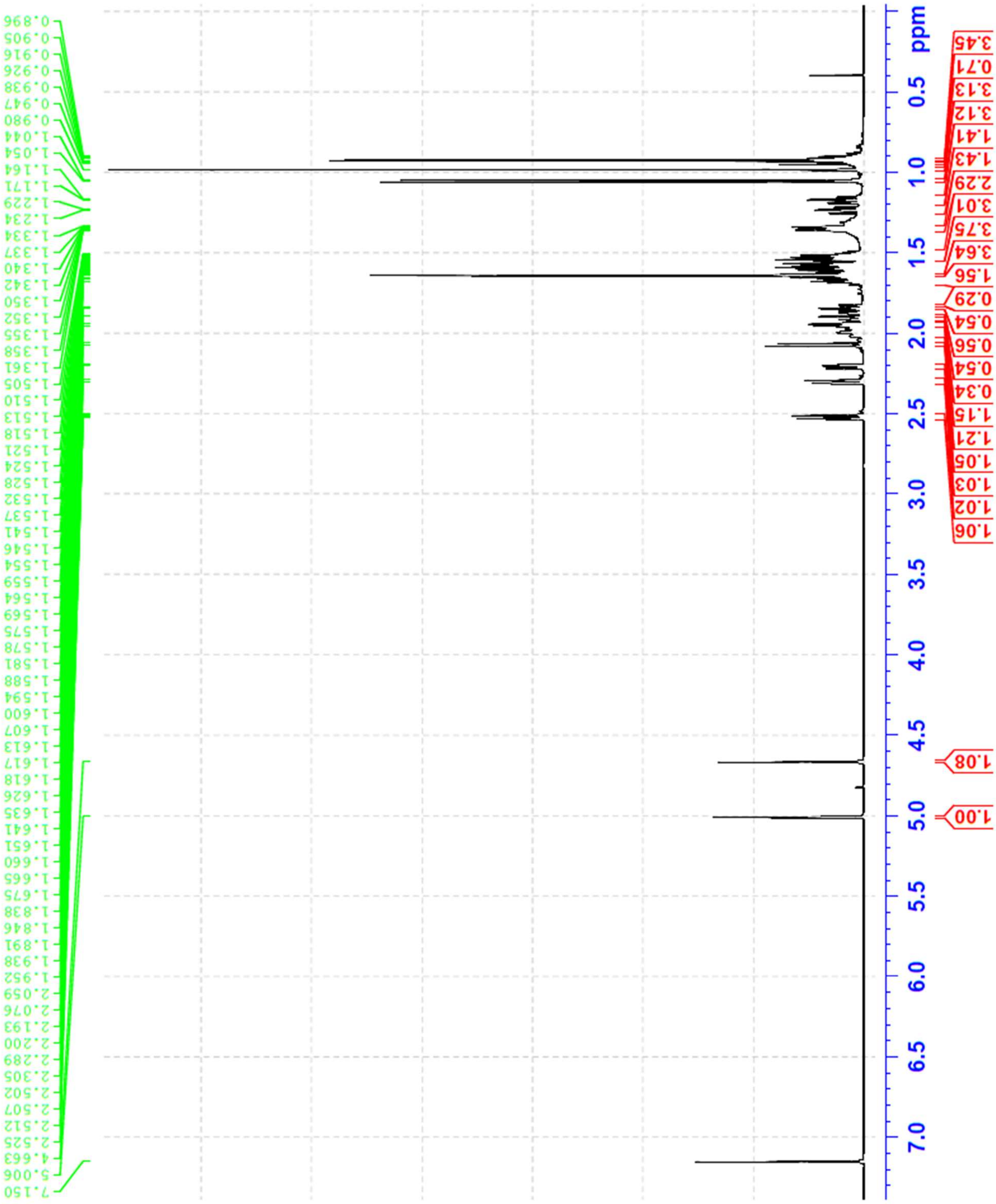

Fig. S8. ^{1}H NMR spectrum of **1** measured in C_6D_6.

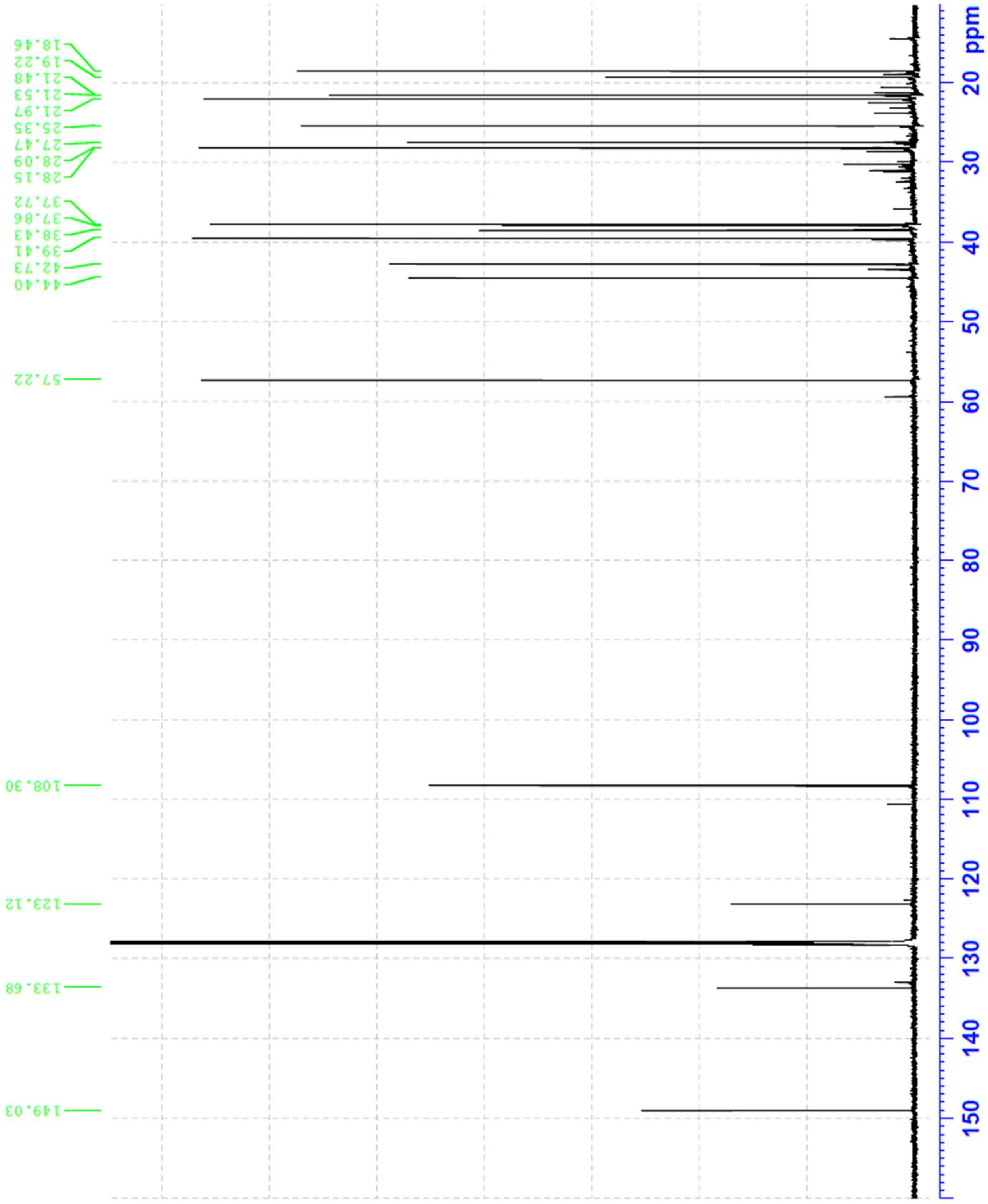

Fig. S9. ^{13}C NMR spectrum of **1** measured in C_6D_6.

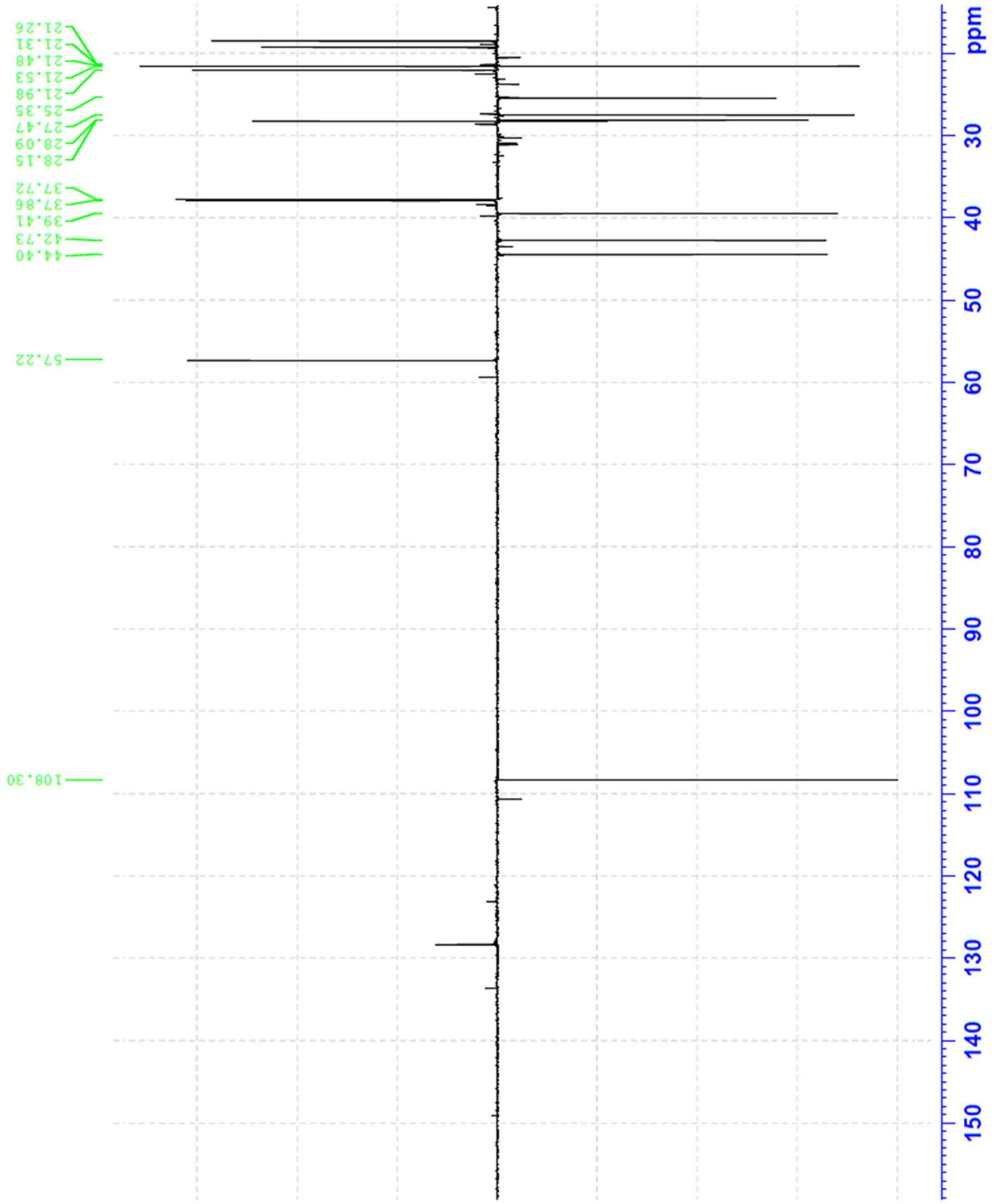

Fig. S10. DEPT135 spectrum of **1** measured in C_6D_6.

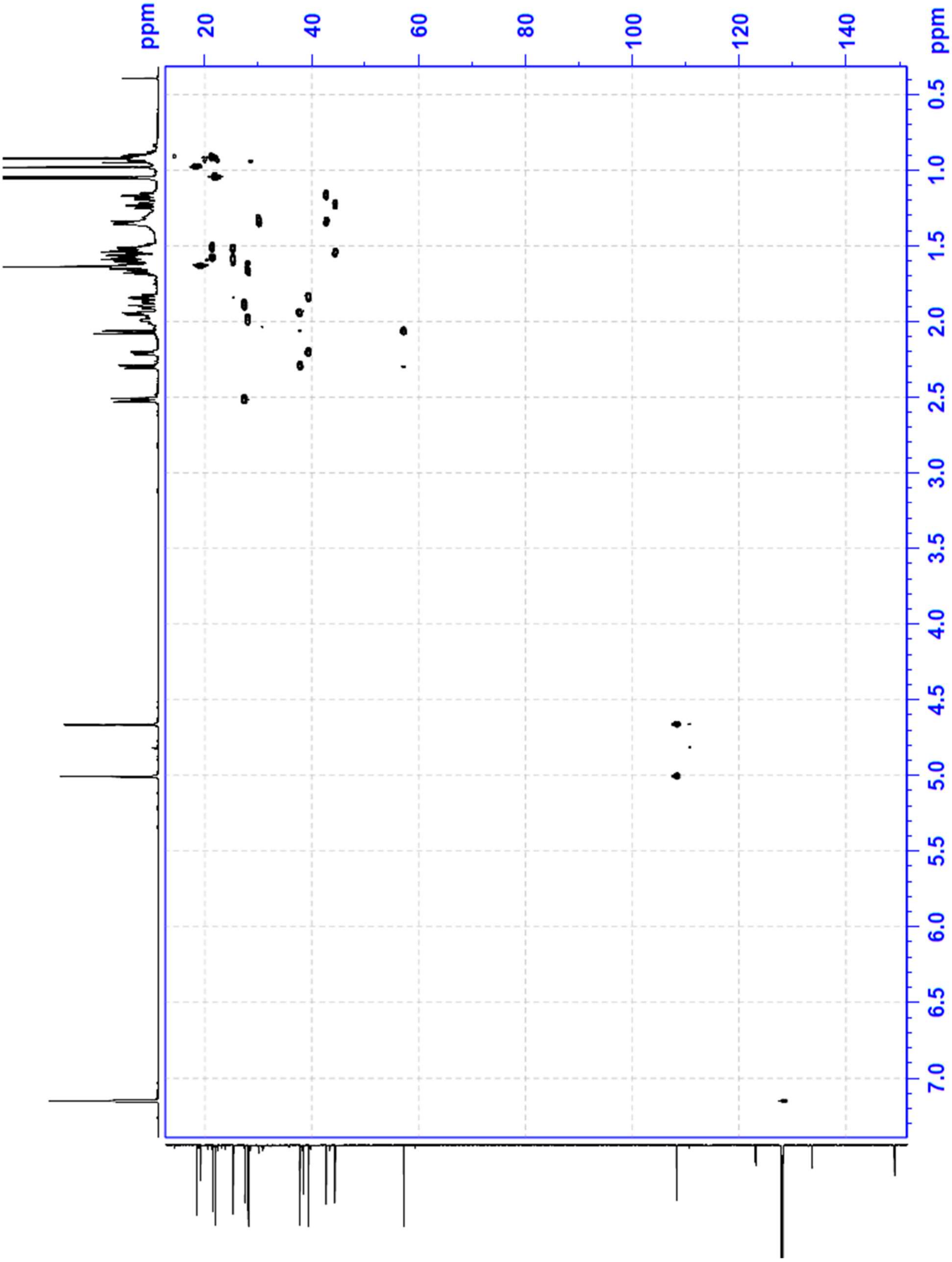

Fig. S11. HSQC spectrum of **1** measured in C_6D_6.

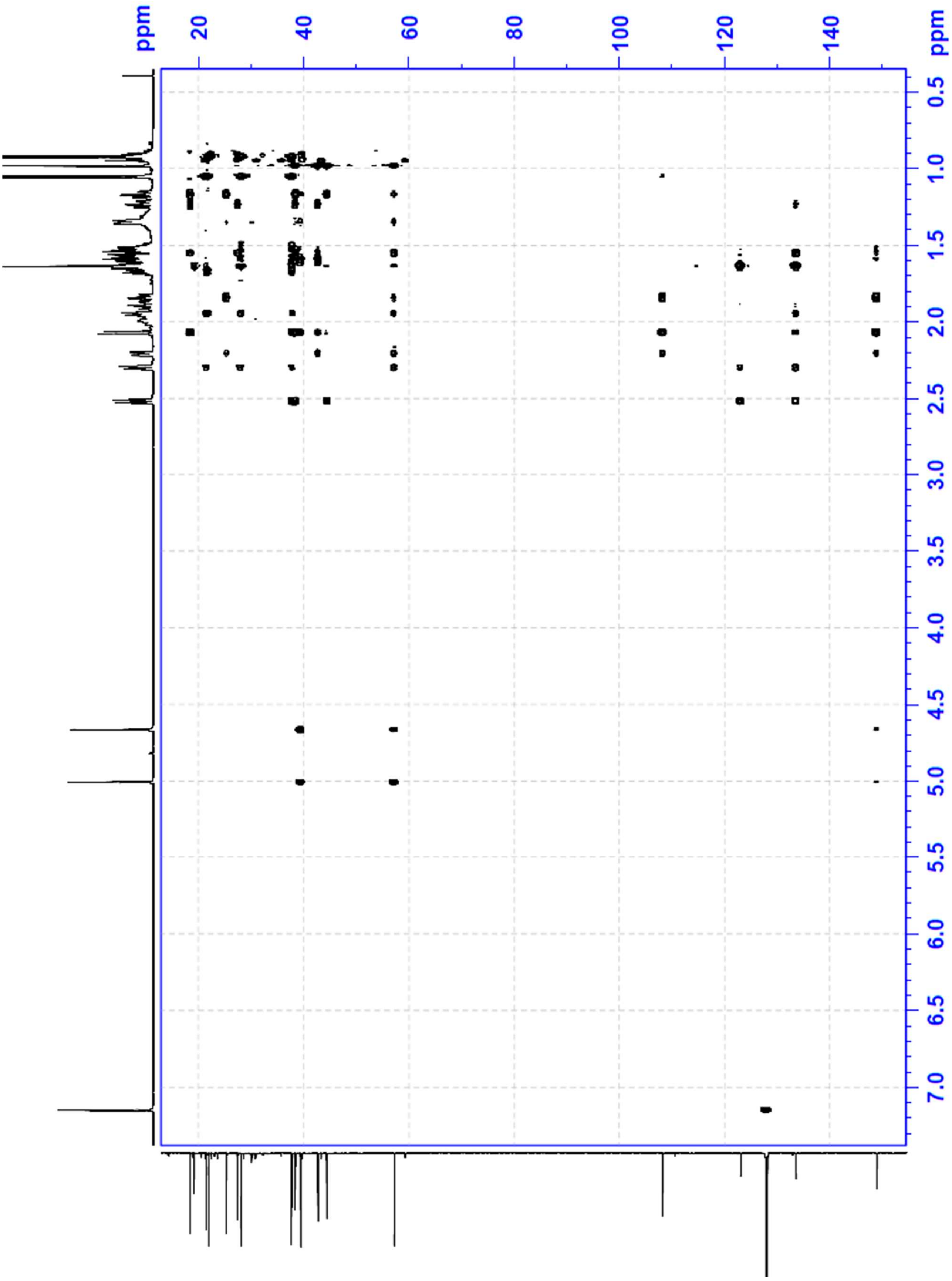

Fig. S12. HMBC spectrum of **1** measured in C_6D_6.

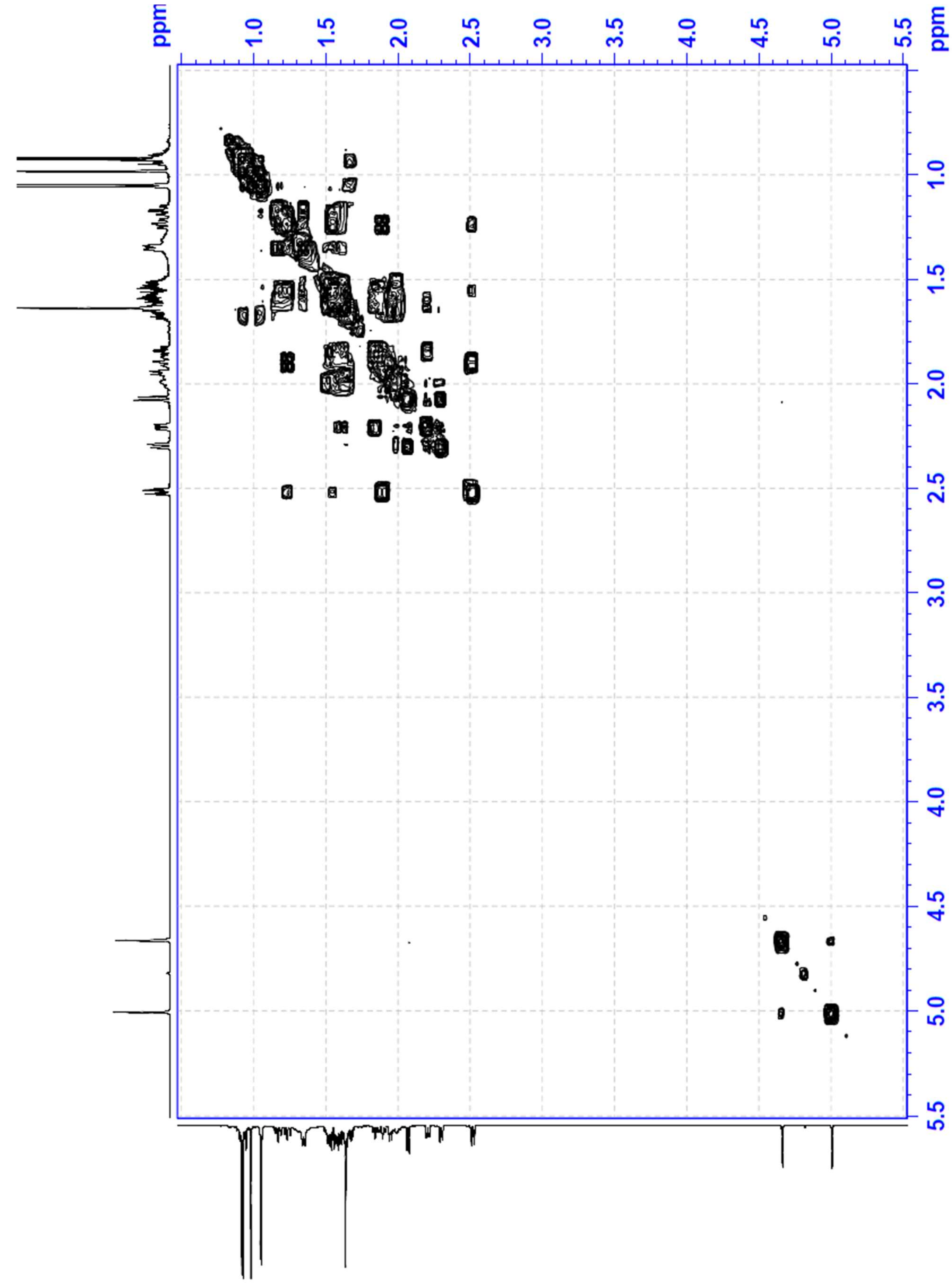

Fig. S13. $^{1}H,^{1}H$-COSY spectrum of **1** measured in C_6D_6.

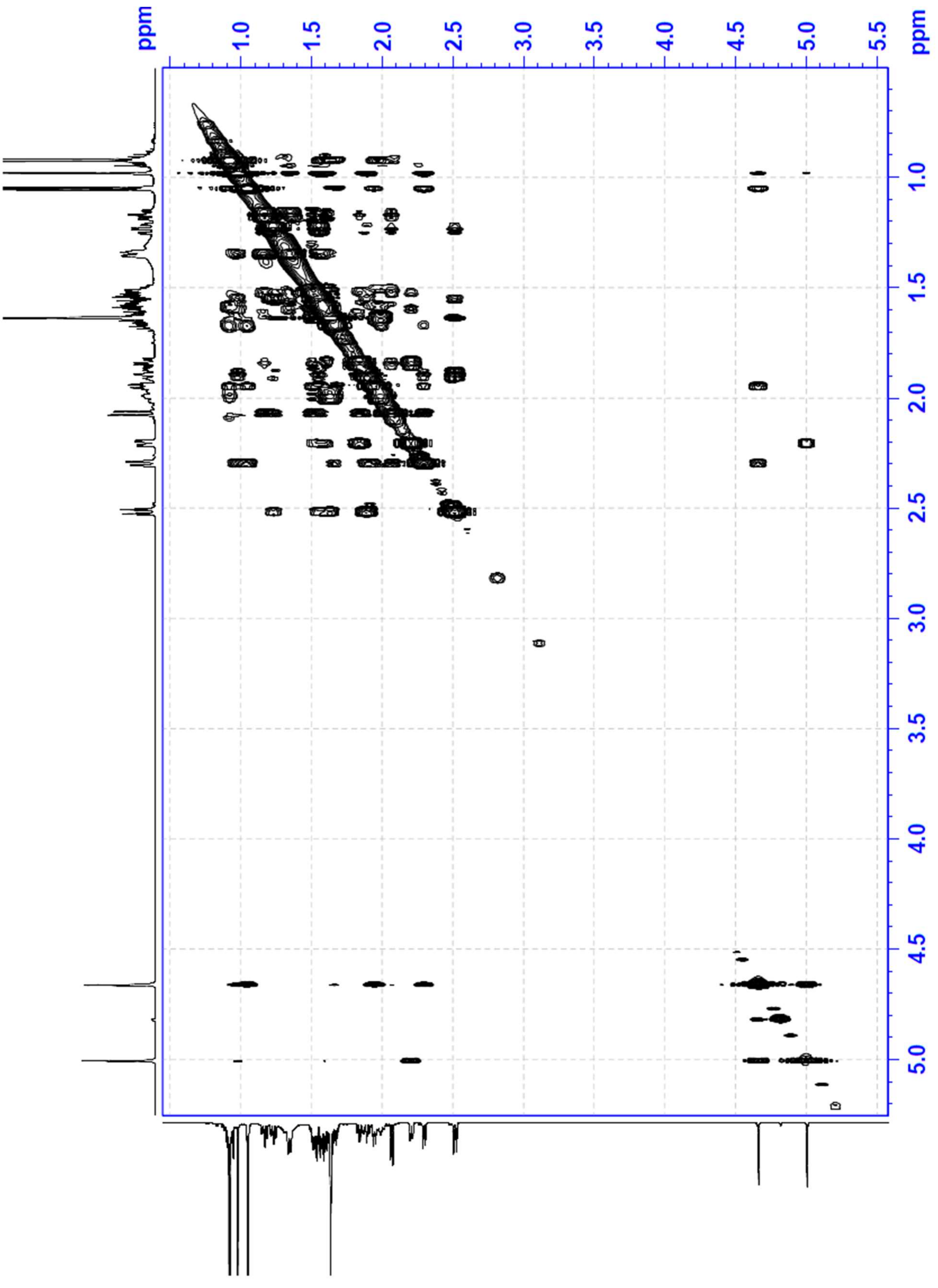

Fig. S14. NOESY spectrum of **1** measured in C_6D_6.

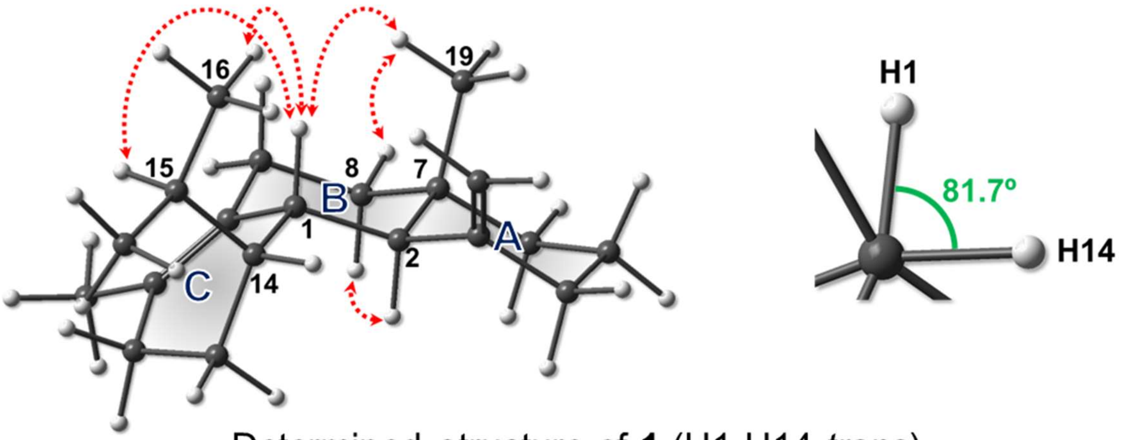

Determined structure of **1** (H1-H14 *trans*)

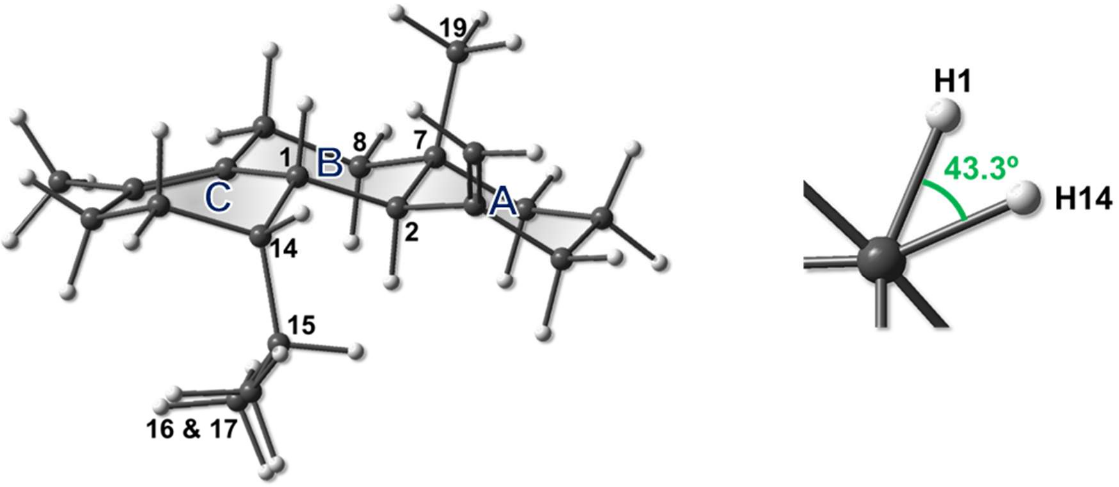

Rejected structure of **1** (H1-H14 *cis*)

Fig. S15. The most stable 3D structures of two isomers of **1** calculated by the conformational search. Two isomers have different stereochemistry at position 14. The structure of H1-H14 *trans* is consisted with the observed NOEs. The dihedral angle of 81.7° in H1-H14 also supports to the observed no H1-H14 correlation in ^{1}H,^{1}H-COSY. The structure of H1-H14 *cis* is rejected because in the structure dihedral angle of H1-H14 is 43.3°, distance between the two hydrogen atoms (H1-H15 and H1-H16) where NOE is observed is too far.

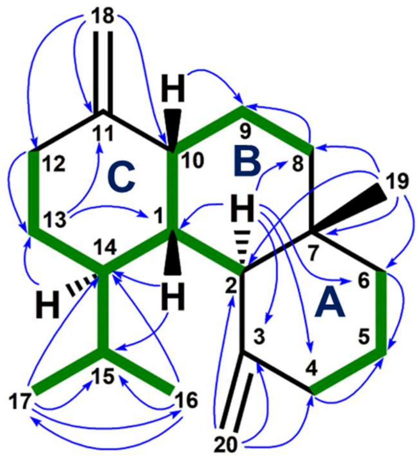

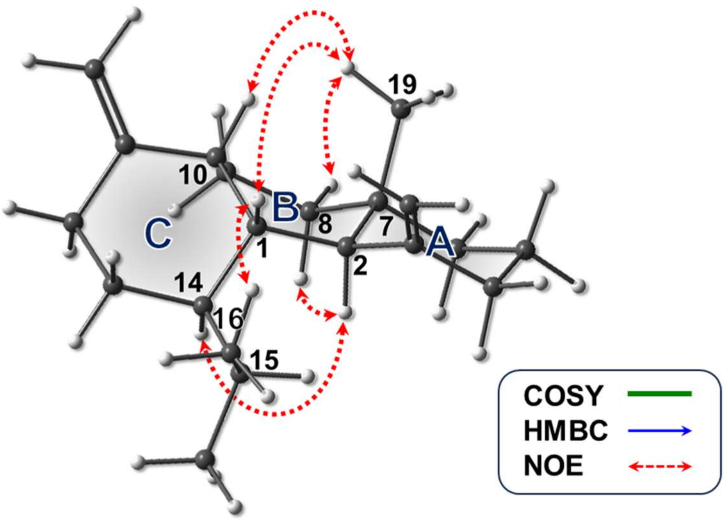

NMR data, δ ppm, in C_6D_6

No.	^{1}H	^{13}C	No.	^{1}H	^{13}C	No.	^{1}H	^{13}C
1	1.37 (1H, m)	43.1 (d)	8	1.16 (m); 1.67 (m)	41.9 (t)	15	2.09 (m)	27.8 (d)
2	1.60 (1H, d, 10.3 Hz)	56.5 (d)	9	1.51 (m); 1.62 (m)	24.0 (t)	16	0.93 (3H, d, 7.0 Hz)	23.5 (q)
3	——	149.7 (s)	10	1.63 (m)	49.7 (d)	17	0.71 (3H, d, 6.5 Hz)	18.1 (q)
4	1.90 (1H, m); 2.19 (1H, m)	39.5 (t)	11	——	153.8 (s)	18	4.75 (1H, s); 4.82 (1H, s)	104.1 (t)
5	1.53 (m); 1.61 (m)	25.8 (t)	12	2.10 (m); 2.41 (1H, m)	36.3 (t)	19	0.76 (3H, s)	20.1 (q)
6	1.16 (m); 1.28 (m)	42.8 (t)	13	1.20 (m); 1.24 (m)	26.9 (t)	20	4.43 (1H, s); 4.79 (1H, s)	106.5 (t)
7	——	38.1 (s)	14	1.20 (m)	52.5 (d)			

Fig. S16. NMR assignment of **2** measured in C_6D_6. The most stable 3D structures calculated by the conformational search are shown on the right.

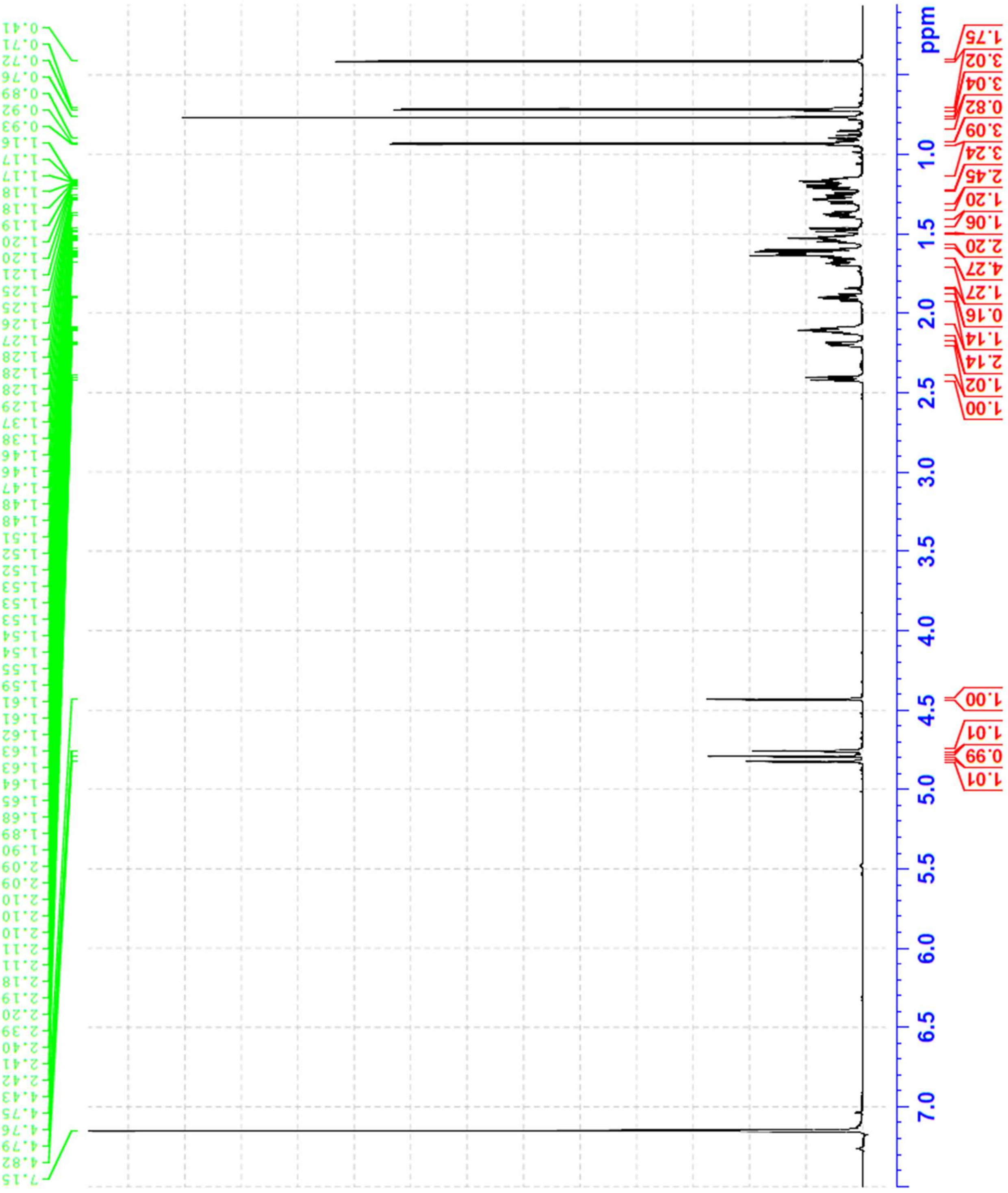

Fig. S17. ^{1}H NMR spectrum of **2** measured in C_6D_6.

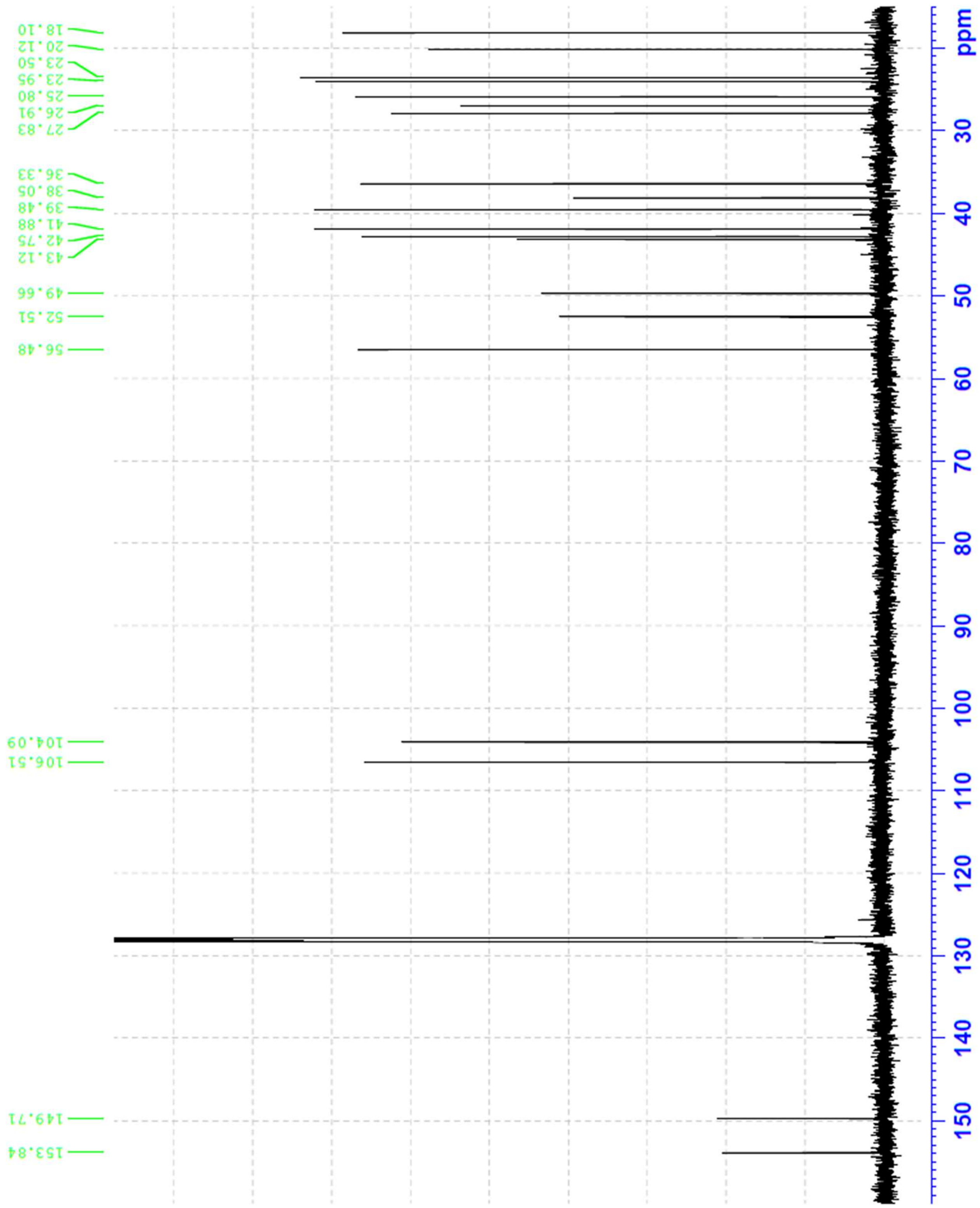

Fig. S18. ^{13}C NMR spectrum of **2** measured in C_6D_6.

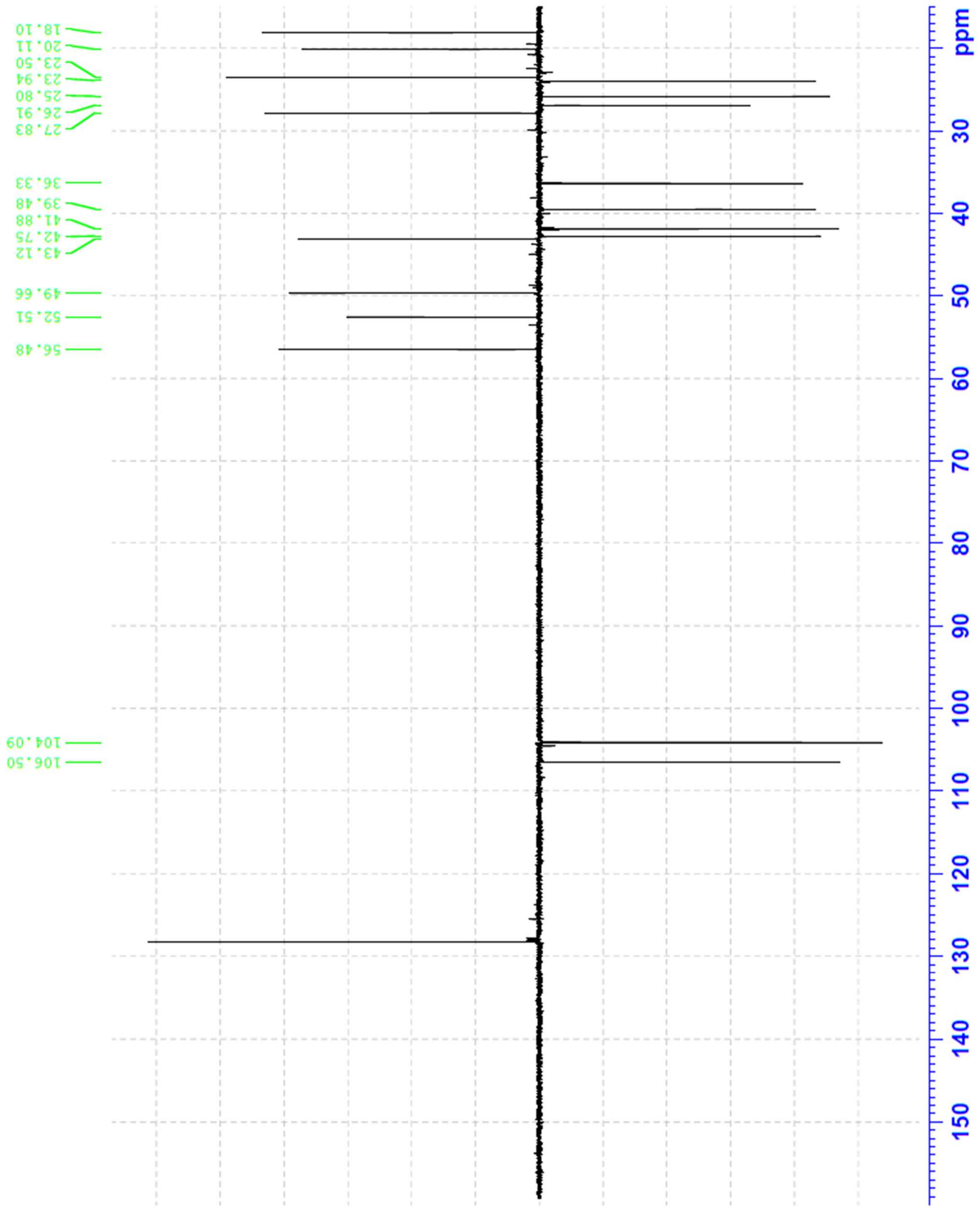

Fig. S19. DEPT135 spectrum of **2** measured in C_6D_6.

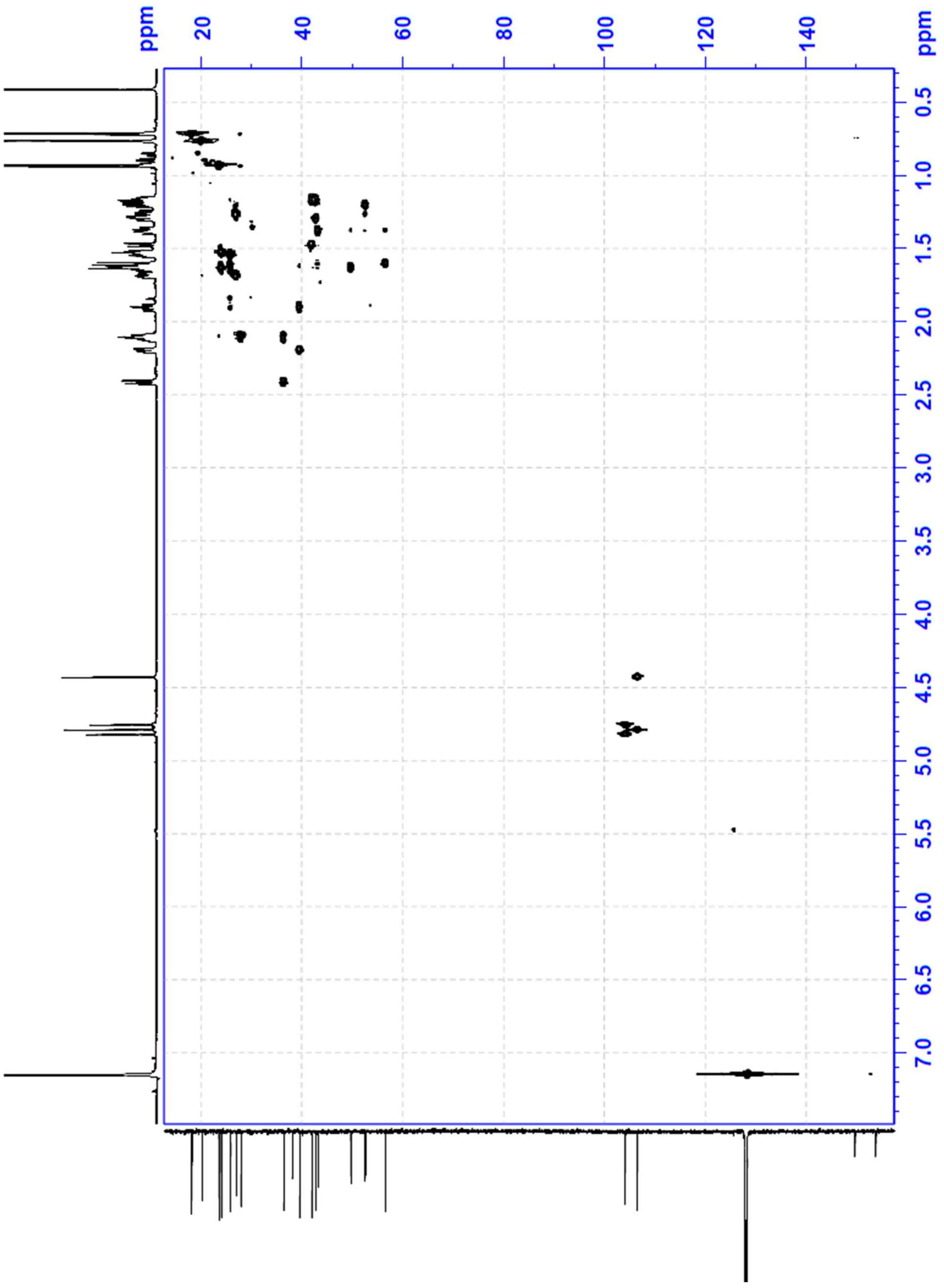

Fig. S20. HSQC spectrum of **2** measured in C_6D_6.

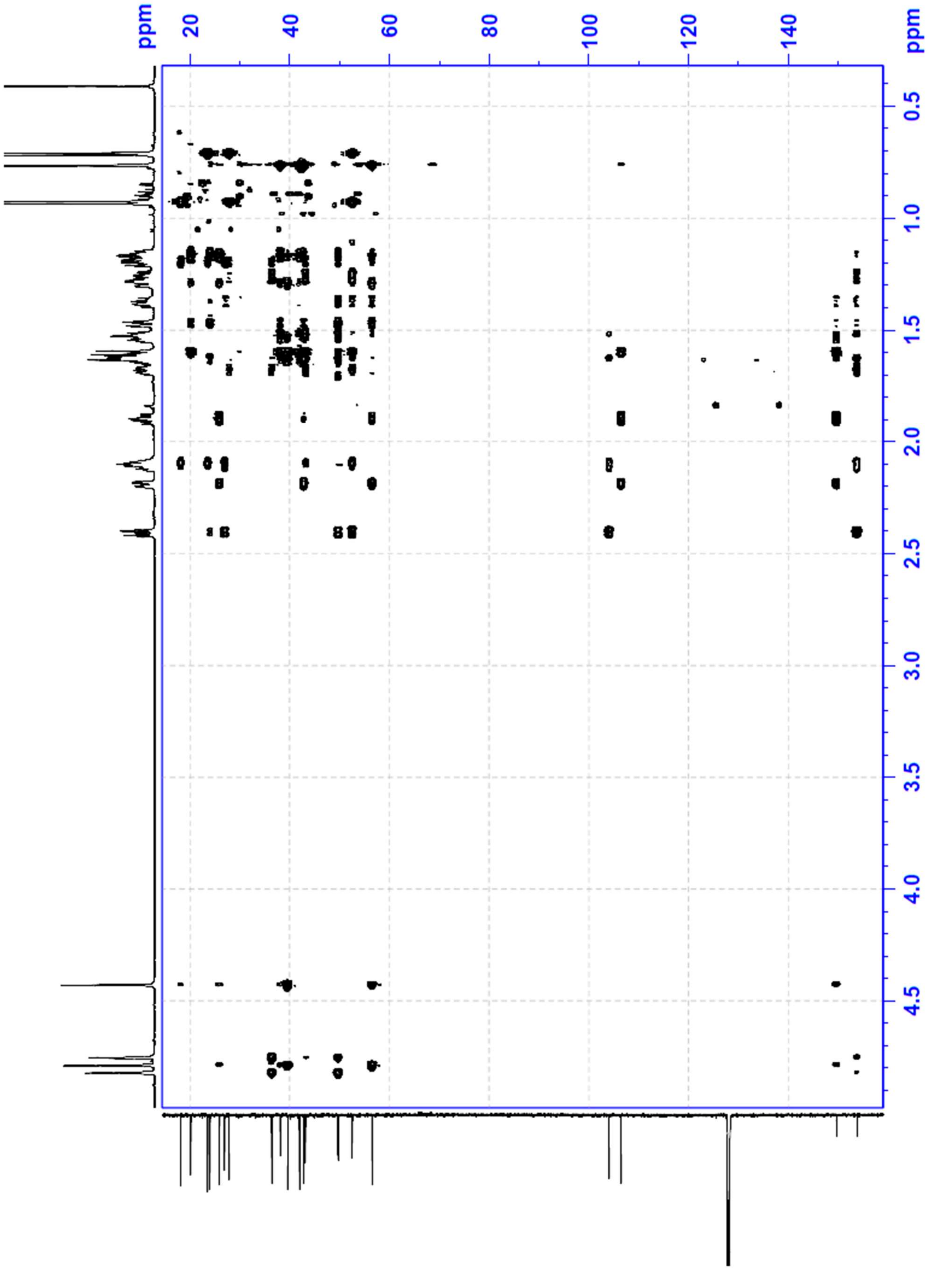

Fig. S21. HMBC spectrum of **2** measured in C_6D_6.

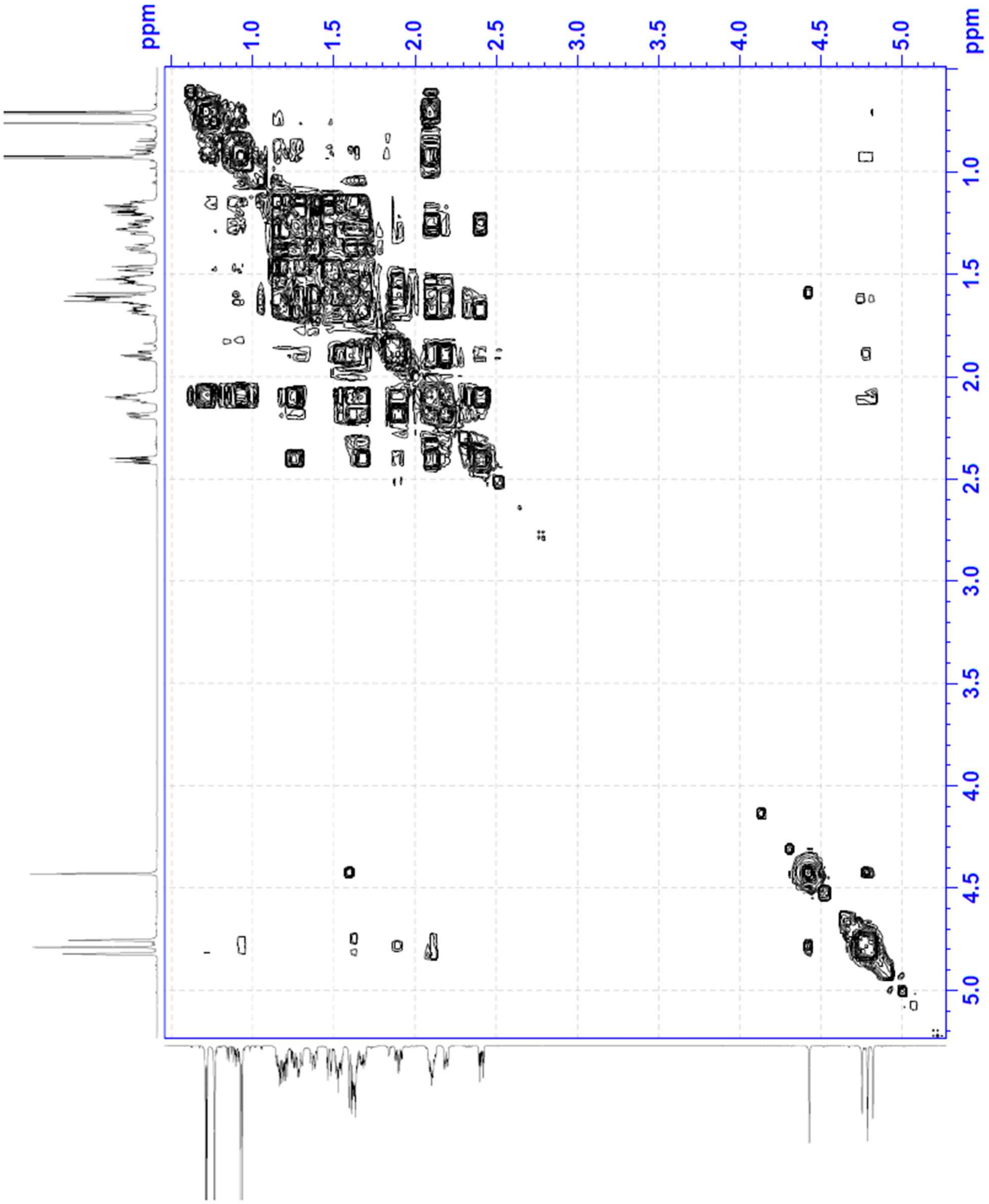

Fig. S22. ^{1}H,^{1}H-COSY spectrum of **2** measured in C_6D_6.

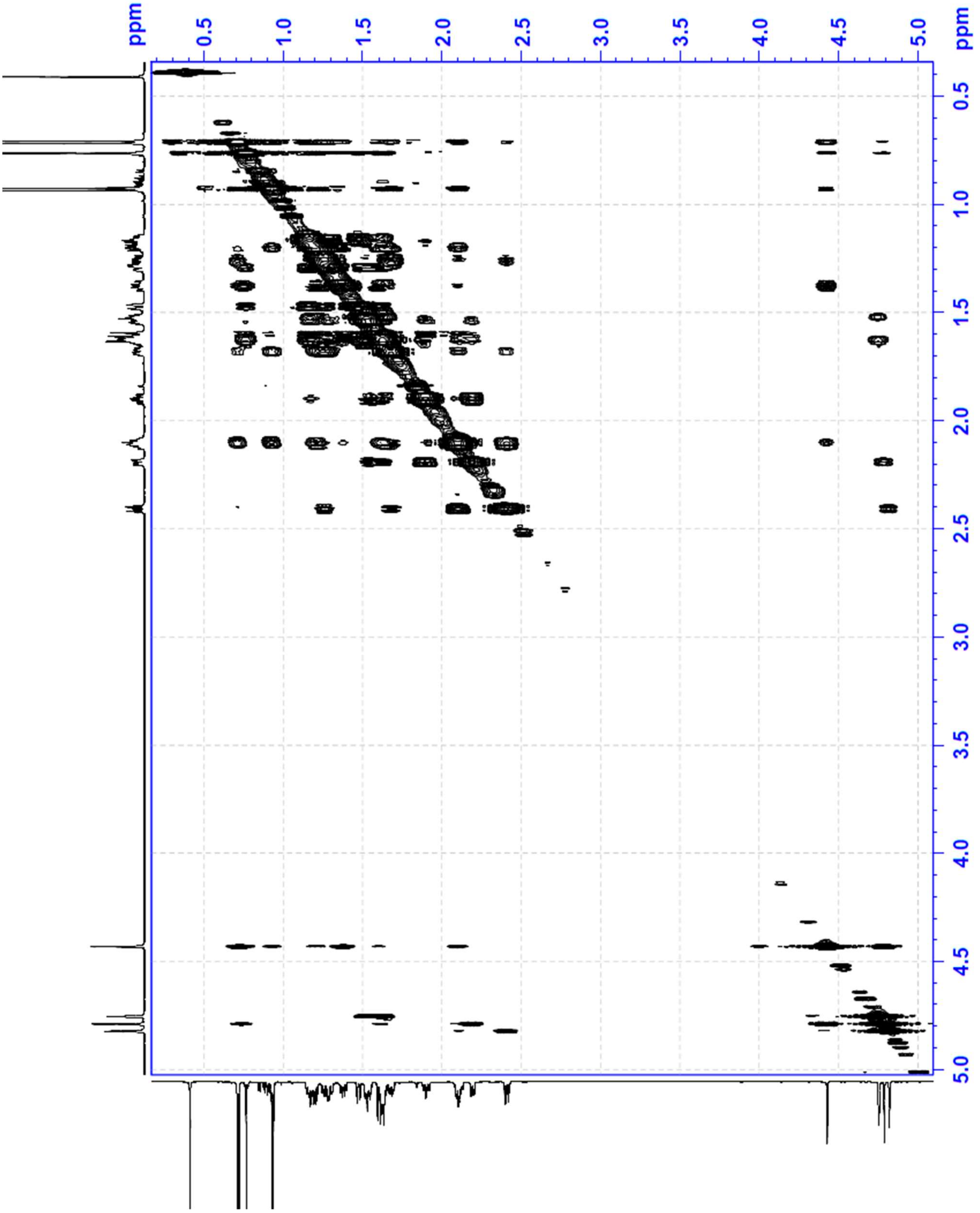

Fig. S23. NOESY spectrum of **2** measured in C_6D_6.

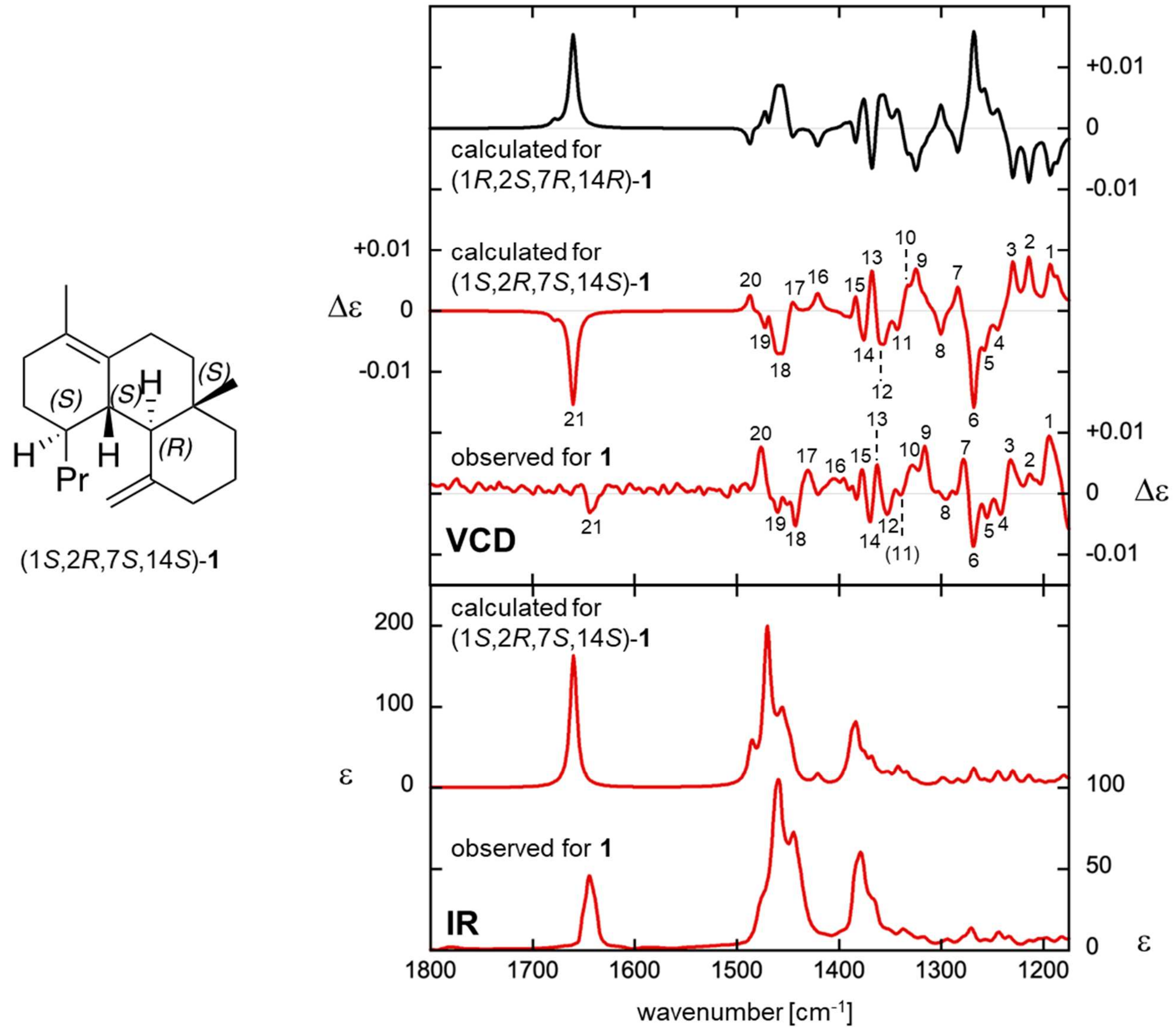

Fig. S24. Calculated and observed VCD/IR spectra of **1**. Corresponding VCD peaks are labelled. Measurement conditions: 0.9 M in $CDCl_3$; l = 50 μm. Calculation conditions: DFT/B3LYP/6-311++G(d,p) with PCM for chloroform. Scaling factor: 0.98.

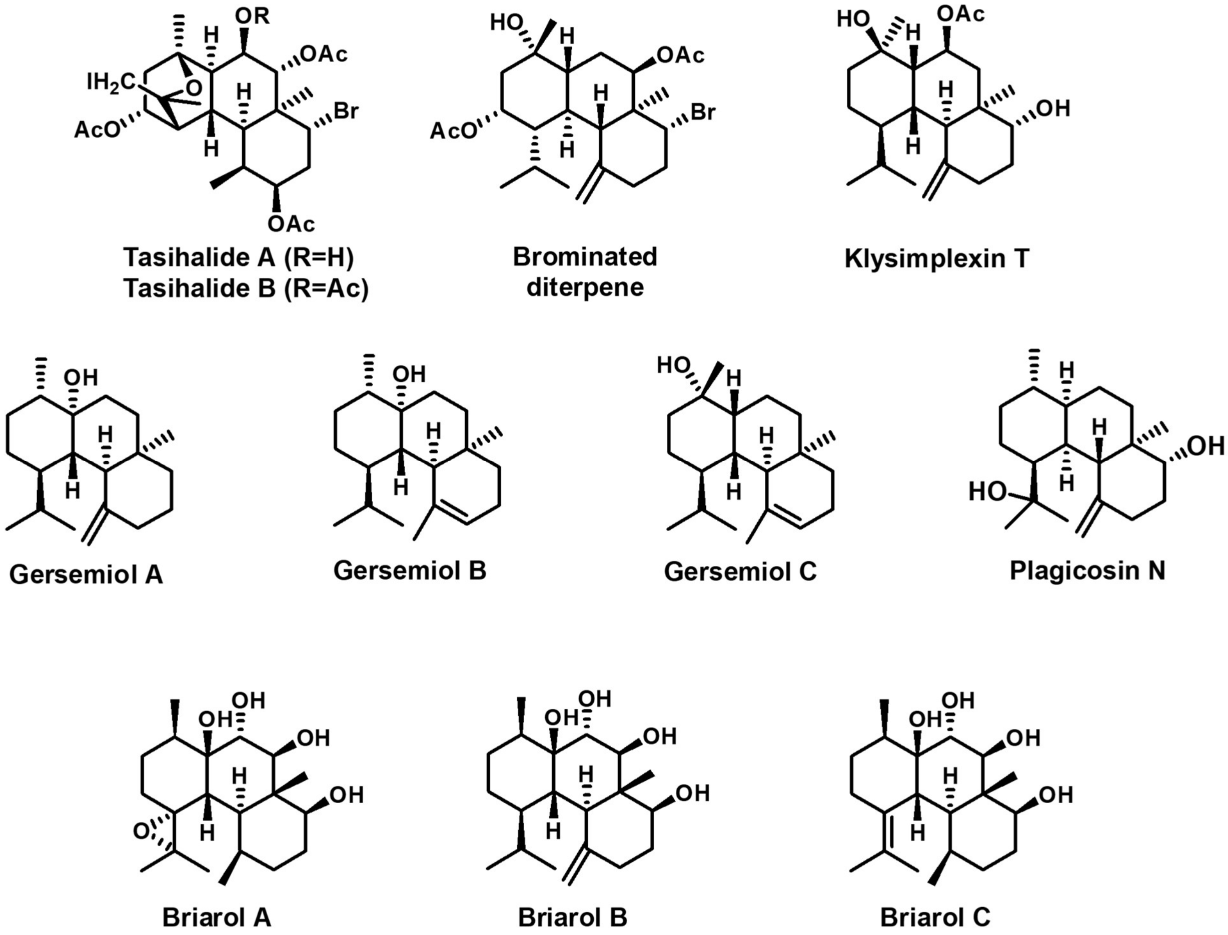

Fig. S25. Examples of natural products possesing gersemiane skeleton.

Tasihalides A and B from the marine cyanobacterium belonging to the genus *Symploca* and the unidentified red alga[22]; Brominated diterpene from the marine angiosperm *Cymodocea nodosa*[23]; Klysimplexin T from the soft coral *Klyxum simplex*[24]; Gersemiols A-C from the soft coral *Gersemia fruticosa*[25]; Plagicosin N from the liverwort *Plagiochila fruticosa*[26]; Briarols A-C from the gorgonian coral *Briareum violaceum*[27].

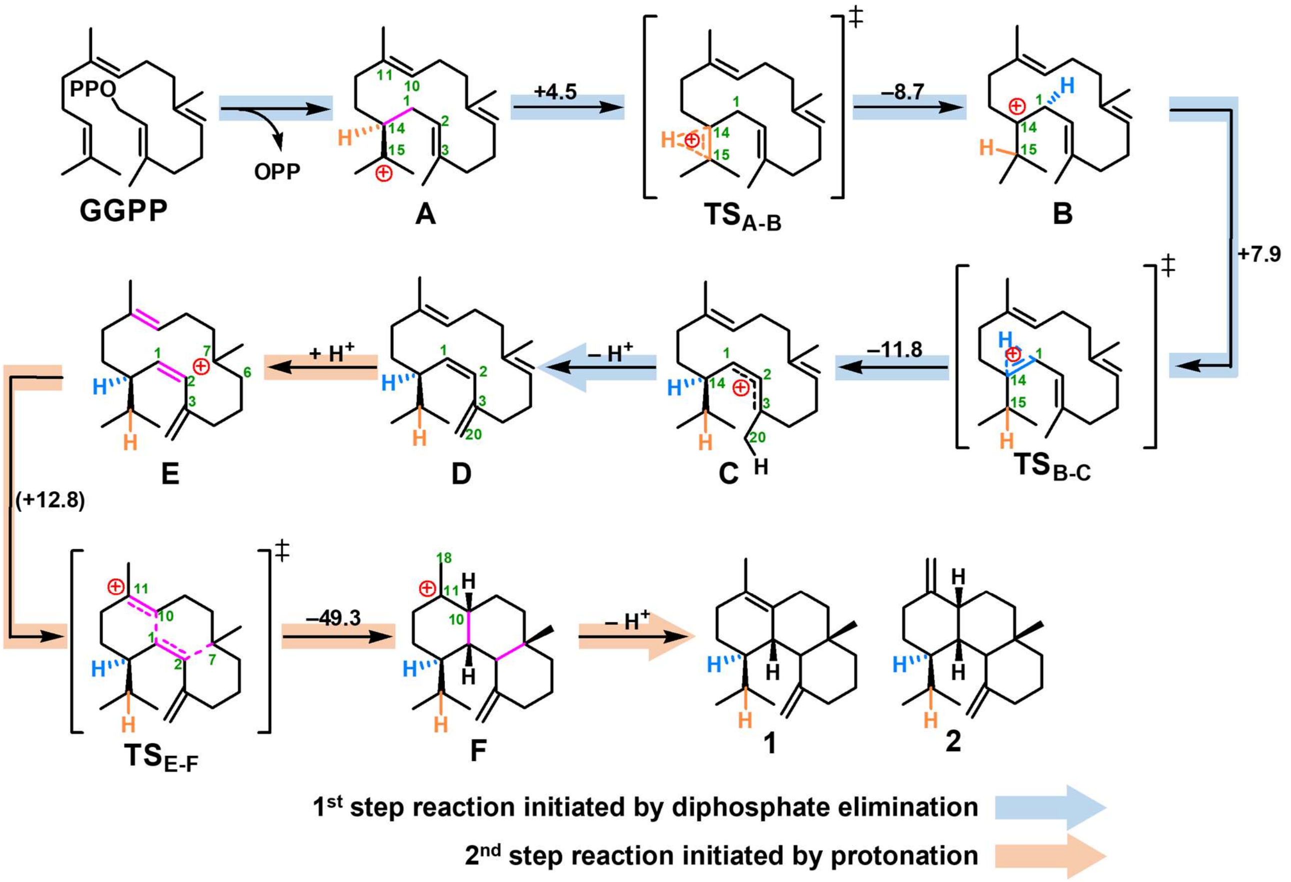

Fig. S26. Proposed mechanism for the formation of PeuTPS products based on the DFT evaluation. Potential energy changes are indicated on the arrows (kcal/mol, Gibbs free energies calculated at M06-2X/6-31+G(d,p).

To investigate the detailed reaction pathways of **1** and **2**, DFT calculations were performed. The C1–C14 cyclization proceeds after dissociation of the diphosphate to form the carbocation **A**. After two successive 1,2-H shifts with relatively low activation energies (4.5 kcal/mol for **TS**$_{\textbf{A-B}}$, 7.9 kcal/mol for **TS**$_{\textbf{B-C}}$), the allyl cation **C** is formed. Subsequently, the intermediate **E** is formed by deprotonation of C20 and protonation of C6. Then, concerted intramolecular annulation forms the tricyclic skeleton **F**. Finally, deprotonation at C10 and C18 gives **1** and **2**, respectively. We propose the above reaction mechanism; however, an alternative pathway to **1** (Fig. S27a) is also possible.

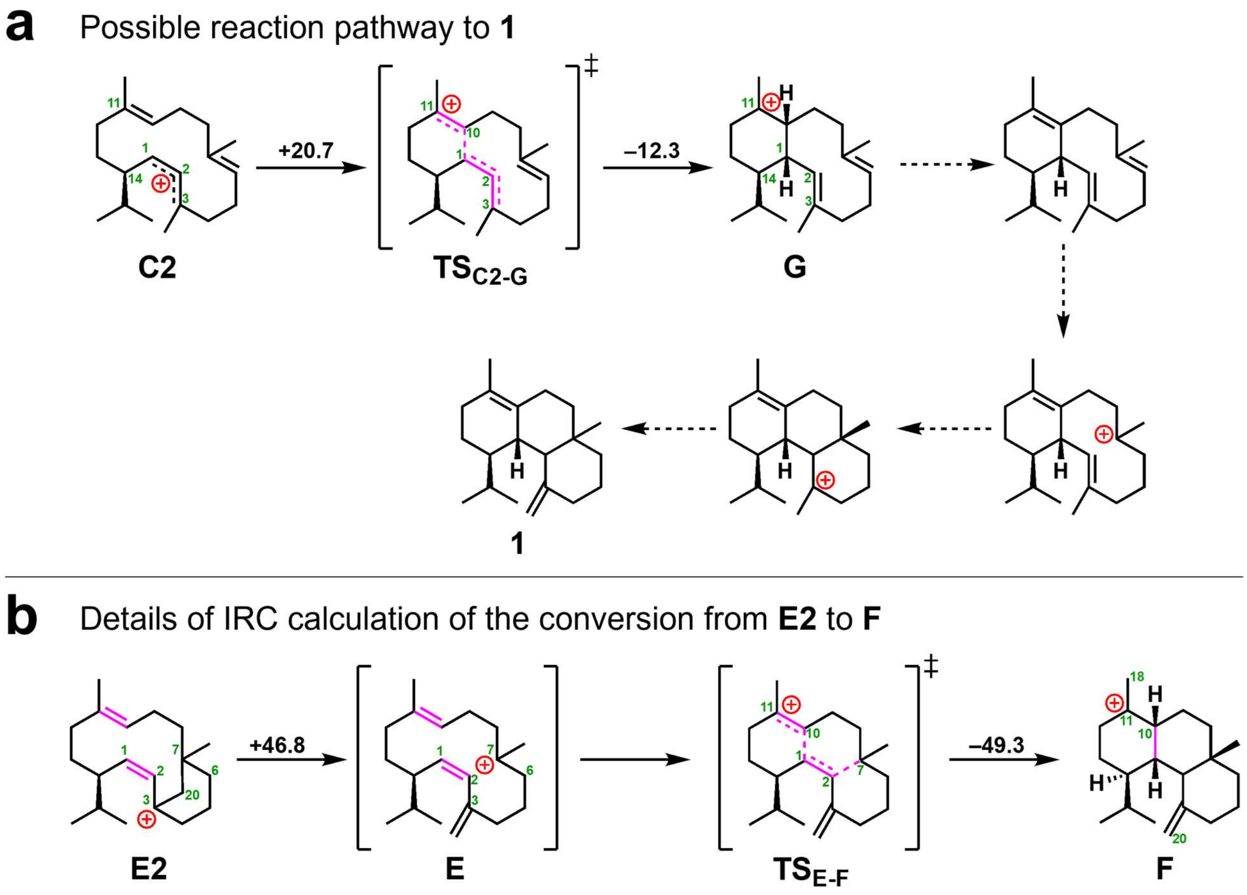

Fig. S27. Mechanistic investigation using DFT calculation.

a) Possible reaction pathway to **1**. Potential energy changes are shown on the arrows (kcal/mol, Gibs free energies calculated at M06-2X/6-31+G(d,p)).

Reaction pathway with C1-C10 bond formation could be possible to yield 6/10 bicyclic structure from carbocation intermediate **C**. However, our calculations indicated that the activation energy requires 20.7 kcal/mol, which is too high to proceed under ambient conditions. Accordingly, this pathway appears not to be the real biosynthetic pathway for **1**. In this calculations, we selected stable conformation of **1** obtained by the conformational search using Spartan'20. Although C1-C10 formation could be possible in a different conformation, however, the conformations of initial substrates and carbocation intermediates are thought to be tightly controlled by the enzyme PeuTPS.

b) Details of IRC calculation of the conversion from **E2** to **F**. Potential energy changes are shown on the arrows (kcal/mol, Gibs free energies calculated at M06-2X/6-31+G(d,p)).

During the IRC calculation of $\mathbf{TS_{E\text{-}F}}$, C–C bond formation from exomethelene C20 towards C7 carbocation was observed, indicating that protonation at C6 in **D** could lead to the **E2** structure. However, the formation **E2** requires the significant conformational changes, which are unlikely to be possible inside the enzyme's active site. Therefore, we have concluded that the enzyme PeuTPS might prevent the formation of **E2** and facilitate the formation of **F**. Accordingly, we picked up the transient structure **E** from the IRC plot and used for the discussion in this study.

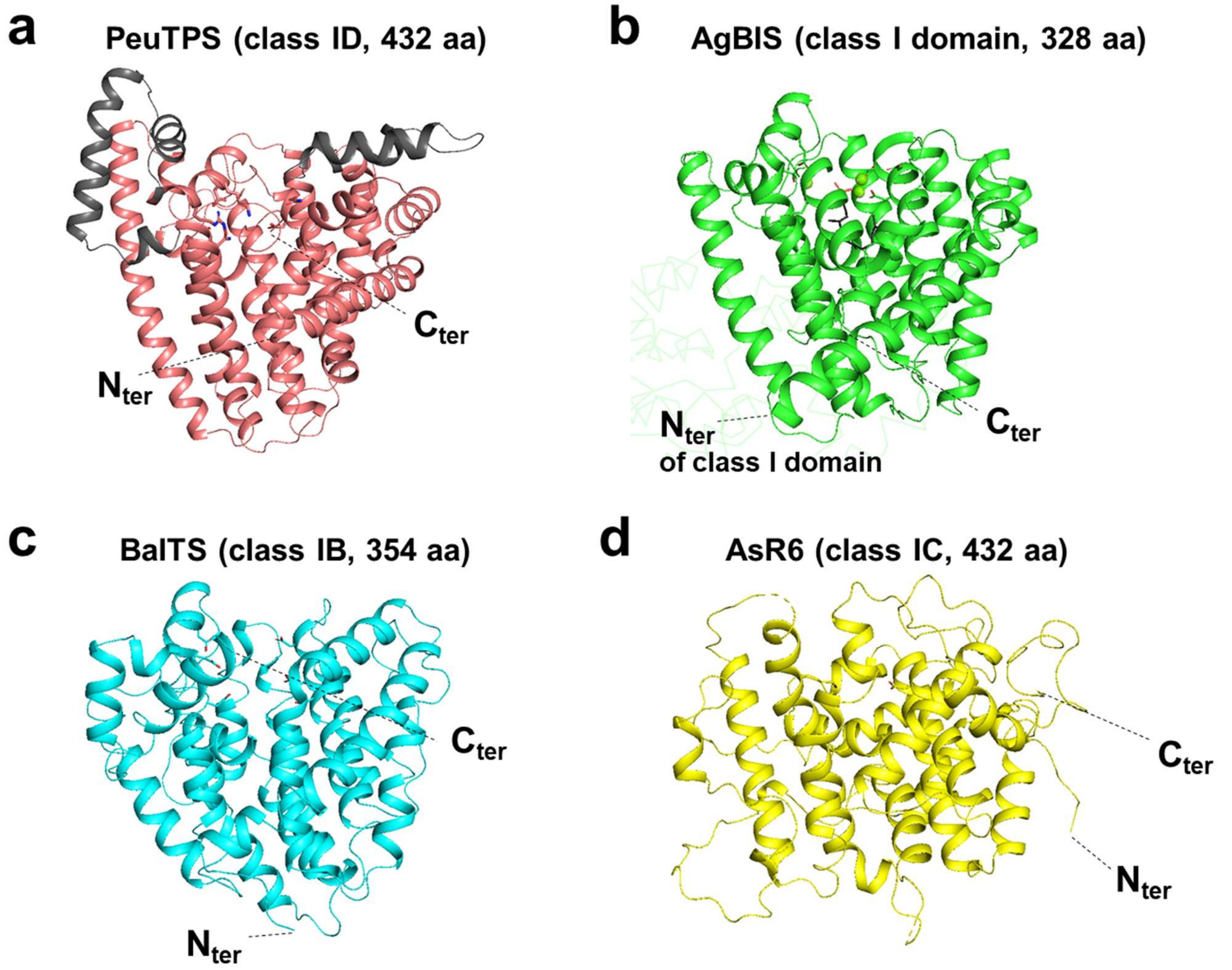

Fig. S28. 3D structures of TPSs. a) Predicted structure of PeuTPS. b) class I TPS domain (E^{489}-L^{817}) of AgBIS (PDB accession: 3sae), and other domains shown as ribbon, c) BalTS (PDB accession: 5yo8), d) AsR6 (PDB accession: 7oc5).

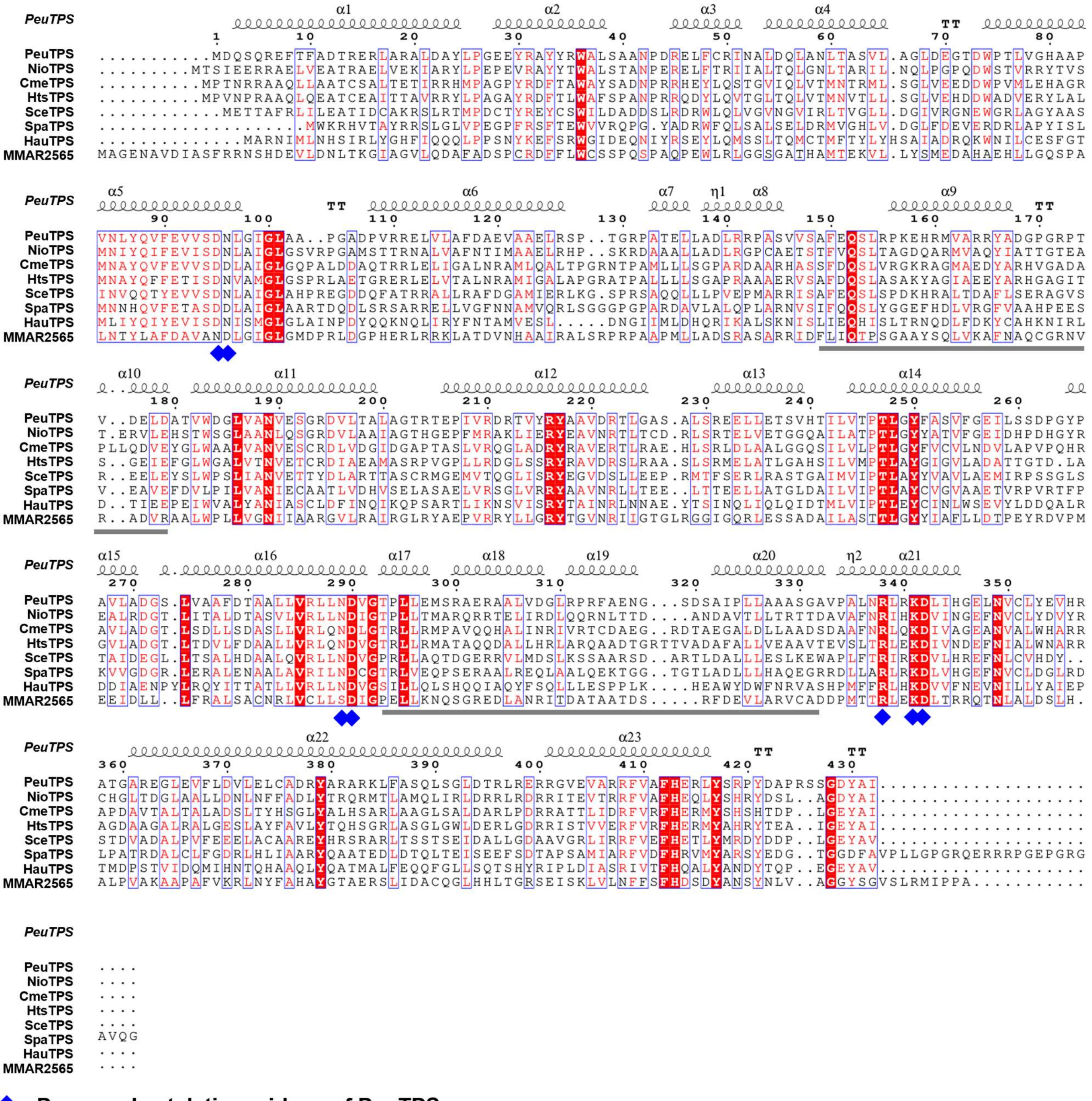

◆ **Proposed catalytic residues of PeuTPS**

▬ **Parts specific to class ID TPSs that do not exist in AgBIS (class I), BalTS (class IB), and AsR6 (class IC)**

Fig. S29. Sequence alignment of class ID TPSs analyzed in this study.

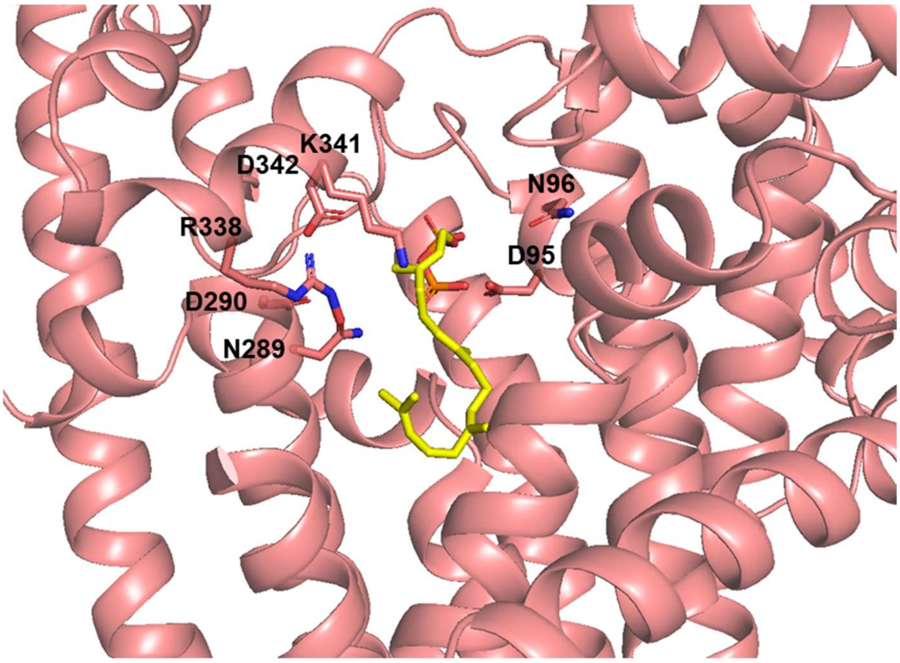

Fig. S30. Docking model of PeuTPS with GGPP. Molecular docking was performed by AutoDock Vina 1.1.2 (https://vina.scripps.edu/). The model with the lowest binding energy was shown as the representative.

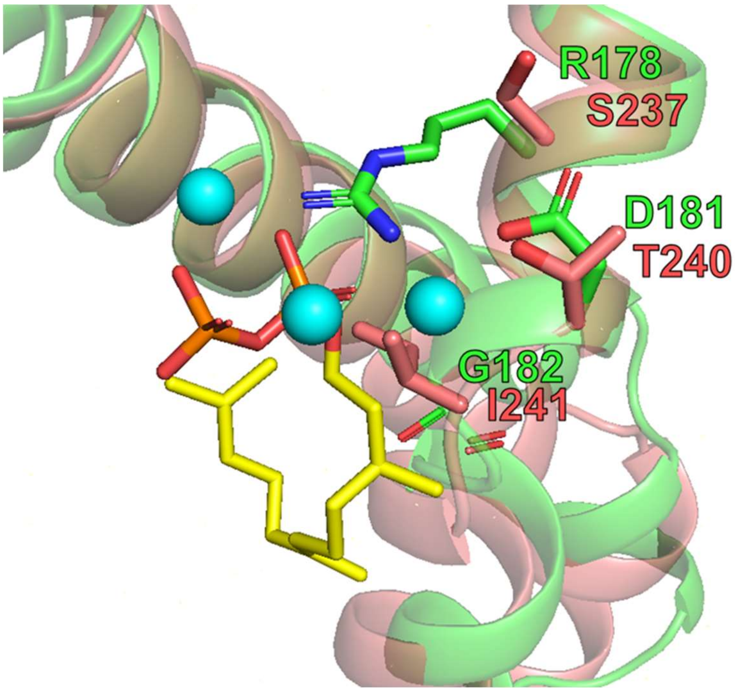

Fig. S31. 3D view of the residues in PeuTPS (pink) corresponding to the effector triad in class I TPS of selinadiene synthase (green, PDB accession: 4okz). Arg178, Asp181, and carbonyl oxygen of Gly182 comprise the diphosphate sensor with the effector triad, which involved in substrate recognition and carbocation stabilization. These residues are strictly conserved in class I TPSs, but have not been found in class ID TPSs in the same position in 3D structure. A side chain of dehydrofarnesyl diphosphate and Mg^{2+} co-crystalized with selinadiene synthase are shown yellow stick and cyan sphere, respectively.

References

1. Jones, P.; Binns, D.; Chang, H. Y.; Fraser, M.; Li, W.; McAnulla, C.; McWilliam, H.; Maslen, J.; Mitchell, A.; Nuka, G.; Pesseat, S.; Quinn, A. F.; Sangrador-Vegas, A.; Scheremetjew, M.; Yong, S. Y.; Lopez, R.; Hunter, S. InterProScan 5: genome-scale protein function classification. *Bioinformatics* **2014**, *30* (9), 1236-1240.
2. Mistry, J.; Chuguransky, S.; Williams, L.; Qureshi, M.; Salazar, G. A.; Sonnhammer, E. L. L.; Tosatto, S. C. E.; Paladin, L.; Raj, S.; Richardson, L. J.; Finn, R. D.; Bateman, A. Pfam: The protein families database in 2021. *Nucleic Acids Res* **2021**, *49* (D1), D412-D419.
3. Varadi, M.; Anyango, S.; Deshpande, M.; Nair, S.; Natassia, C.; Yordanova, G.; Yuan, D.; Stroe, O.; Wood, G.; Laydon, A.; Žídek, A.; Green, T.; Tunyasuvunakool, K.; Petersen, S.; Jumper, J.; Clancy, E.; Green, R.; Vora, A.; Lutfi, M.; Figurnov, M.; Cowie, A.; Hobbs, N.; Kohli, P.; Kleywegt, G.; Birney, E.; Hassabis, D.; Velankar, S. AlphaFold Protein Structure Database: massively expanding the structural coverage of protein-sequence space with high-accuracy models. *Nucleic Acids Res* **2022**, *50* (D1), D439-D444.
4. Holm, L.; Laiho, A.; Törönen, P.; Salgado, M. DALI shines a light on remote homologs: One hundred discoveries. *Protein Sci* **2023**, *32* (1), e4519.
5. Berman, H.; Henrick, K.; Nakamura, H. Announcing the worldwide Protein Data Bank. *Nat Struct Biol* **2003**, *10* (12), 980.
6. Davisson, V. J.; Woodside, A. B; Poulter, C. D.; Synthesis of allylic and homoallylic isoprenoid pyrophosphates. *Methods Enzymol* **1985**, *110* (15), 130–44.
7. Abe, T.; Ozaki, S.; Ueda, D.; Sato, T. Insight into Isoprenoid Biosynthesis by Functional Analysis of Isoprenyl Diphosphate Synthases from Mycobacterium vanbaalenii and Mycobacterium tuberculosis. *Chembiochem* **2020**, *21* (20), 2931-2938.
8. Ueda, D.; Yamaga, H.; Murakami, M.; Totsuka, Y.; Shinada, T.; Sato, T. Biosynthesis of Sesterterpenes, Head-to-Tail Triterpenes, and Sesquarterpenes in Bacillus clausii: Identification of Multifunctional Enzymes and Analysis of Isoprenoid Metabolites. *Chembiochem* **2015**, *16* (9), 1371-1377.
9. Koyama, T.; Fujii, H.; Ogura, K. Enzymatic hydrolysis of polyprenyl pyrophosphates. *Methods Enzymol* **1985**, *110*, 153-155.
10. Sato, T.; Yamaga, H.; Kashima, S.; Murata, Y.; Shinada, T.; Nakano, C.; Hoshino, T. Identification of novel sesterterpene/triterpene synthase from Bacillus clausii. *Chembiochem* **2013**, *14* (7), 822-825.
11. Goto, H.; Obata, S.; Nakayama, N.; Ohta, K. CONFLEX 9, CONFLEX Corporation, Tokyo, Japan, **2021**.
12. Gaussian 16, Revision C.01, Frisch, M. J.; Trucks, G. W.; Schlegel, H. B.; Scuseria, G. E.; Robb, M. A.; Cheeseman, J. R.; Scalmani, G.; Barone, V.; Petersson, G. A.; Nakatsuji, H.; Li, X.; Caricato, M.; Marenich, A. V.; Bloino, J.; Janesko, B. G.; Gomperts, R.; Mennucci, B.; Hratchian, H. P.; Ortiz, J. V.; Izmaylov, A. F.; Sonnenberg, J. L.; Williams-Young, D.; Ding, F.; Lipparini, F.; Egidi, F.; Goings, J.;

Peng, B.; Petrone, A.; Henderson, T.; Ranasinghe, D.; Zakrzewski, V. G.; Gao, J.; Rega, N.; Zheng, G.; Liang, W.; Hada, M.; Ehara, M.; Toyota, K.; Fukuda, R.; Hasegawa, J.; Ishida, M.; Nakajima, T.; Honda, Y.; Kitao, O.; Nakai, H.; Vreven, T.; Throssell, K.; Montgomery, J. A., Jr.; Peralta, J. E.; Ogliaro, F.; Bearpark, M. J.; Heyd, J. J.; Brothers, E. N.; Kudin, K. N.; Staroverov, V. N.; Keith, T. A.; Kobayashi, R.; Normand, J.; Raghavachari, K.; Rendell, A. P.; Burant, J. C.; Iyengar, S. S.; Tomasi, J.; Cossi, M.; Millam, J. M.; Klene, M.; Adamo, C.; Cammi, R.; Ochterski, J. W.; Martin, R. L.; Morokuma, K.; Farkas, O.; Foresman, J. B.; Fox, D. J. Gaussian, Inc., Wallingford CT, 2016.

13. Zhao, Y.; Truhlar, D. G. *Theor. Chem. Acc.* **2008**, *120*, 215–241.
14. Dixit, M.; Weitman, M.; Gao, J.; Major, D. T. *ACS Catal.* **2017**, *7*, 812–818.
15. Zev, S.; Gupta, P. K.; Pahima, E.; Major, D. T. *J. Chem. Theory Comput.* **2022**, *18*, 167–178.
16. Spartan'20 Wavefunction, Inc., Irvine, CA
17. Fukui, K. *Acc. Chem. Res.* **1981**, *14*, 363–368.
18. Page, M.; Doubleday, C.; Jr., J. W. M. *J. Chem. Phys.* **1990**, *93*, 5634–5642.
19. Ishida, K.; Morokuma, K.; Komornicki, A. *J. Chem. Phys.* **1977**, *66*, 2153–2156.
20. Gonzalez, C.; Schlegel, H. B. *J. Phys. Chem.* **1990**, *94*, 5523–5527.
21. Maeda, S.; Ohno, K.; Morokuma, K. *Phys. Chem. Chem. Phys.* **2013**, *15*, 3683–3701.
22. Williams, P. G.; Yoshida, W. Y.; Moore, R. E.; Paul, V. J. Novel iodinated diterpenes from a marine cyanobacterium and red alga assemblage. *Org Lett* **2003**, *5* (22), 4167-4170
23. Kontiza, I.; Stavri, M.; Zloh, M.; Vagias, C.; Gibbons, S.; Roussis, V.; New metabolites with antibacterial activity from the marine angiosperm Cymodocea nodosa. *Tetrahedron* **2008**, *64* (8), 1696-1702.
24. Chen, B. W.; Chao, C. H.; Su, J. H.; Tsai, C. W.; Wang, W. H.; Wen, Z. H.; Huang, C. Y.; Sung, P. J.; Wu, Y. C.; Sheu, J. H. Klysimplexins I-T, eunicellin-based diterpenoids from the cultured soft coral Klyxum simplex. *Org Biomol Chem* **2011**, *9* (3), 834-844.
25. Angulo-Preckler, C.; Genta-Jouve, G.; Mahajan, N.; de la Cruz, M.; de Pedro, N.; Reyes, F.; Iken, K.; Avila, C.; Thomas, O. P. Gersemiols A-C and Eunicellol A, Diterpenoids from the Arctic Soft Coral Gersemia fruticosa. *J Nat Prod* **2016**, *79* (4), 1132-1136.
26. Qiao, Y. N.; Jin, X. Y.; Zhou, J. C.; Zhang, J. Z.; Chang, W. Q.; Li, Y.; Chen, W.; Ren, Z. J.; Zhang, C. Y.; Yuan, S. Z.; Lou, H. X. Terpenoids from the Liverwort Plagiochila fruticosa and Their Antivirulence Activity against Candida albicans. *J Nat Prod* **2020**, *83* (6), 1766-1777.
27. Ahmed, A. F.; Cheng, Y.; Dai, C. F.; Sheu, J. H. Polyoxygenated Klysimplexane- and Eunicellin-Based Diterpenoids from the Gorgonian. *Molecules* **2021**, *26* (11).
